# Deciphering and predicting CD4^+^ T cell immunodominance of influenza virus hemagglutinin

**DOI:** 10.1084/jem.20200206

**Published:** 2020-07-09

**Authors:** Antonino Cassotta, Philipp Paparoditis, Roger Geiger, Ramgopal R. Mettu, Samuel J. Landry, Alessia Donati, Marco Benevento, Mathilde Foglierini, David J.M. Lewis, Antonio Lanzavecchia, Federica Sallusto

**Affiliations:** 1 Institute for Research in Biomedicine, Università della Svizzera italiana, Faculty of Biomedical Sciences, Bellinzona, Switzerland; 2 Institute of Microbiology, ETH Zürich, Zürich, Switzerland; 3 Department of Computer Science, Tulane University, New Orleans, LA; 4 Department of Biochemistry and Molecular Biology, Tulane University School of Medicine, New Orleans, LA; 5 Swiss Institute of Bioinformatics, Lausanne, Switzerland; 6 Surrey Clinical Research Centre, University of Surrey, Guildford, UK

## Abstract

The importance of CD4^+^ T helper (Th) cells is well appreciated in view of their essential role in the elicitation of antibody and cytotoxic T cell responses. However, the mechanisms that determine the selection of immunodominant epitopes within complex protein antigens remain elusive. Here, we used ex vivo stimulation of memory T cells and screening of naive and memory T cell libraries, combined with T cell cloning and TCR sequencing, to dissect the human naive and memory CD4^+^ T cell repertoire against the influenza pandemic H1 hemagglutinin (H1-HA). We found that naive CD4^+^ T cells have a broad repertoire, being able to recognize naturally processed as well as cryptic peptides spanning the whole H1-HA sequence. In contrast, memory Th cells were primarily directed against just a few immunodominant peptides that were readily detected by mass spectrometry–based MHC-II peptidomics and predicted by structural accessibility analysis. Collectively, these findings reveal the presence of a broad repertoire of naive T cells specific for cryptic H1-HA peptides and demonstrate that antigen processing represents a major constraint determining immunodominance.

## Teaser

We explored the nature of the human CD4 T cell immunodominance to influenza H1 hemagglutinin and demonstrate that the naïve repertoire is very broad, while the memory repertoire becomes selected toward peptides with a high likelihood to be processed and presented by dendritic cells.

## Introduction

CD4^+^ T lymphocytes orchestrate adaptive immune responses by secreting cytokines that promote multiple types of inflammatory responses in tissues and by providing help to B cells and CD8^+^ T cells (Sallusto et al., 2010). For antigen recognition, CD4^+^ T cells rely on the interaction with antigen-presenting cells (APCs) that take up, process, and present antigen in the form of short linear peptides bound to MHC class II (MHC-II) molecules (Roche and Furuta, 2015; Unanue et al., 2016). Typically, only a small fraction of the multitude of potentially immunogenic peptides contained in a complex foreign antigen are able to induce a measurable T cell response, with some peptides recognized with higher magnitude and/or frequency and thus arising as immunodominant, and others that remain subdominant or even cryptic (Sercarz et al., 1993; Yewdell and Bennink, 1999; Yewdell and Del Val, 2004).

Given the complexity and tight connection between antigen presentation and recognition, many factors may pertain to peptide and T cell immunodominance. Some of those reflect the biochemical rules of antigen processing and MHC presentation, such as the molecular context in which the peptides are embedded (Graham et al., 2018; Kim and Sadegh-Nasseri, 2015; Landry, 2008; Mirano-Bascos et al., 2008), the affinity of the generated peptides for MHC-II binding, the resistance to HLA-DM–mediated editing of newly formed peptide MHC-II (pMHC-II) complexes (Kim and Sadegh-Nasseri, 2015; Mellins and Stern, 2014), or their kinetic stability on the cell surface of APCs (Sant et al., 2005). Furthermore, the heterogeneous set of proteolytic enzymes and endogenous inhibitors that different kinds of APCs are equipped with (Unanue et al., 2016), as well as the interactions with molecular partners that facilitate antigen uptake, such as B cell receptors (BCRs) or soluble antibodies (Simitsek et al., 1995; Watts and Lanzavecchia, 1993), can affect the antigen processing and the composition of the MHC-II–presented peptidome. Other variables influencing T cell immunodominance depend on the architecture of the T cell repertoires and the mechanisms of antigen recognition (Yewdell, 2006), such as the availability of antigen-specific naive precursors (Jenkins and Moon, 2012; Moon et al., 2007), the interaction affinity of their TCRs with pMHC-II complexes (Malherbe et al., 2004), or the occurrence of TCR cross-reactivity to similar antigenic peptides (Campion et al., 2014; Nelson et al., 2015; Su et al., 2013).

In this study, we chose influenza A virus as a model infectious agent that triggers complex adaptive immune reactions comprising both humoral and cellular responses. Every year, influenza viruses infect more than a billion people worldwide, and are the cause of prominent economic loss as well as significant morbidity and mortality, especially in children <5 yr old and adults >65 (Krammer et al., 2018; Lee et al., 2019; Zens and Farber, 2015). Despite great efforts in research, vaccines are only moderately effective against seasonal strains and are challenged by the rapidly evolving nature of influenza viruses that occasionally emerge as new strains causative of serious epidemics or pandemics (Angeletti and Yewdell, 2018; Krammer et al., 2018; Webster and Govorkova, 2014; Zens and Farber, 2015). We focused our attention on hemagglutinin (HA), which represents the main target of antibody response to influenza virus upon vaccination or infection (Angeletti and Yewdell, 2018; Corti et al., 2011; Krammer et al., 2018; Lee et al., 2019; Pappas et al., 2014). The detailed and unbiased characterization of HA-reactive memory and naive CD4^+^ T cell repertoires, paralleled by a deep analysis of the naturally presented repertoire of MHC-II–binding HA peptides by mass spectrometry (MS)–based immunopeptidomics, allowed us to shed new light on the factors governing CD4^+^ T cell clonal selection and immunodominance to influenza HA in humans.

## Results

### Memory T cells target an immunodominant region of influenza H1-HA

**Figure 1.**
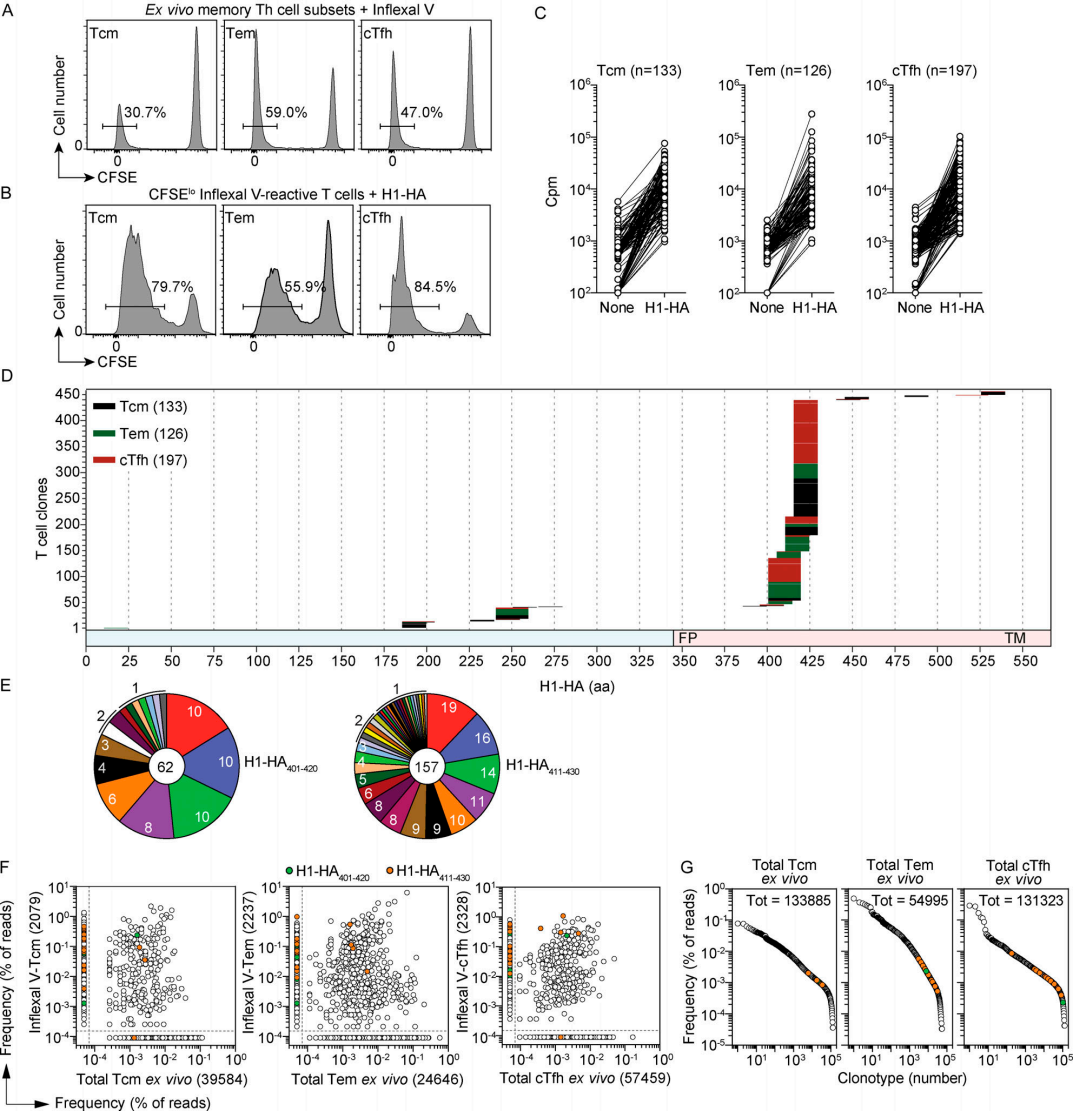
**Clonally expanded memory CD4^+^ T cells target an immunodominant region of influenza H1-HA.** **(A)** Memory CD4^+^ Tcm, Tem, and cTfh cell subsets were isolated from blood samples of donor HD1 6 and 12 mo after Inflexal V vaccination. Cells were labeled with CFSE and stimulated with the Inflexal V vaccine in the presence of autologous monocytes. Shown is the CFSE profile and the percentage of proliferating CFSE^lo^ cells on day 6 in the 12-mo sample. Percentage of CFSE^lo^ in the 6-mo sample was 38% (Tcm), 65% Tem, and 57% cTfh. **(B)** The Inflexal V–reactive CFSE^lo^ T cells were sorted, relabeled with CFSE, and stimulated with recombinant H1-HA in the presence of autologous monocytes. After 5 d, CFSE^lo^ proliferating T cells were sorted and cloned by limiting dilution. Shown is the experiment performed with the 12-mo sample; comparable results were obtained with the 6-mo sample. **(C)** A total of 456 H1-HA–specific CD4^+^ T cell clones were isolated from the Tcm, Tem, and cTfh cultures based on the proliferative response (stimulation index ≥3) to a pool of overlapping peptides spanning the entire H1-HA sequence. Proliferation was assessed on day 3 after a 16-h pulse with [^3^H]thymidine and expressed as counts per minute. The data are representative of at least two independent experiments. **(D)** Epitope mapping of the 456 H1-HA–specific T cell clones. Epitopes were identified by screening the T cell clones with the individual H1-HA peptides in at least three independent experiments, with consistent results. The x axis indicates H1-HA amino acid sequence; each color-coded segment represents the sequence recognized by individual clones isolated from Tcm (black), Tem (green), or cTfh (red) cultures. The numbers of H1-HA–reactive T cell clones isolated from each subset are reported. The chart at the bottom indicates HA1 and HA2 domains colored in blue and red, respectively (FP, fusion peptide; TM, transmembrane). **(E)** Rearranged TCR Vβ sequences of H1-HA–specific T cell clones were determined by RT-PCR followed by Sanger sequencing. Shown in the pie charts are the repertoires of rearranged TCR Vβ sequences of T cell clones recognizing immunodominant H1-HA_401–420_ and H1-HA_411–430_ epitopes. Each slice of the chart indicates a different TCR Vβ clonotype (H1-HA_401–420_, *n* = 16; H1-HA_411–430_, *n* = 39); the number of sister clones bearing the same TCR Vβ sequence are reported for each slice. The total number of clones sequenced is reported at the center. **(F and G)** To evaluate the clonal expansion of H1-HA–reactive memory T cell clones, TCR Vβ deep sequencing was performed on Inflexal V–reactive CFSE^lo^ Tcm, Tem, and cTfh cells and on ex vivo total Tcm, Tem, and cTfh cells isolated from the same blood samples and immediately sequenced. Comparison of TCR Vβ clonotype frequency distribution of total (x axis) and Inflexal V–reactive cells (y axis) is shown in F. Dots outside the dashed lines represent clonotypes that were found in only one of the two samples and that were assigned an arbitrary frequency value for graphical purposes. **(G)** The frequency distribution of TCR Vβ clonotypes from ex vivo total Tcm, Tem, and cTfh cells isolated from a blood sample of donor HD1 obtained 48 mo after the 2013/14 Inflexal V vaccination. TCR Vβ clonotypes recognizing the immunodominant H1-HA_401–420_ or H1-HA_411–430_ peptides found in any of the samples analyzed are colored in green and orange, respectively.

**Figure S1.**
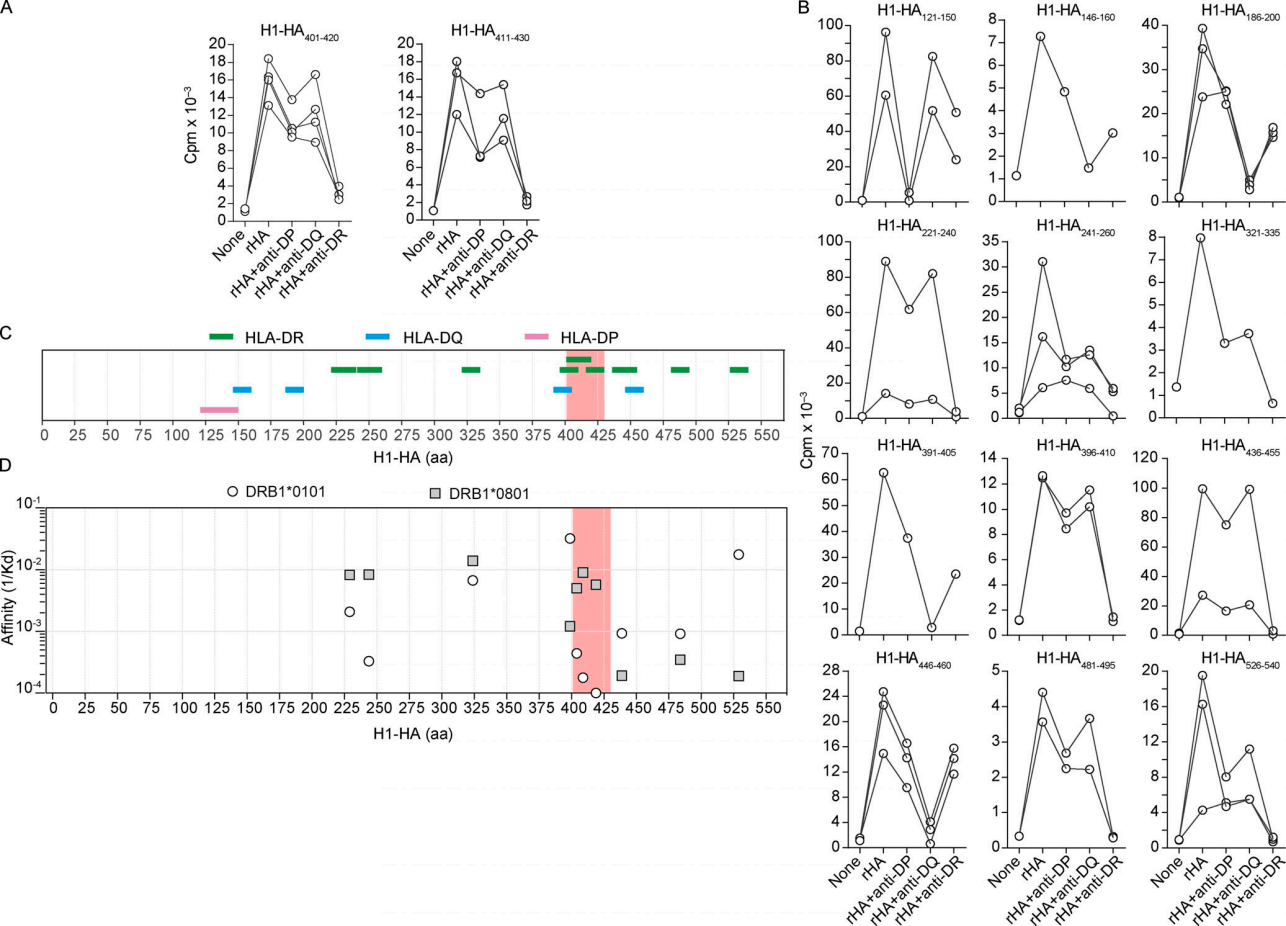
**MHC restriction of H1-HA–specific T cell clones and measurement of peptide binding affinity to HLA-DR. (A** **and** **B)** MHC-II restriction of T cell clones from donor HD1 specific for different H1-HA peptides was determined by stimulation with autologous APCs pulsed with recombinant H1-HA, in the absence or presence of anti–MHC-II–blocking antibodies (anti–HLA-DR, clone L243; anti–HLA-DQ, clone SPVL3; anti–HLA-DP, clone B7/21). Proliferation was assessed on day 3 after a 16-h pulse with [^3^H]thymidine and expressed as counts per minute. MHC-II restriction was defined based on inhibition of T cell proliferation >80%. Shown are representative (30 of 85 analyzed) T cell clones specific for immunodominant (A) or subdominant (B) H1-HA epitopes. Data are grouped based on the epitope specificity of the T cell clones tested, reported on top of each plot. **(C)** Summary of MHC-II restriction of H1-HA–specific T cell clones isolated from donor HD1. The x axis indicates H1-HA amino acid sequence; each color-coded segment represents the peptide recognized by T cell clones restricted by HLA-DP (pink), HLA-DQ (blue), or HLA-DR (green). The immunodominant H1-HA_401–430_ region identified in the memory compartment of donor HD1 is highlighted with a red shadow. **(D)** MHC-II binding affinity of H1-HA peptides recognized by HLA-DR–restricted T cell clones from donor HD1 was measured in vitro. Briefly, recombinant HLA-DRB1 isoforms were refolded in the presence of recombinant HLA-DRA and increasing concentration of peptides, at room temperature and pH 7. *K_d_* values were calculated by nonlinear regression fitting of pMHC-II refolding curves. The plot reports the inverse *K_d_* values of each H1-HA peptide tested with either HLA-DRB1*01:01 (white dots) or HLA-DRB1*08:01 (gray squares) molecules. The immunodominant H1-HA_401–430_ region identified in the memory compartment is highlighted with a red shadow.

To capture the entire repertoire of memory T cells specific for influenza HA, we obtained multiple and large blood samples from a donor (HD1) after vaccination with the 2013/14 seasonal Inflexal V vaccine containing HA from the pandemic A/California/07/2009 H1N1 strain (H1-HA). Central memory (Tcm), effector memory (Tem), and circulating follicular helper (cTfh) CD4^+^ T cells were isolated by cell sorting, labeled with CFSE, and stimulated with Inflexal V. When analyzed on day 6, proliferating CFSE^lo^ T cells were detected in all three memory subsets from samples obtained 6 and 12 mo after vaccination (Fig. 1 A). To select H1-HA–reactive T cells, the CFSE^lo^ T cells were sorted, relabeled with CFSE, and stimulated with H1-HA (Fig. 1 B). T cells proliferating in the secondary stimulation were cloned, and 456 H1-HA–specific clones were isolated (Fig. 1 C and Table S1) and characterized for peptide specificity, MHC restriction (HLA-DR, HLA-DP, or HLA-DQ), and TCR Vβ usage. Strikingly, >85% of the clones isolated (393 of 456) recognized two overlapping peptides (H1-HA_401–420_ or H1-HA_411–430_; Fig. 1 D), thus defining, in this individual, a highly immunodominant region. T cells specific for the immunodominant H1-HA_401–430_ region were found in all three memory subsets (94 clones in Tcm, 112 clones in Tem, and 187 clones in cTfh) and were HLA-DR restricted, as shown by antibody blocking experiments (Fig. S1 A). Several T cell clones specific for subdominant H1-HA regions were also HLA-DR restricted, with a minority being HLA-DQ or HLA-DP restricted (Fig. S1, B and C).

TCR Vβ Sanger sequencing performed on 274 T cell clones showed that the response to H1-HA was highly polyclonal, comprising 88 distinct clonotypes, even when directed against the immunodominant region (Table S2). For instance, 62 T cell clones specific for the immunodominant peptide H1-HA_401–420_ comprised 16 different clonotypes, and 157 T cell clones specific for the immunodominant peptide H1-HA_411–430_ comprised 39 different clonotypes (Fig. 1 E and Table S2). Of note, 26 of the 39 H1-HA_411–430_-specific clonotypes used the TRBV19 gene, suggesting a preferential TCR CDR1 and CDR2 usage that might facilitate cognate interaction with the peptide–MHC complex.

Tracking of H1-HA–reactive T cell clonotypes within the CFSE^lo^ T cell population responding to Inflexal (Fig. 1 A) showed that those against the immunodominant H1-HA region were among the most represented and that some were also found in the Tcm, Tem, or Tfh repertoire ex vivo (Fig. 1 F). Strikingly, several of these clonotypes were still detected in memory T cell subsets isolated from donor HD1 48 mo later (Fig. 1 G). Collectively, these findings indicate that in an Inflexal-immunized donor, a polyclonal repertoire of H1-HA–specific Tcm, Tem, and cTfh cells is highly focused on a small immunodominant region.

### Memory T cells are focused against immunodominant regions, while naive T cells recognize multiple peptides spanning the entire H1-HA sequence

**Figure 2.**
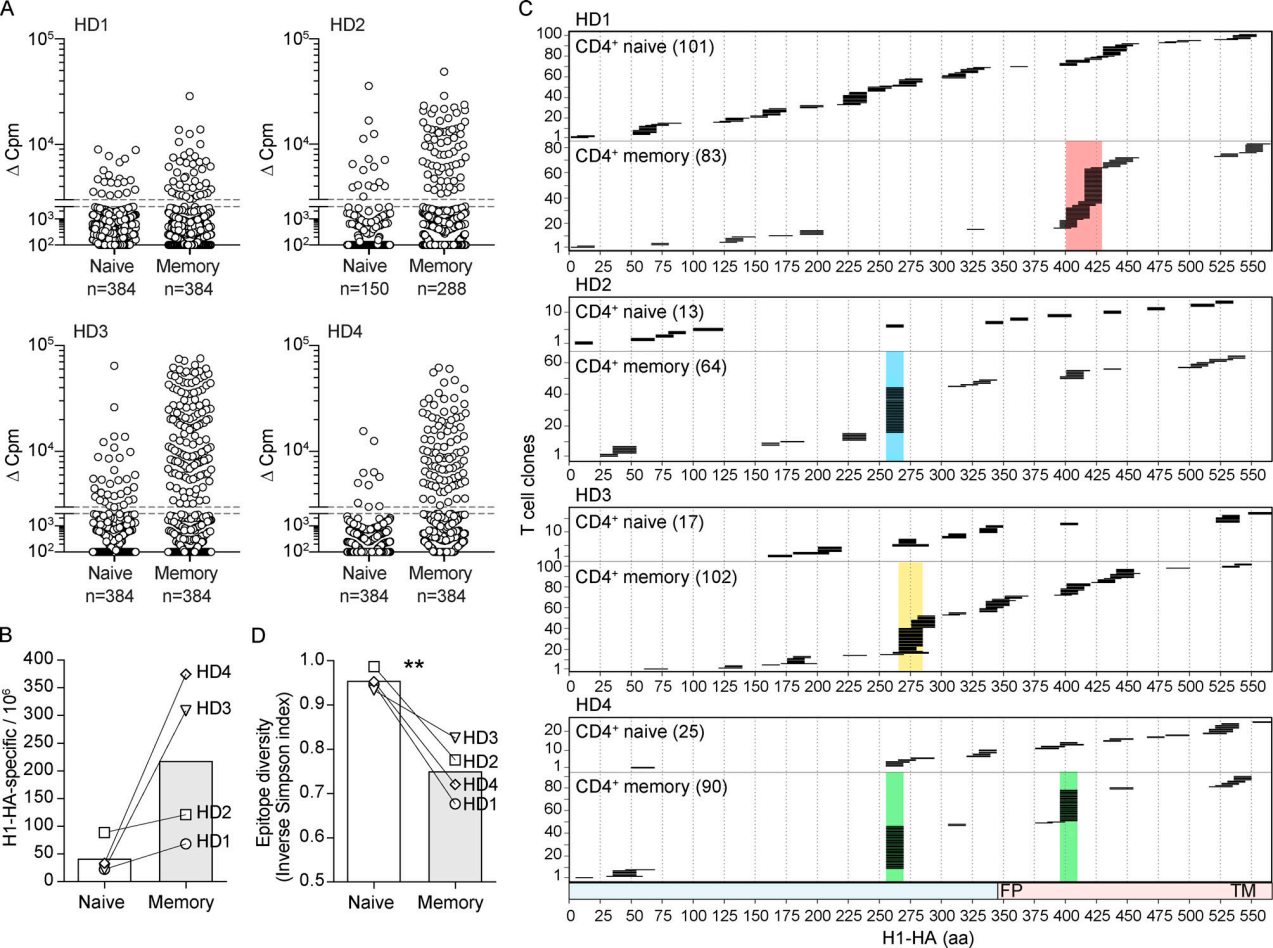
**Isolation and epitope mapping of H1-HA–reactive CD4^+^ T cells from naive and memory compartments of influenza vaccinated donors.** **(A)** Naive and memory CD4^+^ T cells were FACS sorted from PBMCs of four donors and polyclonally expanded in multiple wells, each containing 1,000–2,000 cells. The number of wells ranged from 150 to 384, depending on the number of cells isolated (see Materials and methods). After 14–21 d, the amplified naive and memory T cell libraries were screened against a pool of overlapping peptides spanning the entire H1-HA sequence in the presence of autologous APCs. Proliferation was assessed on day 4 after a 16-h pulse with [^3^H]thymidine. Data are expressed as the counts per minute, after subtraction of background proliferation (Δcpm). The proliferative response of each T cell clone in the library is represented by a single dot. The specificity of positive cultures was confirmed in three independent experiments. **(B)** Frequencies of H1-HA–specific T cells within naive or memory CD4^+^ T cells was calculated based on number of negative wells according to the Poisson distribution. Data are expressed as frequency per million of naive or memory CD4^+^ T cells. Each symbol indicates a different donor. **(C)** Epitope mapping of H1-HA–specific T cell clones from naive or memory libraries. The epitopes were identified by screening the T cell cultures with overlapping peptides spanning the entire H1-HA sequence in at least two independent experiments, with consistent results. The x axis indicates H1-HA amino acid sequence; each segment represents the sequence recognized by individual T cell lines. The numbers of H1-HA–reactive T cell clones in the naive or memory library of each donor is reported. The chart at the bottom indicates HA1 and HA2 domains colored in blue and red, respectively (FP, fusion peptide; TM, transmembrane). The immunodominant regions identified in the memory compartment of each donor are highlighted with color-coded shadows. Immunodominant H1-HA regions and percentage of immunodominant T cell clones were HD1, H1-HA_401–430_, 49%; HD2, H1-HA_256–270_, 45.3%; HD3, H1-HA_266–285_, 24.5%; HD4, H1-HA_256–270_, 42.2%; and H1-HA_396–410_, 31.1%. **(D)** Richness and evenness of the pool of H1-HA–specific T cell clones isolated from naive or memory libraries were evaluated as inverse Simpson index of diversity (1-D), calculated based on the number of T cell clones recognizing each particular H1-HA peptide. Inverse Simpson index (1-D) ranges between 0 and 1, and reflects the probability that two H1-HA–specific T cell clones randomly selected from a repertoire recognize different H1-HA epitopes. **, P = 0.0097 as determined by two-tailed paired *t* test.

We next investigated whether the immunodominance observed in the memory repertoire is a general phenomenon and whether it is reflected in the naive repertoire of a given individual. To address these questions, we used the highly sensitive T cell library method (Geiger et al., 2009) to screen naive and total memory CD4^+^ T cells from HD1 and three other immune donors with a diverse HLA background (Table S3). For each donor, naive and memory T cells were polyclonally expanded in multiple cultures (each containing 1,000–2,000 cells) in the presence of phytohemagglutinin, IL-2, and feeder cells. For a broad and unbiased screening of T cell reactivity against H1-HA, the T cell libraries were then screened using overlapping 15mer peptides covering the entire H1-HA sequence. In all four donors tested, H1-HA peptide–specific T cell clones were readily detected in naive and memory libraries although, as expected, their frequencies measured in the naive libraries were lower compared with that measured in the memory libraries (Fig. 2, A and B). Epitope mapping of memory T cell clones confirmed in all four donors a skew toward one or two immunodominant regions, which for donor HD1 coincided with those detected by antigen-driven proliferation of memory T cell subsets (Fig. 2 C). Strikingly, however, epitope mapping of naive T cell clones showed that these cells covered a broad range of peptide specificities (Fig. 2, C and D). This pattern was particularly evident for donor HD1, which was analyzed at high depth. In this donor, naive T cells with diverse TCR Vβs recognized peptides spanning virtually all the H1-HA sequence (Fig. 2 C and Table S4). Collectively, these findings demonstrate that the naive T cell repertoire has a very broad coverage of the H1-HA sequence and that only a fraction of this repertoire is selected in the memory repertoire.

### Naive and memory T cell clones show different functional avidities for peptide and naturally processed H1-HA

A plausible explanation for the selection of immunodominant peptides is their binding affinity to MHC molecules and/or TCRs. We therefore measured the binding affinity of dominant and subdominant peptides to the recombinant HLA-DR molecules of donor HD1 (alleles DRB1*01:01 or *08:01). In vitro refolding assays performed in the presence of titrated peptides showed that the immunodominant H1-HA_401–420_ and H1-HA_411–430_ peptides bound to HLA-DRB1*08:01 molecules with an affinity that was comparable to that of subdominant peptides binding to the same HLA-DR (Fig. S1 C), indicating that immunodominance of these peptides is not explained by preferential binding to MHC class II molecules.

**Figure S2.**
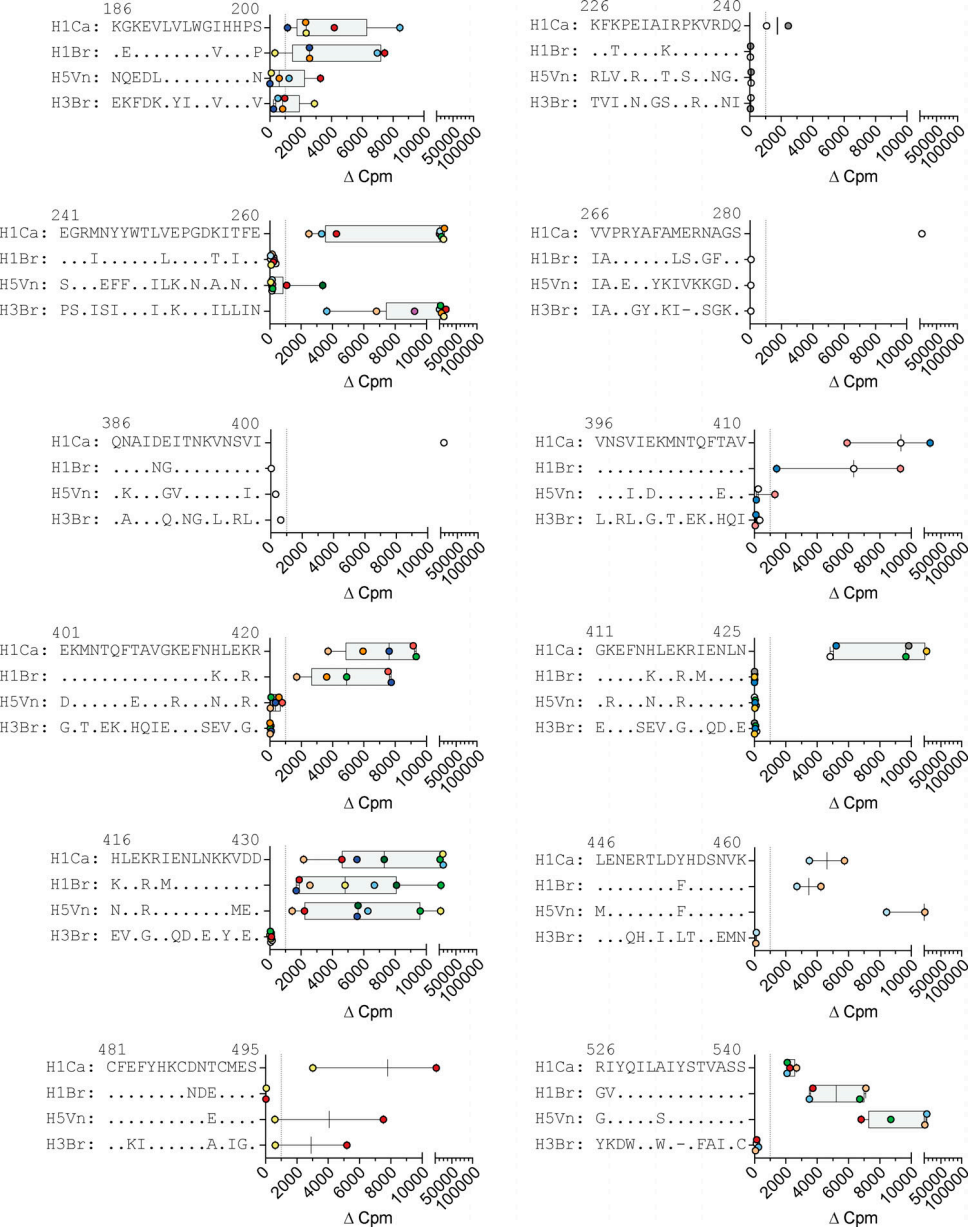
**T cell clones from donor HD1 specific for different H1-HA peptides were tested for their ability to cross-react to HAs from different influenza A strains.** Briefly, T cell clones were stimulated with autologous APCs pulsed with recombinant HAs from A/California/07/2009 (H1N1, H1Ca), A/Brisbane/59/2007 (H1N1, H1Br), A/Viet Nam/1203/2004 (H5N1, H5Vn), or A/Brisbane/10/2007 (H3N2, H3Br). Proliferation was assessed on day 3 after a 16-h pulse with [^3^H]thymidine and expressed as Δcpm. Shown are 45 representative T cell clones, each bearing a unique TCR Vβ sequence. Data are grouped based on the epitope specificity of the T cell clones tested. Homologous peptides from the different influenza A strains are reported as sequence alignment.

Immunodominance to HA antigen may be also the result of repeated exposure through natural infection or vaccination that can select cross-reactive T cells. However, as shown in Fig. S2, T cell clones responding to immunodominant or subdominant H1-HA peptides were both capable to cross-react, to variable but similar extents, to H1-HA and/or H3-HA from the widespread strains A/Brisbane/59/2007 (H1N1) or A/Brisbane/10/2007 (H3N2). A few clones cross-reacted to H5-HA from the highly pathogenic subtype A/Viet Nam/1203/2004 (H5N1).

**Figure 3.**
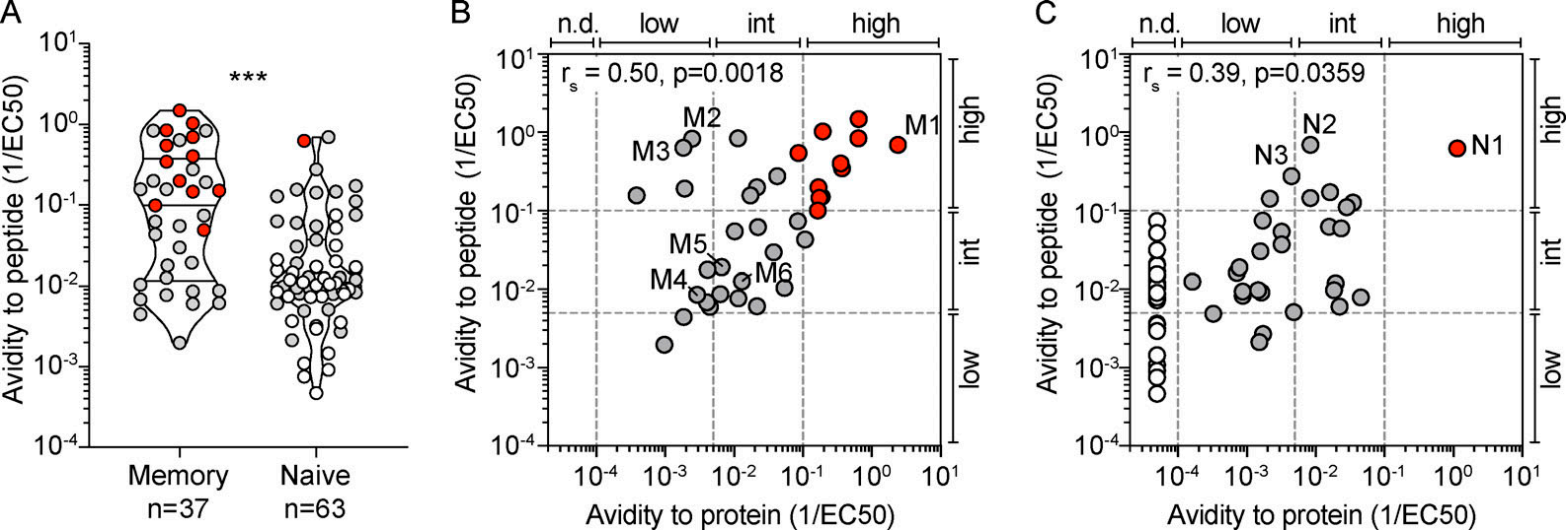
**H1-HA–specific naive and memory CD4^+^ T cell clones show different functional avidities.** Functional avidity of H1-HA–reactive CD4^+^ T cell clones isolated from donor HD1 was determined by stimulation with titrated doses of synthetic H1-HA peptides or recombinant H1-HA in the presence of autologous monocytes. Proliferation was assessed on day 3 after a 16-h pulse with [^3^H]thymidine; EC_50_ values were calculated by nonlinear regression curve fit. Shown are mean data of one experiment done with each clone in duplicate and are representative of three independent experiments. **(A)** Violin plots of the frequency distribution of reciprocal EC_50_ values of T cell clones from the memory or naive compartment stimulated with titrated doses of H1-HA peptides. T cell clones specific for the immunodominant H1-HA_411–430_ epitope are reported as red dots. Lines represent the median and quartiles. ***, P < 0.001 as determined by two-tailed Mann–Whitney *U* test. **(B and C)** Scatter plots of reciprocal EC_50_ values of T cell clones from the memory (B) or naive (C) compartment, stimulated in parallel with recombinant H1-HA (x axis) and synthetic peptides (y axis). EC_50_ values below the detection limit for stimulations with recombinant H1-HA were set arbitrarily to 20 µg/ml; the corresponding T cell clones are reported as white dots. Spearman correlation was calculated based on EC_50_ pairs from T cell clones responding to both peptides and recombinant H1-HA (B, *n* = 36; C, *n* = 29). Thresholds of functional avidity were set arbitrarily at EC_50_ values of 10 µg/ml, 200 ng/ml, and 10 ng/ml of antigen. n.d., not detected.

**Figure S3.**
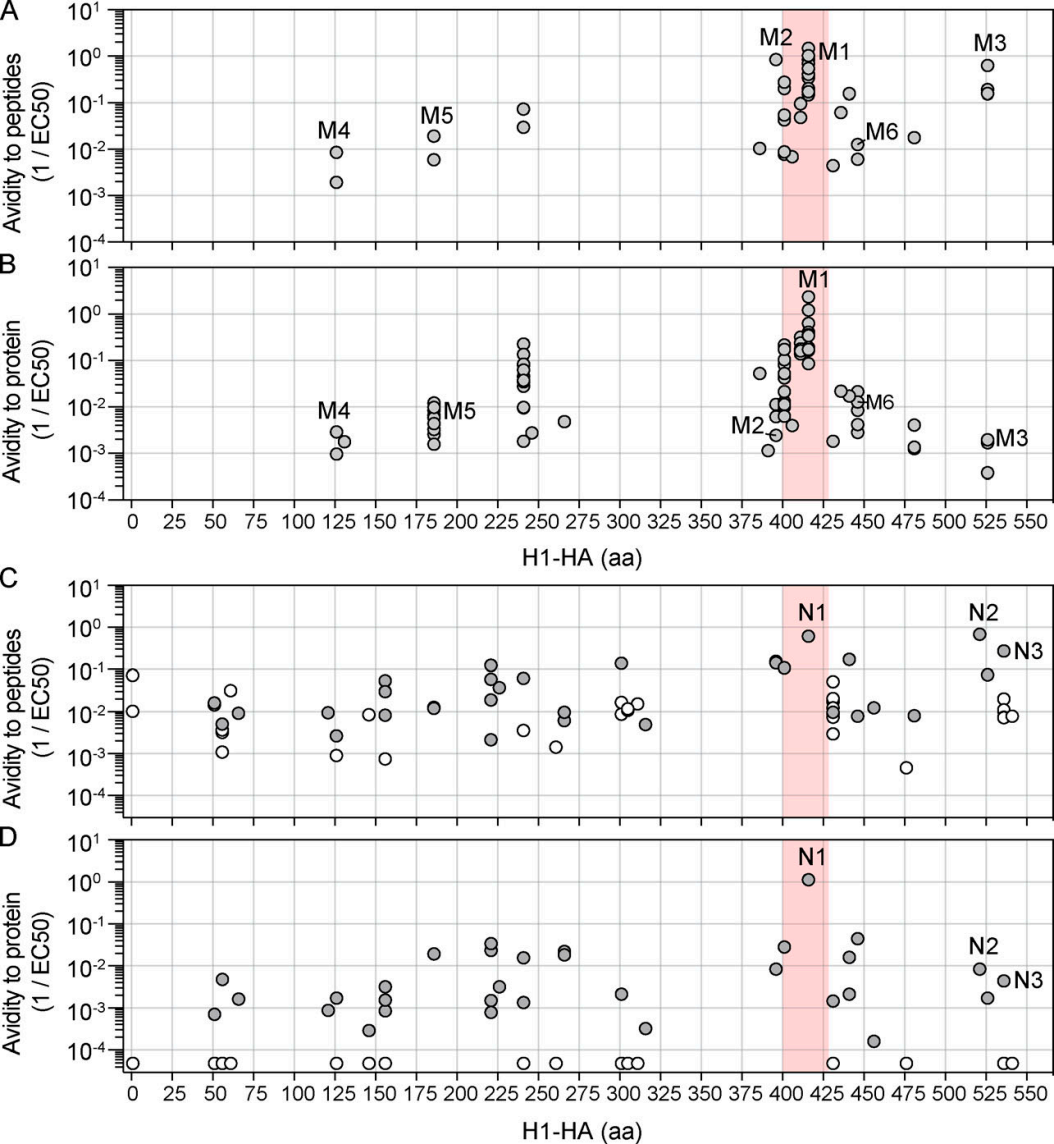
**Functional avidities for peptide and naturally processed H1-HA of T cell clones specific for different epitopes. (A–D) **Functional avidity of H1-HA–reactive T cell clones isolated from the memory (A and B) or the naive (C and D) compartment of donor HD1 was determined by stimulation with titrated doses of synthetic peptides (A, *n* = 37 clones; C, *n* = 63) or recombinant H1-HA (B, *n* = 79; D, *n* = 63). Data are expressed as reciprocal EC_50_ values. Each dot represents an individual T cell clone; the position of the dots on the x axis indicates the starting residue of the cognate peptide. EC_50_ values below the detection limit for stimulations with recombinant H1-HA were set arbitrarily to 20 µg/ml; the corresponding T cell clones are reported as white dots. The immunodominant H1-HA_401–430_ region identified in the memory compartment is highlighted with a red shadow.

We then selected a large number of H1-HA–specific T cell clones derived from naive or memory T cells of donor HD1 and determined their functional avidity by measuring the proliferative response to autologous monocytes pulsed with different concentrations of the H1-HA peptides or H1-HA protein, which need processing for presentation on MHC-II molecules. Functional avidity, measured in response to peptide stimulation and expressed as half-maximal concentration (EC_50_) value, was spread over almost 3 logs for both types of T cell clones, with clones from memory T cells being enriched for high-avidity cells (Fig. 3 A and Fig. S3, A and C), consistent with previous observations on the response to tetanus toxoid (Geiger et al., 2009). When the same clones were tested for their response to H1-HA protein, several observations were made. First, clones from memory T cells responded to H1-HA protein, and there was an overall correlation between EC_50_ values for protein and peptide, with the immunodominant clones, such as M1 specific for H1-HA_411–430_, showing the highest avidity for both peptide and protein (red dots in Fig. 3 B). However, there were a few notable exceptions. For instance, the subdominant clones M2 and M3, specific for H1-HA_396–410_ and H1-HA_526–540_, had high functional avidity for peptide, comparable to clone M1, but showed 1,000-fold lower avidity for the H1-HA protein (Fig. 3 B). Second, memory T cell clones restricted by HLA-DP (M4 specific for H1-HA_121–140_) or by HLA-DQ molecules (M5 and M6 specific for H1-HA_186–200_ and H1-HA_446–460_, respectively) recognized peptides and protein with functional avidity lower than the median of the distribution (Fig. 3, A and B). Third, several clones from naive T cells, including some with intermediate avidity for peptides, did not proliferate in response to H1-HA protein (Fig. 3 C). Finally, and importantly, the differential recognition of dominant and subdominant epitopes in response to H1-HA was also observed for clones derived from naive T cells. For instance, clone N1, specific for the immunodominant H1-HA_411–430_ peptide, had high functional avidity for both peptide and naturally processed H1-HA, comparable to that of memory clone M1, while clones N2 and N3 (specific for the subdominant peptides H1-HA_521–540_ and H1-HA_536–555_, respectively) had high avidity for peptide but 100-fold lower avidity for protein, similar to what was observed for memory clones M2 and M3 (Fig. 3 C).

Collectively, these findings indicate that in both the naive and memory repertoires, immunodominant peptides are recognized with high avidity by T cells. They also reveal the presence of a broad repertoire of naive T cells for cryptic HA peptides. Finally, they show that the functional avidity of peptide recognition is not predictive of the response to the naturally processed antigen, suggesting a major role for antigen processing in determining the abundance of the processed peptide generated.

### The MHC class II peptidome defines immunodominant regions and reveals modulation for antigen processing by antibodies

**Figure 4.**
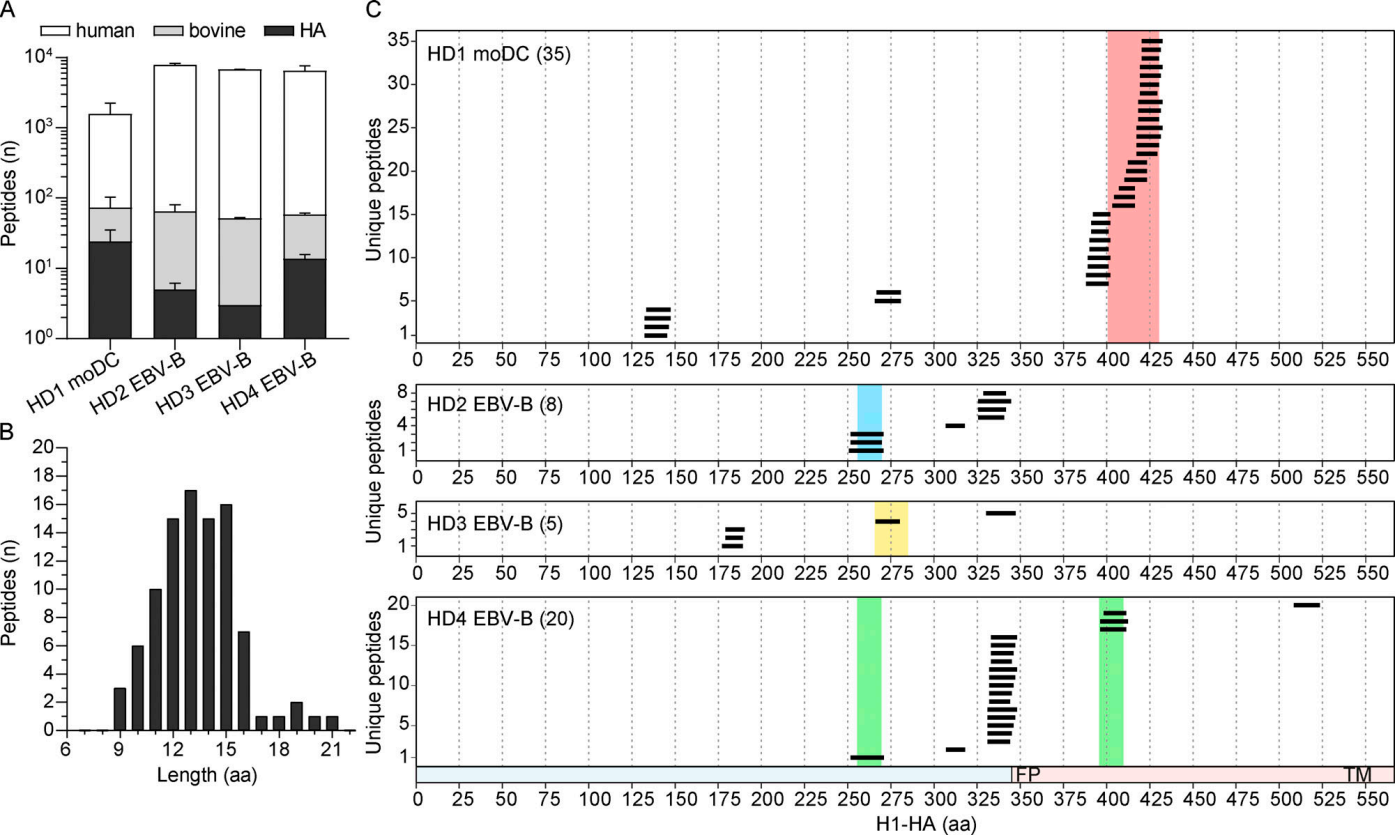
**The MHC class II peptidome defines H1-HA immunodominant regions targeted by memory CD4^+^ T cells.** Monocyte-derived DCs (moDC) from donor HD1 and H1-HA–specific EBV-B cell clones from donors HD2-HD4 were pulsed with recombinant H1-HA, and MHC-II presented peptides were measured by MS-based immunopeptidomics. **(A)** Number of MHC-II eluted peptides measured by MS in the different donors (mean ± SD of *n* = 2 independent experiments for HD1 and HD3, *n* = 3 for HD2, *n* = 4 for HD4). Color code indicates the different source organism of the measured peptides. **(B)** Histogram of the lengths of H1-HA–derived peptides eluted from MHC-II molecules reported in A. **(C)** Sets of H1-HA–derived peptides eluted from MHC-II molecules from each donor. The x axis indicates H1-HA amino acid sequence. Each segment represents a unique H1-HA–derived peptide identified by MS (union of *n* = 2 independent experiments for HD1 and HD3, *n* = 3 for HD2, *n* = 4 for HD4); the total numbers of H1-HA–derived peptides are reported. The chart at the bottom indicates HA1 and HA2 domains colored in blue and red, respectively (FP, fusion peptide; TM, transmembrane). The immunodominant regions targeted by memory CD4^+^ T cells of each donor are reported with color-coded shadows.

Based on the above results, we hypothesize that CD4^+^ T cell immunodominance could be primarily related to the yield of peptides generated by antigen processing by APCs. We therefore used MS-based immunopeptidomics to identify peptides naturally presented on MHC-II molecules by different types of APCs. Briefly, monocyte-derived dendritic cells (DCs) from donor HD1 and EBV-immortalized B cell clones carrying surface BCRs specific for H1-HA from all four donors were pulsed overnight with recombinant H1-HA. MHC-II molecules were isolated from lysed cells using a pan-anti–MHC-II antibody, and peptides were purified by reversed-phase chromatography. Using this approach, we identified thousands of total MHC-II bound individual peptides derived from self-molecules as well as 3–35 H1-HA–derived peptides (median 9) with the expected length peaking at 12–15 aa (Fig. 4, A and B). Remarkably, almost 60% of the H1-HA–derived peptides presented by DCs of donor HD1 corresponded to a rich set of nested peptides overlapping the immunodominant regions H1-HA_401–430_ targeted by memory T cells, suggesting that peptides in this region are presented in high abundance and are recognized by T cells with high functional avidity (Fig. 4 C and Table S5). A similar correspondence between immunodominant peptides recognized by memory T cells and peptides presented on MHC-II molecules was found in the other three donors analyzed (Fig. 4 C and Table S6), although the breadth of the analysis in these donors was limited by the amount of peptides that could be retrieved from B cells. A notable exception, however, was observed for peptide H1-HA_326–340_ that was presented at high abundance by B cells of donor HD4 that lacked memory T cells specific for the same peptide.

**Figure S4.**
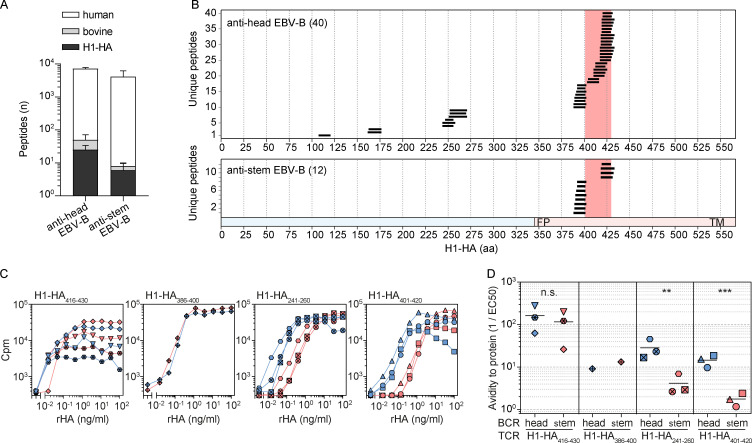
**The MHC-II peptidome reveals modulation for antigen processing by antibodies.** EBV-B cell clones from donor HD1 specific for H1-HA head or H1-HA stem were pulsed with recombinant H1-HA, and MHC-II presented peptides were measured by MS-based immunopeptidomics. **(A)** Number of MHC-II eluted peptides measured by MS in anti-head or anti-stem EBV-B cell clones (mean ± SD of *n* = 3 independent experiments). Color code indicates the different source organism of the measured peptides. **(B)** Sets of H1-HA–derived peptides eluted from MHC-II molecules of anti-head or anti-stem EBV-B cell clones. The x axis indicates H1-HA amino acid sequence. Each segment represents a unique H1-HA–derived peptide identified by MS (union of *n* = 3 independent experiments); the total numbers of H1-HA–derived peptides are reported. The immunodominant H1-HA_401–430_ region targeted by memory CD4^+^ T cells is highlighted with a red shadow. The chart at the bottom indicates HA1 and HA2 domains colored in blue and red, respectively (FP, fusion peptide; TM, transmembrane). **(C and** **D)** The effect of BCR specificity in modulating antigen processing was tested by coculture of H1-HA–specific T and EBV-B cell clones in the presence of titrated antigen. Briefly, functional avidity of memory T cell clones specific for different H1-HA epitopes was determined by stimulation with titrated doses of recombinant H1-HA in the presence of the indicated EBV-B cell clone as APCs. Each individual T cell clone tested was restricted by HLA-DR and carried a different rearranged TCR Vβ sequence. **(C)** Proliferation was assessed on day 3 after a 16-h pulse with [^3^H]thymidine and expressed as counts per minute. Proliferation curves are grouped based on the epitope specificity of the T cell clones, reported in the top-left corner of each plot. Each symbol refers to a distinct T cell clone stimulated in the presence of anti-head (blue) or anti-stem (red) EBV-B cell clone as APCs. **(D)** EC_50_ values were calculated by nonlinear regression curve fit. Each symbol indicates the reciprocal EC_50_ value of T cell clones co-cultured with anti-head (blue) or anti-stem (red) EBV-B cell clone as APCs. Lines represent mean values. Data are grouped based on the epitope specificity of the T cell clones. n.s., not significant; **, P = 0.0041; ***, P = 0.0004, as determined by two-tailed ratio paired *t* test.

The antibody response to influenza A virus is directed against several regions of HA, such as the highly variable globular head or the conserved stem region, that can be the target of neutralizing antibodies with high breadth for multiple viral subtypes (Corti et al., 2011; Lee and Wilson, 2015). The MHC-II peptidome of H1-HA–pulsed B cell clones with distinct epitope specificity isolated from donor HD1 revealed that a clone specific for the HA globular head presented the H1-HA immunodominant region in a similar manner as DCs, whereas a B cell clone specific for the HA stem region was a poorer presenter of H1-HA–derived peptides (Fig. S4, A and B). Indeed, even if we resolved a comparable total number of MHC-II presented peptides in the two kinds of B cells (Fig. S4 A), from the anti-stem clone we did not detect any H1-HA–derived peptides corresponding to the dominant H1-HA_401–420_ region, nor any subdominant peptides from the HA1 domain (Fig. S4 B). Consistently, presentation of the recombinant H1-HA by anti-stem B cells resulted in a much lower activation and proliferation of T cell clones specific for H1-HA_401–420_ or H1-HA_241–260_ (both HLA-DR restricted), resulting in a 10-fold reduction of their functional avidity compared with antigen presentation by anti-head B cells (Fig. S4, C and D). Conversely, HLA-DR–restricted T cell clones specific for H1-HA_416–430_ or H1-HA_386–400_, which were detected by MHC-II immunopeptidomics on both anti-head and anti-stem B cell clones, showed comparable functional avidity when stimulated with either kind of APC (Fig. S4, C and D).

Taken together, these data show that the MHC-II peptidome presented by professional APCs defines the immunodominant regions of HA recognized by memory CD4^+^ T cells. Moreover, the spectrum of peptides naturally presented by B cells can be modulated by antibody binding to HA, thus potentially being an additional variable affecting B cell clonal selection during T cell–dependent immune responses.

### Epitope prediction can be improved by combining MHC binding affinity prediction and antigen processing likelihood (APL)

**Figure S5.**
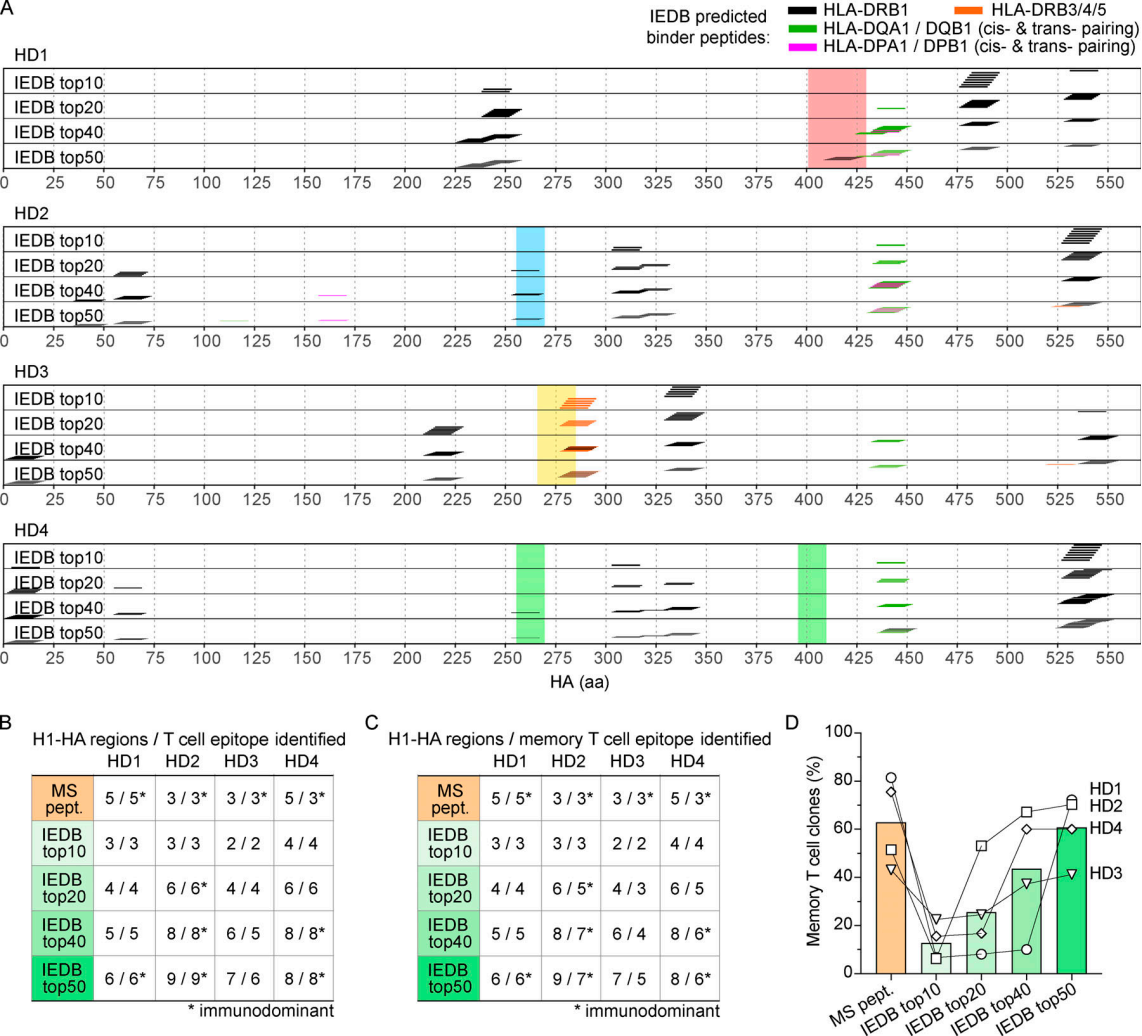
**Peptide binding to MHC-II is a necessary but not sufficient condition to define immunodominance.** MHC-II binding affinity of each theoretical H1-HA 15mer peptide was calculated using the IEDB tool for MHC-II binding prediction (http://tools.iedb.org/mhcii/). Personalized analyses were performed by considering the MHC-II alleles carried by each donor (HLA-DRB1, HLA-DRB3/4/5, HLA-DQA1/DQB1 in cis- or trans-pairing, HLA-DPA1/DPB1 in cis- or trans-pairing). Top scoring H1-HA 15mer peptides for each donor were selected based on percentile rank calculated by comparison to a large set of random natural peptides. **(A)** Sets of H1-HA peptides predicted as MHC-II binders at different thresholds for each donor. The x axis indicates H1-HA amino acid sequence. The sets of top predicted MHC-II binder peptides are reported as color-coded segments. The immunodominant regions targeted by memory CD4^+^ T cells of each donor are reported with color-coded shadows. **(B and C)** IEDB-predicted peptides and MHC-II eluted peptides measured by MS-based peptidomics defined discrete H1-HA regions. The tables summarize the number of HA regions found presented on MHC-II by MS-based peptidomics, or predicted as MHC-II binders in different donors. The corresponding number of HA epitopes recognized by at least one T cell clone regardless of the subset of origin (B) or isolated from the memory compartment (C) are reported. Identification of immunodominant epitopes is marked by an asterisk. **(D)** Sensitivity was evaluated in terms of percentage of memory T cell clones for which the cognate peptide was identified by MS-based MHC-II peptidomics (MS pept., orange bar) or by MHC-II binding predictions (IEDB) at different thresholds (IEDB, green bars). Each symbol represents a different donor.

The binding of processed peptides to the groove of MHC-II molecules follows precise rules that have been instrumental to the development of algorithms capable of predicting binding affinity with high accuracy (Jensen et al., 2018; Unanue et al., 2016; Wang et al., 2010). Using the IEDB tool for MHC-II binding prediction, we found that virtually all the H1-HA 15mer peptides predicted as good binders for the MHC-II alleles carried by the four donors analyzed were recognized by T cell clones isolated from either the naive or the memory compartment (Fig. S5). Nevertheless, it was surprising to note that the immunodominant H1-HA peptides did not show stronger binding to MHC-II compared with subdominant or cryptic peptides, by either in silico prediction or, as demonstrated before, in vitro measurement of MHC-II binding. These data suggest that the binding to MHC-II is a necessary, but not sufficient, feature for T cell immunodominance. We therefore set out to explore further parameters that might improve the in silico prediction of HA-derived T cell epitopes.

**Figure 5.**
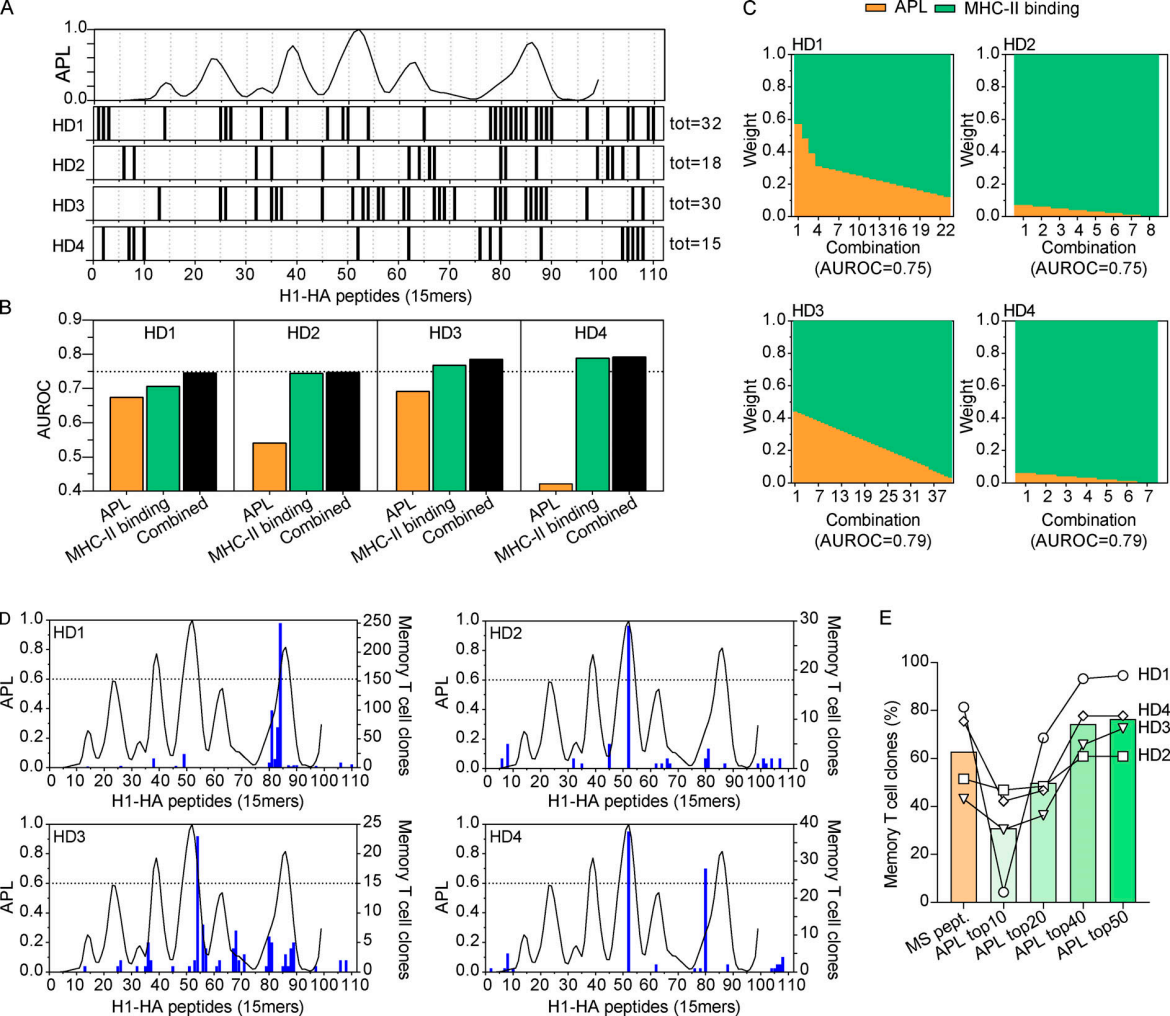
**Immunodominant epitopes localize in H1-HA regions predicted as promptly liberated by endosomal proteases.** **(A)** APL based on structural accessibility was calculated for each theoretical H1-HA 15mer peptide (upper panel). Sets of H1-HA epitopes mapped in the memory CD4^+^ T cell repertoire of each donor (lower panels); each segment indicates a peptide recognized by at least one T cell clone isolated from memory CD4^+^ T cells. Note that APL could not be calculated in the N-terminal HA region, corresponding to HA2 transmembrane region, since this is not included in the H1-HA crystal structures. **(B)** Performance of in silico predictors was benchmarked by computing ROC curves using the sets of H1-HA memory epitopes reported in A as true positives; the performance of each method was assessed using the AUROC metric. Shown are the maximal AUROC values achieved by single predictors based uniquely on APL (orange bars) or MHC-II binding (green bars) and the optimized combined predictor (black bars). **(C)** Contribution of APL (orange) and MHC-II binding (green) in the different combinations all resulting in the highest AUROC values for each donor (HD1 and HD2, 0.75 ± 0.05; HD3 and HD4, 0.79 ± 0.05). The relative contribution of APL and MHC binding would differ across donors, since each one has a distinct HLA background and a distinct set of peptides recognized by memory T cells. **(D)** APL (black curve) and peptide specificity (blues bars) of memory CD4^+^ T cell clones of each donor. **(E)** Percentage of memory T cell clones for which the cognate peptide was identified by MS-based MHC-II peptidomics (MS pept., orange bar) or by APL at different thresholds (green bars). Each symbol represents a different donor.

The molecular context in which a peptide is embedded and its structural accessibility might influence the propensity of unfolding during the progressive pH acidification that occurs in the endocytic pathway, therefore affecting the exposition of denatured stretches of the antigen to the proteolytic environment of the late endosomes (Graham et al., 2018; Kim and Sadegh-Nasseri, 2015; Landry, 2008). To evaluate the role of structural constraints of HA in influencing the immunodominance observed in the memory repertoires, we adopted a recently developed algorithm that uses antigen conformational stability to estimate the likelihood of antigen processing (Mettu et al., 2016). In brief, an aggregate z-score of conformational stability was determined for each H1-HA residue by integrating four structural parameters obtained from the 3D structure of postfusion HA resolved by x-ray diffraction (PDB codes: 3LZG for HA1 domain [Xu et al., 2010]; 1HTM for HA2 domain in the postfusion conformation [Bullough et al., 1994]). The z-score statistic was then used to calculate an APL for each theoretical H1-HA 15mer peptide (Fig. 5 A), following the rationale that the liberation of antigenic peptides might be facilitated by surrounding unstable regions that are readily unfolded and targeted by endosomal proteases. As shown in Fig. 5 A, several peptides recognized by memory T cells in donors HD1–HD4 were found in regions of high APL. We then evaluated the performance of the APL and MHC binding predictive algorithms by computing the receiver operating characteristic (ROC) curves using the set of epitopes recognized by memory T cells of each donor as true positives; the performance was assessed using the area under the ROC curve (AUROC) metric (Fig. 5 B). We also built a combined predictor by iteratively weighting the contributions of APL and MHC binding until we could maximize the AUROC value of the predictor. With this approach, we found that in all donors the combined predictor achieved a better AUROC value, outperforming the predictors based solely on APL or MHC binding (Fig. 5 B). As shown in Fig. 5 C, there are a number of ways to combine APL and MHC binding weights to achieve the highest AUROC (0.75 in HD1 and HD2, 0.79 in HD3 and HD4), giving an estimate of the maximum and minimum weight of the two scores for each donor. Importantly, all the immunodominant peptides identified in the memory repertoire of the four donors analyzed were grouped within a peak of high APL (as defined by a threshold of 0.6; Fig. 5 D). On average, the 20 top-scoring peptides predicted by APL accounted for >50% of the HA-specific memory T cell clones, with a sensitivity comparable to the set of peptides measured by MHC-II immunopeptidomics (Fig. 5 E). Thus, a combination of APL and MHC-II peptide binding affinity may be helpful to improve prediction of immunogenicity and immunodominance in CD4^+^ T cell responses.

## Discussion

In this study, we report the identification of influenza H1-HA peptides recognized by naive and memory CD4^+^ T cells in individuals with different HLA haplotypes. Whereas naive T cells recognized a variety of peptides spanning the whole H1-HA sequence, memory T cells were highly focused on just a few peptides. These immunodominant peptides were readily identified by MS-based analysis of peptides eluted from MHC class II molecules isolated from DCs or H1-HA–specific B cell clones and could be better predicted taking into account the HA structural accessibility to proteolytic cleavage. Collectively, these findings indicate that processing of native proteins represents a major constraint determining the immunodominance to influenza HA and delineate new methods to identify immunodominant and cryptic T cell epitopes.

The identification and characterization of antigen-specific T cells in the naive and memory repertoire is of both fundamental and practical relevance. The high-throughput T cell library method used in this study can rapidly identify, in different individuals, the range of peptides that can be recognized by T cells, thus determining, with a simple assay, both peptide binding to MHC-II and the presence of specific TCRs (Campion et al., 2014; Geiger et al., 2009; Latorre et al., 2018). Considering the small size of the naive T cell libraries analyzed (7 × 10^5^ T cells) compared with the total naive T cell pool, we can estimate that the naive repertoire contains a large number of diverse HA-specific T cells, with frequencies ranging from 10^−5^ to 10^−6^ for each epitope and with a range of functional avidities. Importantly, we showed that, in humans, naive T cells recognize multiple peptides spanning the whole H1-HA sequence. The diversity of epitope recognition observed in the naive compartment indicates that a multiplicity of HA-derived peptides can potentially trigger T cell activation, underlining the redundancy of the MHC-II system in accommodating and presenting a large variety of different peptides. Nevertheless, many T cell clones isolated from the naive compartment recognized peptides but not naturally processed protein. This finding suggests that the naive repertoire retains T cell precursors recognizing peptides that fail to be generated and/or presented by professional APCs or that are produced in amounts insufficient to trigger priming of cognate naive T cells. It remains to be established whether these cryptic peptides can be generated through unconventional antigen processing, as suggested in some cases of tissue antigens (Mohan and Unanue, 2012; Sadegh-Nasseri and Kim, 2015), or by nonprofessional APCs, such as epithelial cells in the respiratory tract that are main targets of influenza viruses. Epithelial cells readily up-regulate MHC-II molecules in response to inflammatory cytokines or viral infection (Gao et al., 1999; Reith and Mach, 2001) and, although they have limited endocytic potential, they can generate peptide ligands for MHC-II presentation through endogenous degradation pathways, such as autophagy and macroautophagy (Dengjel et al., 2005; Schmid et al., 2007).

The analysis performed on memory T cell libraries and on ex vivo–stimulated memory T cells revealed the presence, in each individual, of one or two immunodominant sites targeted by polyclonal and clonally expanded T cells as well as a few subdominant sites. Based on the findings from the analysis of the naive repertoire, this immunodominance cannot be explained by holes in the repertoire. Importantly, our study shows that the immunodominant peptides correspond to those that are found most abundantly in the MHC-II peptidome from H1-HA–pulsed DCs and identified also on H1-HA–specific B cells, although in the latter case, the assay had a limited sensitivity. These findings point to a simple model whereby immunodominance is determined by the abundance of a given peptide–MHC complex generated by processing followed by selection and clonal expansion of high avidity T cells.

The functional avidity of memory T cell clones was on average 10-fold higher compared with that of naive T cell clones, consistent with the notion that high-avidity T cells are selected in the memory pool, as originally reported for mouse CD8^+^ T cells (Busch and Pamer, 1999; McHeyzer-Williams et al., 1999; Savage et al., 1999). The memory repertoire contains also subdominant memory T cell clones that showed very high functional avidity when stimulated by peptides, but not by naturally processed H1-HA protein, suggesting that subdominance may be simply due to a lower abundance of the naturally processed peptide. Collectively, our data reveal that only a small fraction of the peptides that bind to MHC-II molecules and can be recognized by T cells are generated by antigen processing, and even in this case, the yield of processed peptide can vary ≥100-fold between immunodominant and subdominant peptides.

Previous studies using tetanus toxoid as a model antigen showed that antibodies can modulate antigen processing by enhancing or suppressing the generation of different T cell epitopes (Simitsek et al., 1995; Watts and Lanzavecchia, 1993). These findings are extended by the analysis of donor HD1, where a B cell clone with a BCR specific for the H1-HA globular head generates the same sets of immunodominant peptides as DCs, while a B cell clone specific for the H1-HA stem generates a different set of peptides, as demonstrated by peptidomics and activation of specific T cell clones. At this stage, we do not have a mechanistic explanation for these findings, since the structural characterization of the anti-stem antibody and anti-head antibody in complex with HA is not available, and the antigen used was an uncleaved HA0 that is not fusion competent. We can only speculate that by stabilizing a protein domain or by locking HA in the prefusion conformation, certain antibodies might change processing of HA by endosomal cathepsins, leading to decreased production of relevant T cell peptides (Corti et al., 2011; Lee and Wilson, 2015). Further experiments using native antigens and well-characterized antibodies or B cells will be necessary to address the impact of anti-head and anti-stem antibodies in HA processing and presentation to T cells and its physiological relevance in the context of the response to influenza virus infection or vaccination.

The high-resolution epitope mapping of naive and memory T cells from donors with a diverse MHC background offered us the possibility to benchmark currently available in silico predictors of CD4^+^ T cell immunogenicity. Indeed, we found that, although being a prerequisite for recognition by T cells, peptide–MHC-II binding affinity, either predicted in silico or measured in vitro, is a weak correlate of T cell recognition and in particular of immunodominance. Along with the protein antigen expression level and subcellular localization, the position within the 3D structure of the native antigen may profoundly influence the amount of processed peptides (Abelin et al., 2019; Graham et al., 2018). Recent reports have shown that T cell epitopes from viral antigens tend to localize adjacent to highly flexible, surface-exposed regions of the protein that could act as sites of initial proteolytic cleavage (Koblischke et al., 2017; Landry, 2008; Mirano-Bascos et al., 2008), suggesting that the physical accessibility within the tertiary structure is a requirement for efficient peptide release by proteases. By analyzing the 3D conformation of HA, we found that immunodominant epitopes are embedded in regions predicted as readily accessible targets of endosomal proteases. Furthermore, combined in silico analysis of both peptide–MHC-II binding affinity and APL yielded higher predictive values for the set of HA memory epitopes here described, thus indicating a possible strategy to develop more accurate predictive algorithms for T cell immunogenicity of protein antigens.

In conclusion, the findings here reported suggest a model for epitope selection by antigen processing based on a trade-off between multiple factors, including the structural accessibility to proteases and the binding affinity of liberated peptides for the groove of MHC-II molecules. Structural constrains might define regions prone to be liberated at a higher rate by protease cleavage, thus being a property intrinsic of the antigen tridimensional structure, whereas the MHC-II allelic background of each individual would select the final sequences of high-affinity binder peptides. The implications of this model are that highly accessible epitopes could be presented at high abundance on the APC surface even if they are not strong MHC-II binders, and conversely, potentially strong binder peptides could never be presented at relevant amounts if they are not accessible to proteolytic liberation or if they are destroyed by cathepsin cut (resulting in crypticity). The net result of such complex processes would be the differential abundance of some MHC-II presented peptides, which might drive more prominent clonal expansion of cognate naive T cells, leading to immunodominance. Perturbation of the antigen structure, for instance by bound immunoglobulins, might further alter the substrate for endosomal proteolysis and therefore influence the antigen presentation and interaction with cognate T cells.

## Materials and methods

### Cell purification and sorting

Serial blood samples from healthy donor 1 (HD1) vaccinated with Inflexal V 2013/14 (Crucell) and from blood donors from the Swiss Red Cross were used in compliance with the Federal Office of Public Health (authorization no. A000197/2 to F. Sallusto) and approval from the Ethical Committee of Canton Ticino (authorization no. 2018-02166/CE 3428). Blood from HD2, HD3, and HD4 vaccinated with Fluarix Tetra 2015/16 (GlaxoSmithKline) was obtained from the Surrey Clinical Research Centre (University of Surrey, UK). These studies were conducted in compliance with relevant local guidelines, approved by the London-Surrey Borders Research Ethics Committee (reference 15/LO/1649) and registered on ClinicalTrials.gov (reference NCT02557802). Written informed consent was obtained from all subjects participating in the study. Peripheral blood mononuclear cells (PBMCs) were isolated with Ficoll-Paque Plus (GE Healthcare). Monocytes were isolated from PBMCs by positive selection using CD14 magnetic microbeads (Miltenyi Biotech). CD14-depleted fractions were stained at 37°C for 15 min with a primary anti-human CXCR5 (clone 51505; MAB190) from Bio-Techne, followed by staining with a biotinylated secondary goat anti-mouse IgG_2B_ (1090-08) from Southern Biotech. After washing, cells were stained on ice for 30 min with PE/Cy7-Streptavidin (405206) from BioLegend, and with the following fluorochrome-labeled mouse monoclonal antibodies: CD8-PE–Cy5 (clone B9.11; A07758), CD25-PE–Cy5 (clone B1.49.9; IM2646) from Beckman Coulter, CD22-FITC (clone HIB22; 555424) from BD Biosciences, CD4–PE–Texas Red (clone S3.5; MHCD0417), CD45RA-Qdot 655 (clone MEM-56; Q10069), CD95-PerCP-eFluor 710 (clone DX2; 46-0959-42) from Thermo Fisher Scientific, CCR7–BV421 (clone G043H7; 353208) from BioLegend, and Alexa Fluor 647–conjugated goat anti-human IgG (109-606-170) from Jackson ImmunoResearch. Naive and memory CD4^+^ T cells were sorted to >98% purity on a FACSAria III (BD) after exclusion of CD8^+^, CD22^+^, and CD25^bright^ cells. Naive T cells were sorted as CD4^+^CD45RA^+^CCR7^+^CD95^−^; the remaining CD4^+^ T cells were sorted as total memory cells. In some experiments with donor HD1, total memory CD4^+^ T cells were divided in cTfh (sorted as CXCR5^+^ cells), Tcm (sorted as CCR7^+^CXCR5^−^ cells), and Tem (sorted as CCR7^−^CXCR5^−^ cells). IgG^+^ memory B cells and IgG^−^ B cells were sorted to >98% purity after gating on CD22^+^CD4^−^CD8^−^CD25^−^ cells.

### Antigens and peptides

Peptides were synthesized as crude material on a small scale (1 mg) by A&A Labs (San Diego, CA). Peptides used in the study included 15mers overlapping of 10 (112 peptides) or 20mers overlapping of 10 (56 peptides) covering the entire sequence of A/California/07/2009 (H1N1) HA (H1-HA). The numbering of HA T cell epitopes reported in the text refers to the 566-aa-long sequence of A/California/07/2009 HA (UniProtKB: A0A075EXW1). Recombinant HAs from A/California/07/2009 (H1N1), A/Brisbane/59/2007 (H1N1), A/Viet Nam/1203/2004 (H5N1), and A/Brisbane/10/2007 (H3N2) were purchased from Protein Sciences Corp.

### Cell culture

T cells were cultured in RPMI 1640 supplemented with 2 mM glutamine, 1% (vol/vol) nonessential amino acids, 1% (vol/vol) sodium pyruvate, penicillin (50 U/ml), streptomycin (50 µg/ml; all from Invitrogen), and 5% human serum (Swiss Red Cross). For some experiments, medium was supplemented with IL-2 (500 IU/ml). B cells were cultured in RPMI 1640 supplemented with 2 mM glutamine, 1% (vol/vol) nonessential amino acids, 1% (vol/vol) sodium pyruvate, penicillin (50 U/ml), streptomycin (50 µg/ml; all from Invitrogen), and 10% FBS (HyClone, characterized, GE Healthcare Life Science). Sorted IgG^+^ memory B cells were immortalized with EBV and plated in single-cell cultures in the presence of CpG-DNA (2.5 µg/ml) and irradiated PBMC-feeder cells, as previously described (Traggiai et al., 2004). 2 wk after immortalization, the culture supernatants were screened by high-throughput ELISA for binding to H1-HA or H5-HA as described (Pappas et al., 2014). EBV-immortalized B cell (EBV-B) cell clones that resulted positive for binding to H1-HA and/or H5-HA were isolated and expanded. IgG^−^ B cells to be used as APCs for T cell libraries were expanded with CD40L according to an established protocol (Zand et al., 2005). Autologous monocyte-derived DCs were generated by culture in complete medium containing 10% FBS (HyClone) supplemented with recombinant GM-CSF (Gentaur) and IL-4 (ImmunoTools), as previously described (Sallusto and Lanzavecchia, 1994).

### T cell library

Sorted naive or memory CD4^+^ T cells were polyclonally stimulated with 1 µg/ml phytohemagglutinin (Remel) in the presence of irradiated (45 Gy) allogeneic feeder cells (5 × 10^4^ per well) and IL-2 (500 IU/ml) in a 96-well plate. The size of the library (number of wells and initial input of cells seeded per well) depended on the number of cells isolated from each donor. T cell lines were expanded as previously described (Geiger et al., 2009). Library screening was performed 14–21 d after initial stimulation, by culturing thoroughly washed T cells (2.5 × 10^5^ per well) with autologous irradiated B cells (2.5 × 10^4^ per well), untreated or pulsed with a pool of HA overlapping peptides (2 µM per peptide) composed of 15mers (112 peptides, 15mers overlapping of 10) and 20mers (56 peptides, 20mers overlapping of 10) covering the H1-HA sequence. Proliferation was assessed on day 4, after incubation for 16 h with 1 µCi/ml [methyl-^3^H]thymidine (PerkinElmer). Data were expressed as counts per minute. Stringent criteria were used to score positive T cell lines based on two cutoff values: (1) a Δcpm value ≥3 × 10^3^ (cpm with antigen and APCs − cpm with APCs only) and (2) a stimulation index ≥3 (cpm with antigen and APCs ÷ cpm with APCs only). This threshold was chosen based on previous observations made across multiple negative and positive samples assessed by the T cell library technique and with a variety of donors and antigens (Campion et al., 2014; Geiger et al., 2009; Latorre et al., 2018; Lindestam Arlehamn et al., 2013). The specificity of positive cultures was confirmed in subsequent independent experiments of epitope mapping. Precursor frequencies were calculated based on numbers of negative wells, assuming a Poisson distribution (Geiger et al., 2009), and are expressed per million cells within each subset. Inverse Simpson index of diversity (1-D) was calculated for each donor by considering the number of individual T cell clones recognizing each particular H1-HA peptide (Simpson, 1949). The inverse Simpson index (1-D) quantifies the richness and evenness of populations, ranges between 0 and 1, and can be interpreted as the probability that two H1-HA–specific T cell clones randomly selected from a repertoire will recognize different epitopes.

### Isolation of H1-HA–specific T cell clones

Sorted memory CD4^+^ T cell subsets from donor HD1 were labeled with CFSE and cultured at a ratio of 2:1 with irradiated autologous monocytes untreated or pulsed with Inflexal V 2013/2014 (3 µg/ml). After 6 d, cells were stained with antibodies to CD25-PE (clone M-A251; 555432) from BD Biosciences and ICOS-APC (clone C398.4A; 313510) from BioLegend. Proliferating activated T cells were FACS-sorted as CFSE^lo^CD25^+^ICOS^+^ and expanded in vitro in the presence of IL-2 (500 IU/ml). To select HA-specific T cells, Inflexal V–reactive CFSE^lo^ cultures were relabeled with CFSE and stimulated with irradiated autologous monocytes untreated or pulsed with recombinant H1-HA (5 µg/ml). After 5 d, proliferating activated T cells were sorted as CFSE^lo^CD25^+^ICOS^+^ and cloned by limiting dilution. In some experiments, positive cultures from T cell libraries were labeled with CFSE and cultured at a ratio of 2:1 with irradiated autologous monocytes untreated or pulsed with H1-HA peptide pool (2 µM per peptide). After 5 d, proliferating activated T cells were sorted as CFSE^lo^CD25^+^ICOS^+^ and cloned by limiting dilution. T cell clone reactivity was determined by stimulation with H1-HA peptide pool (2 µM per peptide) or recombinant H1-HA (5 µg/ml) in the presence of irradiated autologous monocytes or B cells as APCs. In some experiments, H1-HA peptides or recombinant H1-HA were titrated by serial dilution. Epitope mapping was performed by stimulation of T cell clones with irradiated autologous EBV-B clones, untreated or prepulsed for 2–3 h with individual peptides (15mers overlapping of 10 or 20mers overlapping of 10) covering the entire sequence of H1-HA (2 µM per peptide). To determine MHC restriction of T cell clones, autologous APCs were pulsed for 4 h with recombinant H1-HA, washed extensively, and then cultured with T cells in the absence or presence of blocking anti–MHC-II monoclonal antibodies produced in-house from hybridoma cell lines (anti–HLA-DR, clone L243 from ATCC, HB-55; anti–HLA-DQ, clone SPVL3 [Spits et al., 1983]; anti–HLA-DP, clone B7/21 [Watson et al., 1983]). In all experiments, proliferation was assessed on day 3, after incubation for 16 h with 1 µCi/ml [methyl-^3^H]thymidine (PerkinElmer). Data were expressed as counts per minute.

### TCR Vβ deep sequencing

Ex vivo–sorted memory CD4^+^ T cell subsets and CFSE^lo^ fractions of Inflexal V–stimulated memory CD4^+^ T cell subsets from donor HD1 were analyzed by deep sequencing. In brief, 2.5–5 × 10^5^ T cells were centrifuged and washed in PBS, and genomic DNA was extracted from the pellet using QIAamp DNA Micro Kit (Qiagen), according to manufacturer’s instructions. Genomic DNA quantity and purity were assessed through spectrophotometric analysis. Sequencing of TCR Vβ CDR3 was performed by Adaptive Biotechnologies using the ImmunoSEQ platform. In brief, after a multiplex PCR reaction designed to target any CDR3 Vβ fragments, amplicons were sequenced using the Illumina HiSeq platform. Raw data consisting of all retrieved sequences of 87 nucleotides or corresponding amino acid sequences and containing the CDR3 region were exported and further processed. The assay was performed at deep level for ex vivo–sorted total memory CD4^+^ cells (detection sensitivity, 1 cell in 200,000) and at survey level for CFSE^lo^ Inflexal V–reactive cultures (detection sensitivity, 1 cell in 40,000). Each clonotype was defined as a unique productively rearranged TCR Vβ nucleotide sequence; data processing was done using the productive frequency of reads provided by ImmunoSEQ Analyzer v3.0.

### Sequence analysis of TCR Vβ genes

Sequence analysis of rearranged TCR Vβ genes of HA-specific T cell clones from donor HD1 was performed as previously described (Latorre et al., 2018). Briefly, cDNA from individual T cell clones was obtained by reverse transcription of total RNA from 10^3^–10^4^ cells per reaction. Rearranged TCR Vβ genes were PCR amplified using a forward primer pool targeting Vβ genes and reverse primer pairing to C1–C2 β-chain constant region. Sequence amplification was assessed through agarose gel electrophoresis; successfully amplified fragments were sequenced by Sanger method, and TCR sequence annotation was performed by using IMGT/V-QUEST algorithm (Lefranc et al., 2009).

### HLA typing and peptide–MHC-II binding affinity measurement

HLA genotype of the patients was determined by reverse sequence–specific oligonucleotide probes DNA typing (LABType; One Lambda) performed at the IRCCS San Matteo Hospital Foundation (Pavia, Italy). Affinity measurements of H1-HA 15mer peptides recognized by HLA-DR–restricted T cell clones from donor HD1 to recombinant HLA-DRB1*01:01 or HLA-DRB1*08:01 molecules was performed by Immunitrack (Copenhagen, Denmark), as previously described (Justesen et al., 2009). Briefly, recombinant HLA-DRB1 isoforms were refolded in vitro in the presence of recombinant HLA-DRA and increasing concentrations of H1-HA 15mer peptides. Titrated pan-HLA-DR–binding epitope was used as positive control. After 24-h incubation at room temperature and pH 7, correctly folded heterotrimeric pMHC-II complexes were detected by ELISA; data were analyzed using GraphPad Prism 8 software.

### Purification of MHC-II presented peptides

DCs generated from donor HD1 were pulsed for 2 h with 10 µg/ml recombinant H1-HA at a cellular density of 3 × 10^6^ cells/ml and matured overnight with 100 ng/ml LPS (Enzo Life Sciences) at a cellular density of 1 × 10^6^ cells/ml. HA-specific EBV-B cell clones isolated from IgG^+^ memory B cells of each of the four donors were pulsed overnight with 200 ng/ml recombinant H1-HA at a cellular density of 5 × 10^6^ cells/ml. MHC-II complexes were purified from ∼3 × 10^7^ HA-pulsed DCs or 10^9^ HA-pulsed EBV-B cells with a protocol adapted from Bassani-Sternberg et al. (2015). Briefly, the B cells were lysed with 0.25% sodium deoxycholate, 1% octyl-β-d-glucopyranoside (Sigma-Aldrich), 0.2 mM iodoacetamide, 1 mM EDTA, and Complete Protease Inhibitor Cocktail (Roche) in PBS at 4°C for 1 h. The lysates were cleared by 20-min centrifugation at 18,000 *g* at 4°C, and MHC-II complexes were purified by immunoaffinity chromatography with the anti–HLA-DR/DP/DQ HB-145 monoclonal antibody produced in-house from hybridoma cell line IVA12 (ATCC, HB-145) and covalently bound to protein A Sepharose beads (Thermo Fisher Scientific). The cleared lysates were loaded three times into the affinity columns at 4°C and subsequently washed at 4°C with 10-column volumes of 150 mM NaCl, 20 mM Tris•HCl, pH 8.0 (buffer A); 10-column volumes of 400 mM NaCl, 20 mM Tris•HCl, pH 8; 10-column volumes of buffer A; and finally 10-column volumes of 20 mM Tris•HCl, pH 8. The MHC-II complexes were eluted at room temperature by addition of 500 µl of 0.1 M acetic acid, in total five elutions for each sample. Small aliquots of each eluted fraction were analyzed by 12% SDS-PAGE to evaluate yield and purity of MHC-II complexes. Sep-Pak tC18 (Waters) cartridges were used for further separation of peptides from MHC-II subunits. The cartridges were prewashed with 80% acetonitrile (AcN) in 0.5% formic acid, followed by 0.2% TFA, and subsequently loaded three times with each fraction eluted from the immunoaffinity column. After loading, the cartridges were washed with 0.2% TFA, and the peptides were separated from the more hydrophobic MHC-II chains by elution with 30% AcN in 0.2% TFA. The peptides were further purified using a Silica C18 column tip (Harvard Apparatus) and eluted again with 30% AcN in 0.2% TFA. Finally, the peptides were concentrated by vacuum centrifugation and resuspended in 2% AcN, 0.1% TFA, and 0.5% formic acid for MS analysis.

### Liquid chromatography-tandem MS and data analysis

MHC-II peptides were separated on an EASY-nLC 1200 HPLC system coupled online to a Q Exactive mass HF spectrometer via a nanoelectrospray source (Thermo Fisher Scientific). Peptides were loaded in buffer A (0.1% formic acid) on in-house packed columns (75-µm inner diameter, 50-cm length, and 1.9-µm C18 particles from Dr. Maisch) and eluted with a nonlinear 120-min gradient of 5–60% buffer B (80% AcN and 0.1% formic acid) at a flow rate of 250 nl/min and a column temperature of 50°C. The Q Exactive was operated in data-dependent mode with a survey scan range of 300–1,650 m/z and a resolution of 60,000 at m/z 200. Up to 10 most abundant isotope patterns with a charge ≥1 were isolated with a 1.8-Th-wide isolation window and subjected to higher-energy C-trap dissociation fragmentation at a normalized collision energy of 27. Fragmentation spectra were acquired with a resolution of 15,000 at m/z 200. Dynamic exclusion of sequenced peptides was set to 30 s to reduce the number of repeated sequences. Thresholds for the ion injection time and ion target values were set to 80 ms and 3E6, respectively, for the survey scans and 120 ms and 1E5 for the tandem MS scans. Data were acquired using Xcalibur software (Thermo Fisher Scientific). MaxQuant software was used to analyze MS raw files. Tandem MS spectra were searched against the A/California/07/2009 (H1N1) HA sequence (UniProtKB: A0A075EXW1), the bovine Uniprot FASTA database, the human Uniprot FASTA database, and a common contaminants database (247 entries) by the Andromeda search engine (Cox et al., 2011). N-terminal acetylation and methionine oxidation were set as variable modifications; no fixed modifications were selected; the enzyme specificity was set to unspecific, with a minimum peptide length of 8 aa. A false discovery rate of 1% was required for peptides. Peptide identification was performed with an allowed precursor mass deviation of ≤4.5 ppm and an allowed fragment mass deviation of 20 ppm; “match between runs” option was disabled. The MS proteomics data have been deposited to the ProteomeXchange Consortium via the PRIDE (Perez-Riverol et al., 2019) partner repository with the dataset identifier PXD018151.

### In silico analysis

MHC-II binding affinity of each theoretical H1-HA–derived 15mer peptide was calculated using the IEDB tool for MHC-II binding prediction (http://tools.iedb.org/mhcii/; Paul et al., 2015). Donor-tailored analyses were performed using IEDB’s recommended method and considering the set of MHC-II alleles carried by each donor at the following loci: HLA-DRB1, HLA-DRB3/4/5 (if associated), HLA-DQA1/DQB1 in cis- or trans-pairing, and HLA-DPA1/DPB1 in cis- or trans-pairing. Top scoring H1-HA 15mer peptides for each donor were selected based on percentile rank calculated by comparison to a large set of random natural peptides. APL was computed as described in Mettu et al. (2016). Briefly, an aggregate z-score of conformational stability was determined for each H1-HA residue by integrating four structural parameters obtained from the 3D structure of postfusion HA resolved by x-ray diffraction (PDB codes: 3LZG for HA1 domain [Xu et al., 2010]; 1HTM for HA2 domain in the postfusion conformation [Bullough et al., 1994]). The z-score statistic was then used to calculate an APL for each theoretical H1-HA 15mer peptide, following the rationale that the liberation of antigenic peptides might be facilitated by surrounding unstable regions that are readily unfolded and targeted by endosomal proteases. For the optimization of combined predictors, we systematically performed peptide binding affinity predictions of each theoretical H1-HA 15mer peptide using IEDB for the MHC-II alleles carried by each donor, considering for each peptide the best scoring affinity within each group of MHC-II alleles. Using the set of epitopes recognized by memory T cells of each donor as true positives, we computed ROC curves for each predictive model and calculated the corresponding AUROC. Combined predictors for each donor were then built by iteratively weighting the contributions of epitope likelihood based on structural accessibility and peptide binding affinity to MHC-II, until we could maximize the AUROC value.

### Statistical analysis

Statistical analyses were performed using GraphPad Prism 8 software or R software v3.5.1. EC_50_ (ng/ml) and *K_d_* (ng/ml) values were calculated by nonlinear regression curve fit (4PL with automatic outlier elimination) using GraphPad Prism 8 software. Significance was assigned at P < 0.05, unless stated otherwise. Specific tests are indicated in the figure legends for each comparison.

### Online supplemental material

Fig. S1 shows in representative T cell clones blocking experiments with anti–HLA-DR, anti–HLA-DP, and anti–HLA-DQ antibodies to determine MHC-II restriction. It also shows MHC-II binding affinity of H1-HA peptides recognized by HLA-DR–restricted T cell clones from donor HD1 as measured in vitro. Fig. S2 shows the ability of a panel of H1-HA–reactive T cell clones to cross-react to HAs from different influenza A strains. Fig. S3 shows the functional avidities for peptide and naturally processed H1-HA of T cell clones isolated from the memory or the naive compartment determined by stimulation with titrated doses of peptides or recombinant H1-HA. Fig. S4 reports an analysis of MHC-II eluted H1-HA peptides measured by MS and of MHC-II H1-HA presented peptides to T cell clones of anti-head or anti-stem EBV-B cell clones. Fig. S5 reports the theoretical H1-HA 15mer peptide predicted to bind to selected MHC-II alleles using the IEDB tool and a the number of IEDB-predicted peptides and MHC-II eluted peptides measured by MS-based peptidomics found to be recognized by T cells. Table S1 shows epitope mapping of H1-HA–reactive T cell clones isolated from memory CD4^+^ T cell subsets of donor HD1. Table S2 shows TCR Vβ sequence and epitope specificity of H1-HA–reactive T cell clones isolated from CD4^+^ memory (Tcm, Tem, or cTfh) T cell compartment. Table S3 shows HLA class II typing of the four HDs included in this study. Table S4 shows TCR Vβ sequence and epitope specificity of H1-HA–reactive T cell clones isolated from the CD4^+^ naive T cell compartment. Table S5 lists H1-HA peptides identified by MS-based MHC-II peptidomics in donor HD1. Table S6 lists H1-HA peptides identified by MS-based MHC-II peptidomics in donors HD2–HD4.

## Supplementary Material

Table S1 shows epitope mapping of H1-HA–reactive T cell clones isolated from memory CD4^+^ T cell subsets of donor HD1.

Table S2 shows TCR Vβ sequence and epitope specificity of H1-HA–reactive T cell clones isolated from CD4^+^ memory (Tcm, Tem, or cTfh) T cell compartment.

Table S3 shows HLA class II typing of the four HDs included in this study.

Table S4 shows TCR Vβ sequence and epitope specificity of H1-HA–reactive T cell clones isolated from the CD4^+^ naive T cell compartment.

Table S5 lists H1-HA peptides identified by MS-based MHC-II peptidomics in donor HD1.

Table S6 lists H1-HA peptides identified by MS-based MHC-II peptidomics in donors HD2–HD4.
