# Supplementary material for: Deciphering and predicting CD4+ T cell immunodominance of influenza virus hemagglutinin
Source: J Exp Med. 2020 Jul 9;217(10):e20200206. doi: 10.1084/jem.20200206 (PMC7537397; doi:10.1084/jem.20200206)
Supplement: Table S1 — shows epitope mapping of H1-HA–reactive T cell clones isolated from memory CD4+ T cell subsets of donor HD1. [file JEM_20200206_TableS1.docx]

**Table S1.** Epitope mapping of H1-HA–reactive T cell clones isolated from memory CD4^+^ T cell subsets of donor HD1

| **Donor** | **Subset** | **Clone ID** | **Peptide** | **Start** | **Stop** | **Length** | **No Ag (Cpm)** | **Peptide (Cpm)** |
| --- | --- | --- | --- | --- | --- | --- | --- | --- |
| HD1-t1 | Tem | D2 | TFATANADTLCIGYH | 11 | 25 | 15 | 96 | 2780 |
| HD1-t1 | Tcm | C9 | KGKEVLVLWGIHHPS | 186 | 200 | 15 | 35 | 16800 |
| HD1-t1 | Tcm | F9 | KGKEVLVLWGIHHPS | 186 | 200 | 15 | 122 | 37220 |
| HD1-t1 | Tcm | G3 | KGKEVLVLWGIHHPS | 186 | 200 | 15 | 56 | 22389 |
| HD1-t1 | Tcm | G8 | KGKEVLVLWGIHHPS | 186 | 200 | 15 | 80 | 1087 |
| HD1-t1 | Tcm | E5 | KGKEVLVLWGIHHPS | 186 | 200 | 15 | 57 | 34909 |
| HD1-t1 | Tcm | M8 | KGKEVLVLWGIHHPS | 186 | 200 | 15 | 70 | 38751 |
| HD1-t1 | Tcm | N5 | KGKEVLVLWGIHHPS | 186 | 200 | 15 | 9 | 7557 |
| HD1-t1 | Tcm | G5 | KGKEVLVLWGIHHPS | 186 | 200 | 15 | 74 | 3209 |
| HD1-t1 | Tcm | 1_C10 | KGKEVLVLWGIHHPS | 186 | 200 | 15 | 112 | 31556 |
| HD1-t1 | Tem | C10 | KGKEVLVLWGIHHPS | 186 | 200 | 15 | 23 | 1921 |
| HD1-t1 | cTfh | 1_E2 | KGKEVLVLWGIHHPSTSADQ | 186 | 205 | 20 | 152 | 5961 |
| HD1-t1 | Tcm | H11 | KGKEVLVLWGIHHPSTSADQ | 186 | 205 | 20 | 9 | 7607 |
| HD1-t1 | Tcm | B10 | KFKPEIAIRPKVRDQ | 226 | 240 | 15 | 37 | 52927 |
| HD1-t1 | Tcm | E10 | KFKPEIAIRPKVRDQ | 226 | 240 | 15 | 28 | 13504 |
| HD1-t1 | Tcm | H10 | KFKPEIAIRPKVRDQ | 226 | 240 | 15 | 17 | 14525 |
| HD1-t1 | cTfh | B10 | EGRMNYYWTLVEPGD | 241 | 255 | 15 | 30 | 1384 |
| HD1-t1 | Tcm | E11 | EGRMNYYWTLVEPGD | 241 | 255 | 15 | 2 | 2194 |
| HD1-t1 | cTfh | B5 | EGRMNYYWTLVEPGDKITFE | 241 | 260 | 20 | 133 | 2276 |
| HD1-t1 | cTfh | C3 | EGRMNYYWTLVEPGDKITFE | 241 | 260 | 20 | 26 | 2806 |
| HD1-t1 | cTfh | D5 | EGRMNYYWTLVEPGDKITFE | 241 | 260 | 20 | 26 | 1411 |
| HD1-t1 | Tcm | C2 | EGRMNYYWTLVEPGDKITFE | 241 | 260 | 20 | 17 | 1545 |
| HD1-t1 | Tcm | C4 | EGRMNYYWTLVEPGDKITFE | 241 | 260 | 20 | 87 | 4061 |
| HD1-t1 | Tcm | C8 | EGRMNYYWTLVEPGDKITFE | 241 | 260 | 20 | 6 | 2473 |
| HD1-t1 | Tcm | H2 | EGRMNYYWTLVEPGDKITFE | 241 | 260 | 20 | 30 | 4149 |
| HD1-t1 | Tcm | H7 | EGRMNYYWTLVEPGDKITFE | 241 | 260 | 20 | 56 | 8047 |
| HD1-t1 | Tcm | G9 | EGRMNYYWTLVEPGDKITFE | 241 | 260 | 20 | 15 | 6585 |
| HD1-t2 | Tcm | P2_H1 | EGRMNYYWTLVEPGDKITFE | 241 | 260 | 20 | 1057 | 13480 |
| HD1-t2 | Tem | P1_B3 | EGRMNYYWTLVEPGDKITFE | 241 | 260 | 20 | 779 | 6019 |
| HD1-t2 | Tem | P1_B6 | EGRMNYYWTLVEPGDKITFE | 241 | 260 | 20 | 999 | 3372 |
| HD1-t2 | Tem | P1_D11 | EGRMNYYWTLVEPGDKITFE | 241 | 260 | 20 | 831 | 15987 |
| HD1-t2 | Tem | P1_F12 | EGRMNYYWTLVEPGDKITFE | 241 | 260 | 20 | 1079 | 25246 |
| HD1-t2 | Tem | P1_H2 | EGRMNYYWTLVEPGDKITFE | 241 | 260 | 20 | 860 | 4606 |
| HD1-t2 | Tem | P1_H3 | EGRMNYYWTLVEPGDKITFE | 241 | 260 | 20 | 711 | 2737 |
| HD1-t2 | Tem | P2_A5 | EGRMNYYWTLVEPGDKITFE | 241 | 260 | 20 | 960 | 9814 |
| HD1-t2 | Tem | P2_B2 | EGRMNYYWTLVEPGDKITFE | 241 | 260 | 20 | 1471 | 24848 |
| HD1-t2 | Tem | P2_B10 | EGRMNYYWTLVEPGDKITFE | 241 | 260 | 20 | 1898 | 15899 |
| HD1-t2 | Tem | P2_D9 | EGRMNYYWTLVEPGDKITFE | 241 | 260 | 20 | 610 | 5304 |
| HD1-t2 | Tem | P2_F3 | EGRMNYYWTLVEPGDKITFE | 241 | 260 | 20 | 746 | 7698 |
| HD1-t2 | Tem | P2_H1 | EGRMNYYWTLVEPGDKITFE | 241 | 260 | 20 | 934 | 3470 |
| HD1-t1 | Tcm | N2 | VEPGDKITFEATGNL | 251 | 265 | 15 | 40 | 6888 |
| HD1-t1 | Tcm | I11 | VVPRYAFAMERNAGS | 266 | 280 | 15 | 20 | 43000 |
| HD1-t1 | Tcm | B11 | QNAIDEITNKVNSVI | 386 | 400 | 15 | 11 | 3949 |
| HD1-t1 | cTfh | D10 | VNSVIEKMNTQFTAV | 396 | 410 | 15 | 2 | 24126 |
| HD1-t1 | cTfh | D4 | VNSVIEKMNTQFTAV | 396 | 410 | 15 | 27 | 8367 |
| HD1-t1 | Tcm | C7 | VNSVIEKMNTQFTAV | 396 | 410 | 15 | 111 | 31432 |
| HD1-t1 | cTfh | 1_E6 | EKMNTQFTAVGKEFN | 401 | 415 | 15 | 50 | 1516 |
| HD1-t2 | Tem | P1_E9 | EKMNTQFTAVGKEFN | 401 | 415 | 15 | 705 | 3439 |
| HD1-t2 | Tem | P2_C4 | EKMNTQFTAVGKEFN | 401 | 415 | 15 | 607 | 4359 |
| HD1-t2 | Tem | P2_C5 | EKMNTQFTAVGKEFN | 401 | 415 | 15 | 1693 | 13277 |
| HD1-t2 | Tem | P2_E4 | EKMNTQFTAVGKEFN | 401 | 415 | 15 | 546 | 3244 |
| HD1-t2 | Tem | P2_H6 | EKMNTQFTAVGKEFN | 401 | 415 | 15 | 513 | 2298 |
| HD1-t2 | Tem | P2_H9 | EKMNTQFTAVGKEFN | 401 | 415 | 15 | 557 | 2334 |
| HD1-t1 | cTfh | B11 | EKMNTQFTAVGKEFNHLEKR | 401 | 420 | 20 | 41 | 10207 |
| HD1-t1 | cTfh | B2 | EKMNTQFTAVGKEFNHLEKR | 401 | 420 | 20 | 28 | 8679 |
| HD1-t1 | cTfh | B4 | EKMNTQFTAVGKEFNHLEKR | 401 | 420 | 20 | 11 | 9663 |
| HD1-t1 | cTfh | C4 | EKMNTQFTAVGKEFNHLEKR | 401 | 420 | 20 | 14 | 10216 |
| HD1-t1 | cTfh | C7 | EKMNTQFTAVGKEFNHLEKR | 401 | 420 | 20 | 15 | 6012 |
| HD1-t1 | cTfh | C8 | EKMNTQFTAVGKEFNHLEKR | 401 | 420 | 20 | 37 | 4298 |
| HD1-t1 | cTfh | D11 | EKMNTQFTAVGKEFNHLEKR | 401 | 420 | 20 | 35 | 6440 |
| HD1-t2 | cTfh | P2_B8 | EKMNTQFTAVGKEFNHLEKR | 401 | 420 | 20 | 744 | 3175 |
| HD1-t2 | cTfh | P2_B9 | EKMNTQFTAVGKEFNHLEKR | 401 | 420 | 20 | 670 | 5584 |
| HD1-t2 | cTfh | P2_C1 | EKMNTQFTAVGKEFNHLEKR | 401 | 420 | 20 | 1049 | 10934 |
| HD1-t2 | cTfh | P2_C5 | EKMNTQFTAVGKEFNHLEKR | 401 | 420 | 20 | 636 | 6484 |
| HD1-t2 | cTfh | P2_C7 | EKMNTQFTAVGKEFNHLEKR | 401 | 420 | 20 | 636 | 5305 |
| HD1-t2 | cTfh | P2_E3 | EKMNTQFTAVGKEFNHLEKR | 401 | 420 | 20 | 653 | 2090 |
| HD1-t2 | cTfh | P2_E12 | EKMNTQFTAVGKEFNHLEKR | 401 | 420 | 20 | 714 | 29826 |
| HD1-t2 | cTfh | P2_F7 | EKMNTQFTAVGKEFNHLEKR | 401 | 420 | 20 | 666 | 18331 |
| HD1-t2 | cTfh | P2_G1 | EKMNTQFTAVGKEFNHLEKR | 401 | 420 | 20 | 558 | 82246 |
| HD1-t2 | cTfh | P3_A8 | EKMNTQFTAVGKEFNHLEKR | 401 | 420 | 20 | 536 | 5856 |
| HD1-t2 | cTfh | P3_B8 | EKMNTQFTAVGKEFNHLEKR | 401 | 420 | 20 | 548 | 15338 |
| HD1-t2 | cTfh | P3_B11 | EKMNTQFTAVGKEFNHLEKR | 401 | 420 | 20 | 710 | 9439 |
| HD1-t2 | cTfh | P3_C6 | EKMNTQFTAVGKEFNHLEKR | 401 | 420 | 20 | 615 | 1859 |
| HD1-t2 | cTfh | P3_C10 | EKMNTQFTAVGKEFNHLEKR | 401 | 420 | 20 | 518 | 35719 |
| HD1-t2 | cTfh | P3_D11 | EKMNTQFTAVGKEFNHLEKR | 401 | 420 | 20 | 555 | 26321 |
| HD1-t2 | cTfh | P3_E11 | EKMNTQFTAVGKEFNHLEKR | 401 | 420 | 20 | 522 | 41415 |
| HD1-t2 | cTfh | P3_F5 | EKMNTQFTAVGKEFNHLEKR | 401 | 420 | 20 | 805 | 2621 |
| HD1-t2 | cTfh | P3_G2 | EKMNTQFTAVGKEFNHLEKR | 401 | 420 | 20 | 580 | 2138 |
| HD1-t2 | cTfh | P3_G9 | EKMNTQFTAVGKEFNHLEKR | 401 | 420 | 20 | 843 | 16556 |
| HD1-t2 | cTfh | P3_H9 | EKMNTQFTAVGKEFNHLEKR | 401 | 420 | 20 | 783 | 9039 |
| HD1-t2 | cTfh | P1_B6 | EKMNTQFTAVGKEFNHLEKR | 401 | 420 | 20 | 757 | 21726 |
| HD1-t2 | cTfh | P1_D1 | EKMNTQFTAVGKEFNHLEKR | 401 | 420 | 20 | 762 | 31463 |
| HD1-t2 | cTfh | P1_D6 | EKMNTQFTAVGKEFNHLEKR | 401 | 420 | 20 | 813 | 8569 |
| HD1-t2 | cTfh | P1_E12 | EKMNTQFTAVGKEFNHLEKR | 401 | 420 | 20 | 636 | 40637 |
| HD1-t2 | cTfh | P1_F6 | EKMNTQFTAVGKEFNHLEKR | 401 | 420 | 20 | 695 | 5182 |
| HD1-t2 | cTfh | P1_F8 | EKMNTQFTAVGKEFNHLEKR | 401 | 420 | 20 | 717 | 38826 |
| HD1-t2 | cTfh | P1_G8 | EKMNTQFTAVGKEFNHLEKR | 401 | 420 | 20 | 589 | 1880 |
| HD1-t2 | cTfh | P1_G11 | EKMNTQFTAVGKEFNHLEKR | 401 | 420 | 20 | 545 | 48497 |
| HD1-t2 | cTfh | P2_A2 | EKMNTQFTAVGKEFNHLEKR | 401 | 420 | 20 | 544 | 19086 |
| HD1-t2 | cTfh | P2_B1 | EKMNTQFTAVGKEFNHLEKR | 401 | 420 | 20 | 558 | 42241 |
| HD1-t2 | cTfh | P2_B2 | EKMNTQFTAVGKEFNHLEKR | 401 | 420 | 20 | 588 | 22689 |
| HD1-t2 | cTfh | P2_B3 | EKMNTQFTAVGKEFNHLEKR | 401 | 420 | 20 | 439 | 22995 |
| HD1-t2 | cTfh | P2_D9 | EKMNTQFTAVGKEFNHLEKR | 401 | 420 | 20 | 822 | 55302 |
| HD1-t2 | cTfh | P2_G8 | EKMNTQFTAVGKEFNHLEKR | 401 | 420 | 20 | 896 | 33606 |
| HD1-t2 | cTfh | P3_A2 | EKMNTQFTAVGKEFNHLEKR | 401 | 420 | 20 | 1058 | 31877 |
| HD1-t2 | cTfh | P3_C6 | EKMNTQFTAVGKEFNHLEKR | 401 | 420 | 20 | 860 | 18204 |
| HD1-t2 | cTfh | P3_C10 | EKMNTQFTAVGKEFNHLEKR | 401 | 420 | 20 | 839 | 76180 |
| HD1-t2 | cTfh | P3_G11 | EKMNTQFTAVGKEFNHLEKR | 401 | 420 | 20 | 653 | 38717 |
| HD1-t2 | cTfh | P1_B7 | EKMNTQFTAVGKEFNHLEKR | 401 | 420 | 20 | 1114 | 15735 |
| HD1-t1 | Tcm | M4 | EKMNTQFTAVGKEFNHLEKR | 401 | 420 | 20 | 59 | 18915 |
| HD1-t1 | Tcm | G5 | EKMNTQFTAVGKEFNHLEKR | 401 | 420 | 20 | 4 | 11291 |
| HD1-t2 | Tcm | P1_F10 | EKMNTQFTAVGKEFNHLEKR | 401 | 420 | 20 | 527 | 9991 |
| HD1-t2 | Tcm | P2_B8 | EKMNTQFTAVGKEFNHLEKR | 401 | 420 | 20 | 500 | 9565 |
| HD1-t1 | Tcm | 1_F3 | EKMNTQFTAVGKEFNHLEKR | 401 | 420 | 20 | 182 | 4927 |
| HD1-t2 | Tem | P1_C2 | EKMNTQFTAVGKEFNHLEKR | 401 | 420 | 20 | 695 | 11262 |
| HD1-t2 | Tem | P1_C5 | EKMNTQFTAVGKEFNHLEKR | 401 | 420 | 20 | 829 | 7612 |
| HD1-t2 | Tem | P1_C8 | EKMNTQFTAVGKEFNHLEKR | 401 | 420 | 20 | 583 | 12065 |
| HD1-t2 | Tem | P1_C10 | EKMNTQFTAVGKEFNHLEKR | 401 | 420 | 20 | 566 | 125856 |
| HD1-t2 | Tem | P1_D6 | EKMNTQFTAVGKEFNHLEKR | 401 | 420 | 20 | 562 | 4678 |
| HD1-t2 | Tem | P1_D7 | EKMNTQFTAVGKEFNHLEKR | 401 | 420 | 20 | 727 | 2219 |
| HD1-t2 | Tem | P1_D12 | EKMNTQFTAVGKEFNHLEKR | 401 | 420 | 20 | 804 | 85168 |
| HD1-t2 | Tem | P1_E1 | EKMNTQFTAVGKEFNHLEKR | 401 | 420 | 20 | 701 | 32527 |
| HD1-t2 | Tem | P1_F2 | EKMNTQFTAVGKEFNHLEKR | 401 | 420 | 20 | 636 | 12684 |
| HD1-t2 | Tem | P1_H8 | EKMNTQFTAVGKEFNHLEKR | 401 | 420 | 20 | 506 | 13317 |
| HD1-t2 | Tem | P2_A10 | EKMNTQFTAVGKEFNHLEKR | 401 | 420 | 20 | 749 | 2090 |
| HD1-t2 | Tem | P2_C6 | EKMNTQFTAVGKEFNHLEKR | 401 | 420 | 20 | 549 | 5318 |
| HD1-t2 | Tem | P2_C10 | EKMNTQFTAVGKEFNHLEKR | 401 | 420 | 20 | 705 | 3678 |
| HD1-t2 | Tem | P2_C12 | EKMNTQFTAVGKEFNHLEKR | 401 | 420 | 20 | 596 | 5462 |
| HD1-t2 | Tem | P2_E5 | EKMNTQFTAVGKEFNHLEKR | 401 | 420 | 20 | 504 | 1975 |
| HD1-t2 | Tem | P2_E11 | EKMNTQFTAVGKEFNHLEKR | 401 | 420 | 20 | 614 | 6112 |
| HD1-t2 | Tem | P2_F2 | EKMNTQFTAVGKEFNHLEKR | 401 | 420 | 20 | 732 | 53825 |
| HD1-t2 | Tem | P2_F6 | EKMNTQFTAVGKEFNHLEKR | 401 | 420 | 20 | 732 | 9841 |
| HD1-t2 | Tem | P2_F12 | EKMNTQFTAVGKEFNHLEKR | 401 | 420 | 20 | 452 | 21091 |
| HD1-t2 | Tem | P2_G9 | EKMNTQFTAVGKEFNHLEKR | 401 | 420 | 20 | 363 | 2675 |
| HD1-t2 | Tem | P3_B5 | EKMNTQFTAVGKEFNHLEKR | 401 | 420 | 20 | 452 | 6693 |
| HD1-t2 | Tem | P3_B8 | EKMNTQFTAVGKEFNHLEKR | 401 | 420 | 20 | 418 | 15288 |
| HD1-t2 | Tem | P1_B12 | EKMNTQFTAVGKEFNHLEKR | 401 | 420 | 20 | 749 | 2173 |
| HD1-t2 | Tem | P1_B5 | EKMNTQFTAVGKEFNHLEKR | 401 | 420 | 20 | 822 | 3397 |
| HD1-t2 | Tem | P1_F3 | EKMNTQFTAVGKEFNHLEKR | 401 | 420 | 20 | 614 | 4137 |
| HD1-t2 | Tem | P2_C4 | EKMNTQFTAVGKEFNHLEKR | 401 | 420 | 20 | 708 | 3788 |
| HD1-t2 | Tem | P2_C5 | EKMNTQFTAVGKEFNHLEKR | 401 | 420 | 20 | 468 | 5469 |
| HD1-t2 | Tem | P2_E12 | EKMNTQFTAVGKEFNHLEKR | 401 | 420 | 20 | 555 | 3705 |
| HD1-t2 | Tem | P2_H6 | EKMNTQFTAVGKEFNHLEKR | 401 | 420 | 20 | 546 | 7255 |
| HD1-t2 | Tem | P2_H9 | EKMNTQFTAVGKEFNHLEKR | 401 | 420 | 20 | 404 | 2090 |
| HD1-t2 | Tem | P2_H11 | EKMNTQFTAVGKEFNHLEKR | 401 | 420 | 20 | 1128 | 36506 |
| HD1-t2 | cTfh | P3_D8 | QFTAVGKEFNHLEKR | 406 | 420 | 15 | 568 | 2965 |
| HD1-t2 | Tem | P1_B1 | QFTAVGKEFNHLEKR | 406 | 420 | 15 | 749 | 63510 |
| HD1-t2 | Tem | P1_B5 | QFTAVGKEFNHLEKR | 406 | 420 | 15 | 688 | 16281 |
| HD1-t2 | Tem | P1_B12 | QFTAVGKEFNHLEKR | 406 | 420 | 15 | 862 | 7668 |
| HD1-t2 | Tem | P1_D4 | QFTAVGKEFNHLEKR | 406 | 420 | 15 | 558 | 24127 |
| HD1-t2 | Tem | P1_F10 | QFTAVGKEFNHLEKR | 406 | 420 | 15 | 697 | 8586 |
| HD1-t2 | Tem | P2_B8 | QFTAVGKEFNHLEKR | 406 | 420 | 15 | 649 | 4252 |
| HD1-t2 | Tem | P2_E12 | QFTAVGKEFNHLEKR | 406 | 420 | 15 | 1189 | 11381 |
| HD1-t2 | Tem | P2_G6 | QFTAVGKEFNHLEKR | 406 | 420 | 15 | 976 | 9414 |
| HD1-t2 | Tem | P1_B1 | QFTAVGKEFNHLEKR | 406 | 420 | 15 | 873 | 59881 |
| HD1-t2 | Tem | P1_D4 | QFTAVGKEFNHLEKR | 406 | 420 | 15 | 658 | 25169 |
| HD1-t2 | Tem | P1_F10 | QFTAVGKEFNHLEKR | 406 | 420 | 15 | 1237 | 25675 |
| HD1-t2 | Tem | P2_G6 | QFTAVGKEFNHLEKR | 406 | 420 | 15 | 683 | 23579 |
| HD1-t2 | cTfh | P1_C8 | GKEFNHLEKRIENLN | 411 | 425 | 15 | 1297 | 6809 |
| HD1-t2 | cTfh | P1_D11 | GKEFNHLEKRIENLN | 411 | 425 | 15 | 609 | 9181 |
| HD1-t2 | cTfh | P1_B2 | GKEFNHLEKRIENLN | 411 | 425 | 15 | 949 | 16047 |
| HD1-t1 | Tem | C3 | GKEFNHLEKRIENLN | 411 | 425 | 15 | 39 | 3202 |
| HD1-t1 | Tem | C6 | GKEFNHLEKRIENLN | 411 | 425 | 15 | 48 | 4812 |
| HD1-t1 | Tem | C8 | GKEFNHLEKRIENLN | 411 | 425 | 15 | 75 | 1054 |
| HD1-t1 | Tem | D9 | GKEFNHLEKRIENLN | 411 | 425 | 15 | 9 | 908 |
| HD1-t1 | Tem | E3 | GKEFNHLEKRIENLN | 411 | 425 | 15 | 24 | 10746 |
| HD1-t2 | Tem | P1_A10 | GKEFNHLEKRIENLN | 411 | 425 | 15 | 645 | 11100 |
| HD1-t2 | Tem | P1_C4 | GKEFNHLEKRIENLN | 411 | 425 | 15 | 671 | 19960 |
| HD1-t2 | Tem | P1_C7 | GKEFNHLEKRIENLN | 411 | 425 | 15 | 737 | 5220 |
| HD1-t2 | Tem | P1_G9 | GKEFNHLEKRIENLN | 411 | 425 | 15 | 505 | 10000 |
| HD1-t2 | Tem | P1_G11 | GKEFNHLEKRIENLN | 411 | 425 | 15 | 575 | 4441 |
| HD1-t2 | Tem | P2_A3 | GKEFNHLEKRIENLN | 411 | 425 | 15 | 705 | 2983 |
| HD1-t2 | Tem | P2_A6 | GKEFNHLEKRIENLN | 411 | 425 | 15 | 735 | 15644 |
| HD1-t2 | Tem | P2_A11 | GKEFNHLEKRIENLN | 411 | 425 | 15 | 557 | 14299 |
| HD1-t2 | Tem | P2_B1 | GKEFNHLEKRIENLN | 411 | 425 | 15 | 827 | 7762 |
| HD1-t2 | Tem | P2_B11 | GKEFNHLEKRIENLN | 411 | 425 | 15 | 666 | 11052 |
| HD1-t2 | Tem | P2_D8 | GKEFNHLEKRIENLN | 411 | 425 | 15 | 486 | 3339 |
| HD1-t2 | Tem | P2_E3 | GKEFNHLEKRIENLN | 411 | 425 | 15 | 589 | 13167 |
| HD1-t2 | Tem | P2_E6 | GKEFNHLEKRIENLN | 411 | 425 | 15 | 572 | 7822 |
| HD1-t2 | Tem | P2_E7 | GKEFNHLEKRIENLN | 411 | 425 | 15 | 513 | 4700 |
| HD1-t2 | Tem | P2_G5 | GKEFNHLEKRIENLN | 411 | 425 | 15 | 482 | 2349 |
| HD1-t2 | Tem | P2_G10 | GKEFNHLEKRIENLN | 411 | 425 | 15 | 436 | 9767 |
| HD1-t2 | Tem | P3_B3 | GKEFNHLEKRIENLN | 411 | 425 | 15 | 572 | 3352 |
| HD1-t2 | Tem | P3_B7 | GKEFNHLEKRIENLN | 411 | 425 | 15 | 590 | 2656 |
| HD1-t2 | Tem | P1_E3 | GKEFNHLEKRIENLN | 411 | 425 | 15 | 826 | 8149 |
| HD1-t2 | Tem | P2_C2 | GKEFNHLEKRIENLN | 411 | 425 | 15 | 1167 | 4046 |
| HD1-t2 | Tem | P2_D5 | GKEFNHLEKRIENLN | 411 | 425 | 15 | 839 | 35993 |
| HD1-t2 | Tem | P2_D6 | GKEFNHLEKRIENLN | 411 | 425 | 15 | 839 | 21265 |
| HD1-t2 | Tem | P2_D7 | GKEFNHLEKRIENLN | 411 | 425 | 15 | 873 | 9431 |
| HD1-t2 | cTfh | P1_B8 | GKEFNHLEKRIENLNKKVDD | 411 | 430 | 20 | 1557 | 5049 |
| HD1-t2 | cTfh | P1_B9 | GKEFNHLEKRIENLNKKVDD | 411 | 430 | 20 | 1335 | 10451 |
| HD1-t2 | cTfh | P1_C10 | GKEFNHLEKRIENLNKKVDD | 411 | 430 | 20 | 2712 | 11114 |
| HD1-t2 | cTfh | P1_D3 | GKEFNHLEKRIENLNKKVDD | 411 | 430 | 20 | 3428 | 12258 |
| HD1-t2 | cTfh | P1_D8 | GKEFNHLEKRIENLNKKVDD | 411 | 430 | 20 | 1607 | 15770 |
| HD1-t2 | cTfh | P1_E6 | GKEFNHLEKRIENLNKKVDD | 411 | 430 | 20 | 1541 | 15125 |
| HD1-t2 | cTfh | P2_G3 | GKEFNHLEKRIENLNKKVDD | 411 | 430 | 20 | 1389 | 7065 |
| HD1-t2 | cTfh | P3_B10 | GKEFNHLEKRIENLNKKVDD | 411 | 430 | 20 | 1135 | 5140 |
| HD1-t2 | cTfh | P3_D5 | GKEFNHLEKRIENLNKKVDD | 411 | 430 | 20 | 1730 | 6265 |
| HD1-t2 | cTfh | P3_F3 | GKEFNHLEKRIENLNKKVDD | 411 | 430 | 20 | 1126 | 11392 |
| HD1-t2 | cTfh | P3_G4 | GKEFNHLEKRIENLNKKVDD | 411 | 430 | 20 | 3916 | 19325 |
| HD1-t2 | cTfh | P3_G7 | GKEFNHLEKRIENLNKKVDD | 411 | 430 | 20 | 961 | 5573 |
| HD1-t2 | cTfh | P3_H7 | GKEFNHLEKRIENLNKKVDD | 411 | 430 | 20 | 1635 | 22621 |
| HD1-t2 | cTfh | P1_G2 | GKEFNHLEKRIENLNKKVDD | 411 | 430 | 20 | 1385 | 12365 |
| HD1-t2 | Tcm | P1_B10 | GKEFNHLEKRIENLNKKVDD | 411 | 430 | 20 | 1376 | 17180 |
| HD1-t2 | Tcm | P1_D9 | GKEFNHLEKRIENLNKKVDD | 411 | 430 | 20 | 1643 | 10950 |
| HD1-t2 | Tcm | P1_E4 | GKEFNHLEKRIENLNKKVDD | 411 | 430 | 20 | 1422 | 14131 |
| HD1-t2 | Tcm | P1_F5 | GKEFNHLEKRIENLNKKVDD | 411 | 430 | 20 | 3510 | 10997 |
| HD1-t2 | Tcm | P1_G8 | GKEFNHLEKRIENLNKKVDD | 411 | 430 | 20 | 1668 | 29311 |
| HD1-t2 | Tcm | P1_G9 | GKEFNHLEKRIENLNKKVDD | 411 | 430 | 20 | 636 | 2660 |
| HD1-t2 | Tcm | P2_C8 | GKEFNHLEKRIENLNKKVDD | 411 | 430 | 20 | 1439 | 4397 |
| HD1-t2 | Tcm | P2_E8 | GKEFNHLEKRIENLNKKVDD | 411 | 430 | 20 | 1259 | 22230 |
| HD1-t2 | Tcm | P2_E11 | GKEFNHLEKRIENLNKKVDD | 411 | 430 | 20 | 2595 | 8928 |
| HD1-t2 | Tcm | P2_F10 | GKEFNHLEKRIENLNKKVDD | 411 | 430 | 20 | 3821 | 22210 |
| HD1-t2 | Tcm | P2_H7 | GKEFNHLEKRIENLNKKVDD | 411 | 430 | 20 | 1602 | 5226 |
| HD1-t2 | Tcm | P3_B6 | GKEFNHLEKRIENLNKKVDD | 411 | 430 | 20 | 1607 | 36033 |
| HD1-t2 | Tcm | P3_C4 | GKEFNHLEKRIENLNKKVDD | 411 | 430 | 20 | 1494 | 4869 |
| HD1-t2 | Tcm | P1_C5 | GKEFNHLEKRIENLNKKVDD | 411 | 430 | 20 | 1567 | 5604 |
| HD1-t2 | Tcm | P1_G4 | GKEFNHLEKRIENLNKKVDD | 411 | 430 | 20 | 4132 | 13350 |
| HD1-t2 | Tcm | P2_E9 | GKEFNHLEKRIENLNKKVDD | 411 | 430 | 20 | 1771 | 14502 |
| HD1-t2 | Tem | P2_D5 | GKEFNHLEKRIENLNKKVDD | 411 | 430 | 20 | 623 | 19462 |
| HD1-t2 | Tem | P1_C12 | GKEFNHLEKRIENLNKKVDD | 411 | 430 | 20 | 1394 | 7942 |
| HD1-t2 | Tem | P2_C11 | GKEFNHLEKRIENLNKKVDD | 411 | 430 | 20 | 2523 | 21745 |
| HD1-t2 | Tem | P2_D4 | GKEFNHLEKRIENLNKKVDD | 411 | 430 | 20 | 1153 | 11388 |
| HD1-t2 | Tem | P2_G1 | GKEFNHLEKRIENLNKKVDD | 411 | 430 | 20 | 1825 | 28035 |
| HD1-t2 | Tem | P2_H7 | GKEFNHLEKRIENLNKKVDD | 411 | 430 | 20 | 1588 | 8809 |
| HD1-t1 | cTfh | B3 | HLEKRIENLNKKVDD | 416 | 430 | 15 | 2 | 8812 |
| HD1-t1 | cTfh | C10 | HLEKRIENLNKKVDD | 416 | 430 | 15 | 145 | 2412 |
| HD1-t1 | cTfh | C11 | HLEKRIENLNKKVDD | 416 | 430 | 15 | 22 | 19297 |
| HD1-t1 | cTfh | C6 | HLEKRIENLNKKVDD | 416 | 430 | 15 | 9 | 28233 |
| HD1-t1 | cTfh | E2 | HLEKRIENLNKKVDD | 416 | 430 | 15 | 17 | 16733 |
| HD1-t2 | cTfh | P2_B11 | HLEKRIENLNKKVDD | 416 | 430 | 15 | 636 | 9331 |
| HD1-t2 | cTfh | P2_C6 | HLEKRIENLNKKVDD | 416 | 430 | 15 | 783 | 42993 |
| HD1-t2 | cTfh | P2_C8 | HLEKRIENLNKKVDD | 416 | 430 | 15 | 714 | 16421 |
| HD1-t2 | cTfh | P2_C9 | HLEKRIENLNKKVDD | 416 | 430 | 15 | 1068 | 10181 |
| HD1-t2 | cTfh | P2_C11 | HLEKRIENLNKKVDD | 416 | 430 | 15 | 649 | 7886 |
| HD1-t2 | cTfh | P2_D1 | HLEKRIENLNKKVDD | 416 | 430 | 15 | 458 | 2312 |
| HD1-t2 | cTfh | P2_D4 | HLEKRIENLNKKVDD | 416 | 430 | 15 | 1045 | 8908 |
| HD1-t2 | cTfh | P2_D5 | HLEKRIENLNKKVDD | 416 | 430 | 15 | 506 | 3719 |
| HD1-t2 | cTfh | P2_D8 | HLEKRIENLNKKVDD | 416 | 430 | 15 | 575 | 2233 |
| HD1-t2 | cTfh | P2_D12 | HLEKRIENLNKKVDD | 416 | 430 | 15 | 602 | 6771 |
| HD1-t2 | cTfh | P2_E2 | HLEKRIENLNKKVDD | 416 | 430 | 15 | 548 | 4468 |
| HD1-t2 | cTfh | P2_E5 | HLEKRIENLNKKVDD | 416 | 430 | 15 | 522 | 2094 |
| HD1-t2 | cTfh | P2_E8 | HLEKRIENLNKKVDD | 416 | 430 | 15 | 584 | 7408 |
| HD1-t2 | cTfh | P2_F1 | HLEKRIENLNKKVDD | 416 | 430 | 15 | 546 | 1667 |
| HD1-t2 | cTfh | P2_F2 | HLEKRIENLNKKVDD | 416 | 430 | 15 | 748 | 2565 |
| HD1-t2 | cTfh | P2_F4 | HLEKRIENLNKKVDD | 416 | 430 | 15 | 571 | 9583 |
| HD1-t2 | cTfh | P2_F6 | HLEKRIENLNKKVDD | 416 | 430 | 15 | 714 | 16973 |
| HD1-t2 | cTfh | P2_F9 | HLEKRIENLNKKVDD | 416 | 430 | 15 | 500 | 1637 |
| HD1-t2 | cTfh | P2_F12 | HLEKRIENLNKKVDD | 416 | 430 | 15 | 996 | 3867 |
| HD1-t2 | cTfh | P2_G4 | HLEKRIENLNKKVDD | 416 | 430 | 15 | 701 | 18411 |
| HD1-t2 | cTfh | P2_G6 | HLEKRIENLNKKVDD | 416 | 430 | 15 | 554 | 2664 |
| HD1-t2 | cTfh | P2_H4 | HLEKRIENLNKKVDD | 416 | 430 | 15 | 454 | 6014 |
| HD1-t2 | cTfh | P2_H7 | HLEKRIENLNKKVDD | 416 | 430 | 15 | 477 | 8635 |
| HD1-t2 | cTfh | P2_H11 | HLEKRIENLNKKVDD | 416 | 430 | 15 | 684 | 2588 |
| HD1-t2 | cTfh | P3_A4 | HLEKRIENLNKKVDD | 416 | 430 | 15 | 551 | 4145 |
| HD1-t2 | cTfh | P3_B2 | HLEKRIENLNKKVDD | 416 | 430 | 15 | 395 | 5750 |
| HD1-t2 | cTfh | P3_B3 | HLEKRIENLNKKVDD | 416 | 430 | 15 | 568 | 2775 |
| HD1-t2 | cTfh | P3_B6 | HLEKRIENLNKKVDD | 416 | 430 | 15 | 601 | 10210 |
| HD1-t2 | cTfh | P3_B9 | HLEKRIENLNKKVDD | 416 | 430 | 15 | 465 | 14841 |
| HD1-t2 | cTfh | P3_C3 | HLEKRIENLNKKVDD | 416 | 430 | 15 | 422 | 21295 |
| HD1-t2 | cTfh | P3_C4 | HLEKRIENLNKKVDD | 416 | 430 | 15 | 645 | 1738 |
| HD1-t2 | cTfh | P3_C7 | HLEKRIENLNKKVDD | 416 | 430 | 15 | 736 | 12330 |
| HD1-t2 | cTfh | P3_C8 | HLEKRIENLNKKVDD | 416 | 430 | 15 | 468 | 20087 |
| HD1-t2 | cTfh | P3_C9 | HLEKRIENLNKKVDD | 416 | 430 | 15 | 454 | 25808 |
| HD1-t2 | cTfh | P3_C11 | HLEKRIENLNKKVDD | 416 | 430 | 15 | 430 | 22544 |
| HD1-t2 | cTfh | P3_D6 | HLEKRIENLNKKVDD | 416 | 430 | 15 | 526 | 1597 |
| HD1-t2 | cTfh | P3_E2 | HLEKRIENLNKKVDD | 416 | 430 | 15 | 447 | 1515 |
| HD1-t2 | cTfh | P3_E3 | HLEKRIENLNKKVDD | 416 | 430 | 15 | 667 | 49977 |
| HD1-t2 | cTfh | P3_E5 | HLEKRIENLNKKVDD | 416 | 430 | 15 | 490 | 5587 |
| HD1-t2 | cTfh | P3_E9 | HLEKRIENLNKKVDD | 416 | 430 | 15 | 421 | 5007 |
| HD1-t2 | cTfh | P3_F4 | HLEKRIENLNKKVDD | 416 | 430 | 15 | 517 | 4743 |
| HD1-t2 | cTfh | P3_F6 | HLEKRIENLNKKVDD | 416 | 430 | 15 | 670 | 5717 |
| HD1-t2 | cTfh | P3_F8 | HLEKRIENLNKKVDD | 416 | 430 | 15 | 496 | 18268 |
| HD1-t2 | cTfh | P3_F9 | HLEKRIENLNKKVDD | 416 | 430 | 15 | 644 | 2795 |
| HD1-t2 | cTfh | P3_F10 | HLEKRIENLNKKVDD | 416 | 430 | 15 | 567 | 1748 |
| HD1-t2 | cTfh | P3_G5 | HLEKRIENLNKKVDD | 416 | 430 | 15 | 697 | 17982 |
| HD1-t2 | cTfh | P3_G6 | HLEKRIENLNKKVDD | 416 | 430 | 15 | 662 | 8158 |
| HD1-t2 | cTfh | P3_G8 | HLEKRIENLNKKVDD | 416 | 430 | 15 | 796 | 4640 |
| HD1-t2 | cTfh | P3_H2 | HLEKRIENLNKKVDD | 416 | 430 | 15 | 662 | 21611 |
| HD1-t2 | cTfh | P3_H4 | HLEKRIENLNKKVDD | 416 | 430 | 15 | 1153 | 14160 |
| HD1-t2 | cTfh | P3_H6 | HLEKRIENLNKKVDD | 416 | 430 | 15 | 432 | 1743 |
| HD1-t2 | cTfh | P3_H8 | HLEKRIENLNKKVDD | 416 | 430 | 15 | 1035 | 6409 |
| HD1-t2 | cTfh | P1_A4 | HLEKRIENLNKKVDD | 416 | 430 | 15 | 679 | 25835 |
| HD1-t2 | cTfh | P1_A6 | HLEKRIENLNKKVDD | 416 | 430 | 15 | 636 | 47018 |
| HD1-t2 | cTfh | P1_C1 | HLEKRIENLNKKVDD | 416 | 430 | 15 | 554 | 22117 |
| HD1-t2 | cTfh | P1_C3 | HLEKRIENLNKKVDD | 416 | 430 | 15 | 675 | 2371 |
| HD1-t2 | cTfh | P1_C11 | HLEKRIENLNKKVDD | 416 | 430 | 15 | 779 | 37658 |
| HD1-t2 | cTfh | P1_D9 | HLEKRIENLNKKVDD | 416 | 430 | 15 | 770 | 8599 |
| HD1-t2 | cTfh | P1_D12 | HLEKRIENLNKKVDD | 416 | 430 | 15 | 531 | 3470 |
| HD1-t2 | cTfh | P1_E2 | HLEKRIENLNKKVDD | 416 | 430 | 15 | 566 | 1872 |
| HD1-t2 | cTfh | P1_E3 | HLEKRIENLNKKVDD | 416 | 430 | 15 | 514 | 4496 |
| HD1-t2 | cTfh | P1_E7 | HLEKRIENLNKKVDD | 416 | 430 | 15 | 792 | 24727 |
| HD1-t2 | cTfh | P1_E9 | HLEKRIENLNKKVDD | 416 | 430 | 15 | 692 | 7909 |
| HD1-t2 | cTfh | P1_F1 | HLEKRIENLNKKVDD | 416 | 430 | 15 | 1356 | 19303 |
| HD1-t2 | cTfh | P1_F2 | HLEKRIENLNKKVDD | 416 | 430 | 15 | 521 | 1988 |
| HD1-t2 | cTfh | P1_F4 | HLEKRIENLNKKVDD | 416 | 430 | 15 | 632 | 5014 |
| HD1-t2 | cTfh | P1_F7 | HLEKRIENLNKKVDD | 416 | 430 | 15 | 1344 | 10526 |
| HD1-t2 | cTfh | P1_F10 | HLEKRIENLNKKVDD | 416 | 430 | 15 | 649 | 20758 |
| HD1-t2 | cTfh | P1_F11 | HLEKRIENLNKKVDD | 416 | 430 | 15 | 680 | 2873 |
| HD1-t2 | cTfh | P1_F12 | HLEKRIENLNKKVDD | 416 | 430 | 15 | 724 | 13223 |
| HD1-t2 | cTfh | P1_G1 | HLEKRIENLNKKVDD | 416 | 430 | 15 | 772 | 2277 |
| HD1-t2 | cTfh | P1_G6 | HLEKRIENLNKKVDD | 416 | 430 | 15 | 589 | 21166 |
| HD1-t2 | cTfh | P1_G9 | HLEKRIENLNKKVDD | 416 | 430 | 15 | 690 | 59730 |
| HD1-t2 | cTfh | P1_G10 | HLEKRIENLNKKVDD | 416 | 430 | 15 | 626 | 62687 |
| HD1-t2 | cTfh | P1_H2 | HLEKRIENLNKKVDD | 416 | 430 | 15 | 509 | 21180 |
| HD1-t2 | cTfh | P1_H4 | HLEKRIENLNKKVDD | 416 | 430 | 15 | 364 | 12485 |
| HD1-t2 | cTfh | P1_H5 | HLEKRIENLNKKVDD | 416 | 430 | 15 | 697 | 4826 |
| HD1-t2 | cTfh | P1_H6 | HLEKRIENLNKKVDD | 416 | 430 | 15 | 723 | 42107 |
| HD1-t2 | cTfh | P1_H8 | HLEKRIENLNKKVDD | 416 | 430 | 15 | 641 | 4145 |
| HD1-t2 | cTfh | P1_H9 | HLEKRIENLNKKVDD | 416 | 430 | 15 | 434 | 10398 |
| HD1-t2 | cTfh | P1_H10 | HLEKRIENLNKKVDD | 416 | 430 | 15 | 754 | 6232 |
| HD1-t2 | cTfh | P2_A4 | HLEKRIENLNKKVDD | 416 | 430 | 15 | 535 | 27800 |
| HD1-t2 | cTfh | P2_A8 | HLEKRIENLNKKVDD | 416 | 430 | 15 | 529 | 54851 |
| HD1-t2 | cTfh | P2_A9 | HLEKRIENLNKKVDD | 416 | 430 | 15 | 800 | 28103 |
| HD1-t2 | cTfh | P2_A10 | HLEKRIENLNKKVDD | 416 | 430 | 15 | 468 | 19765 |
| HD1-t2 | cTfh | P2_B4 | HLEKRIENLNKKVDD | 416 | 430 | 15 | 553 | 31324 |
| HD1-t2 | cTfh | P1_B3 | HLEKRIENLNKKVDD | 416 | 430 | 15 | 1593 | 6093 |
| HD1-t2 | cTfh | P1_B4 | HLEKRIENLNKKVDD | 416 | 430 | 15 | 1284 | 4691 |
| HD1-t2 | cTfh | P1_B5 | HLEKRIENLNKKVDD | 416 | 430 | 15 | 996 | 3010 |
| HD1-t2 | cTfh | P1_B10 | HLEKRIENLNKKVDD | 416 | 430 | 15 | 1027 | 19946 |
| HD1-t2 | cTfh | P1_B11 | HLEKRIENLNKKVDD | 416 | 430 | 15 | 1214 | 8141 |
| HD1-t2 | cTfh | P1_C2 | HLEKRIENLNKKVDD | 416 | 430 | 15 | 1302 | 5954 |
| HD1-t2 | cTfh | P1_C3 | HLEKRIENLNKKVDD | 416 | 430 | 15 | 886 | 2569 |
| HD1-t2 | cTfh | P1_C7 | HLEKRIENLNKKVDD | 416 | 430 | 15 | 1426 | 7808 |
| HD1-t2 | cTfh | P1_D2 | HLEKRIENLNKKVDD | 416 | 430 | 15 | 1428 | 5030 |
| HD1-t2 | cTfh | P1_D4 | HLEKRIENLNKKVDD | 416 | 430 | 15 | 696 | 2299 |
| HD1-t2 | cTfh | P1_D5 | HLEKRIENLNKKVDD | 416 | 430 | 15 | 1448 | 6387 |
| HD1-t2 | cTfh | P1_D7 | HLEKRIENLNKKVDD | 416 | 430 | 15 | 1330 | 16393 |
| HD1-t2 | cTfh | P1_E4 | HLEKRIENLNKKVDD | 416 | 430 | 15 | 1295 | 4201 |
| HD1-t2 | cTfh | P1_F3 | HLEKRIENLNKKVDD | 416 | 430 | 15 | 1299 | 22039 |
| HD1-t2 | cTfh | P1_G5 | HLEKRIENLNKKVDD | 416 | 430 | 15 | 999 | 72858 |
| HD1-t2 | cTfh | P2_A7 | HLEKRIENLNKKVDD | 416 | 430 | 15 | 892 | 2546 |
| HD1-t2 | cTfh | P2_B5 | HLEKRIENLNKKVDD | 416 | 430 | 15 | 870 | 45752 |
| HD1-t2 | cTfh | P2_D4 | HLEKRIENLNKKVDD | 416 | 430 | 15 | 727 | 2470 |
| HD1-t2 | cTfh | P2_D10 | HLEKRIENLNKKVDD | 416 | 430 | 15 | 723 | 3966 |
| HD1-t2 | cTfh | P2_F1 | HLEKRIENLNKKVDD | 416 | 430 | 15 | 731 | 3178 |
| HD1-t2 | cTfh | P2_F9 | HLEKRIENLNKKVDD | 416 | 430 | 15 | 1531 | 17466 |
| HD1-t2 | cTfh | P3_C11 | HLEKRIENLNKKVDD | 416 | 430 | 15 | 809 | 103047 |
| HD1-t2 | cTfh | P3_D6 | HLEKRIENLNKKVDD | 416 | 430 | 15 | 886 | 34388 |
| HD1-t2 | cTfh | P3_E2 | HLEKRIENLNKKVDD | 416 | 430 | 15 | 809 | 2500 |
| HD1-t2 | cTfh | P3_E4 | HLEKRIENLNKKVDD | 416 | 430 | 15 | 1239 | 21131 |
| HD1-t2 | cTfh | P3_E6 | HLEKRIENLNKKVDD | 416 | 430 | 15 | 685 | 23651 |
| HD1-t2 | cTfh | P3_G10 | HLEKRIENLNKKVDD | 416 | 430 | 15 | 1158 | 7250 |
| HD1-t2 | cTfh | P3_H4 | HLEKRIENLNKKVDD | 416 | 430 | 15 | 732 | 59705 |
| HD1-t2 | cTfh | P3_H5 | HLEKRIENLNKKVDD | 416 | 430 | 15 | 1085 | 6219 |
| HD1-t2 | cTfh | P3_H6 | HLEKRIENLNKKVDD | 416 | 430 | 15 | 916 | 19507 |
| HD1-t2 | cTfh | P3_H8 | HLEKRIENLNKKVDD | 416 | 430 | 15 | 723 | 13909 |
| HD1-t1 | Tcm | B8 | HLEKRIENLNKKVDD | 416 | 430 | 15 | 54 | 20572 |
| HD1-t1 | Tcm | C5 | HLEKRIENLNKKVDD | 416 | 430 | 15 | 9 | 971 |
| HD1-t1 | Tcm | C5 | HLEKRIENLNKKVDD | 416 | 430 | 15 | 22 | 3102 |
| HD1-t1 | Tcm | C3 | HLEKRIENLNKKVDD | 416 | 430 | 15 | 2130 | 7311 |
| HD1-t2 | Tcm | P1_A5 | HLEKRIENLNKKVDD | 416 | 430 | 15 | 696 | 22273 |
| HD1-t2 | Tcm | P1_A7 | HLEKRIENLNKKVDD | 416 | 430 | 15 | 592 | 17321 |
| HD1-t2 | Tcm | P1_A9 | HLEKRIENLNKKVDD | 416 | 430 | 15 | 479 | 25765 |
| HD1-t2 | Tcm | P1_A10 | HLEKRIENLNKKVDD | 416 | 430 | 15 | 736 | 4320 |
| HD1-t2 | Tcm | P1_B6 | HLEKRIENLNKKVDD | 416 | 430 | 15 | 814 | 17457 |
| HD1-t2 | Tcm | P1_B7 | HLEKRIENLNKKVDD | 416 | 430 | 15 | 510 | 11746 |
| HD1-t2 | Tcm | P1_B8 | HLEKRIENLNKKVDD | 416 | 430 | 15 | 549 | 9538 |
| HD1-t2 | Tcm | P1_B9 | HLEKRIENLNKKVDD | 416 | 430 | 15 | 705 | 9536 |
| HD1-t2 | Tcm | P1_C1 | HLEKRIENLNKKVDD | 416 | 430 | 15 | 610 | 12153 |
| HD1-t2 | Tcm | P1_C3 | HLEKRIENLNKKVDD | 416 | 430 | 15 | 692 | 16949 |
| HD1-t2 | Tcm | P1_C6 | HLEKRIENLNKKVDD | 416 | 430 | 15 | 684 | 7714 |
| HD1-t2 | Tcm | P1_C7 | HLEKRIENLNKKVDD | 416 | 430 | 15 | 503 | 10704 |
| HD1-t2 | Tcm | P1_C9 | HLEKRIENLNKKVDD | 416 | 430 | 15 | 602 | 9964 |
| HD1-t2 | Tcm | P1_C11 | HLEKRIENLNKKVDD | 416 | 430 | 15 | 701 | 31107 |
| HD1-t2 | Tcm | P1_D6 | HLEKRIENLNKKVDD | 416 | 430 | 15 | 513 | 46150 |
| HD1-t2 | Tcm | P1_D10 | HLEKRIENLNKKVDD | 416 | 430 | 15 | 592 | 5779 |
| HD1-t2 | Tcm | P1_E5 | HLEKRIENLNKKVDD | 416 | 430 | 15 | 561 | 6183 |
| HD1-t2 | Tcm | P1_E6 | HLEKRIENLNKKVDD | 416 | 430 | 15 | 474 | 3685 |
| HD1-t2 | Tcm | P1_E7 | HLEKRIENLNKKVDD | 416 | 430 | 15 | 478 | 4291 |
| HD1-t2 | Tcm | P1_E9 | HLEKRIENLNKKVDD | 416 | 430 | 15 | 503 | 13853 |
| HD1-t2 | Tcm | P1_F1 | HLEKRIENLNKKVDD | 416 | 430 | 15 | 632 | 8111 |
| HD1-t2 | Tcm | P1_F2 | HLEKRIENLNKKVDD | 416 | 430 | 15 | 538 | 37319 |
| HD1-t2 | Tcm | P1_F4 | HLEKRIENLNKKVDD | 416 | 430 | 15 | 772 | 13290 |
| HD1-t2 | Tcm | P1_F8 | HLEKRIENLNKKVDD | 416 | 430 | 15 | 486 | 6378 |
| HD1-t2 | Tcm | P1_F9 | HLEKRIENLNKKVDD | 416 | 430 | 15 | 658 | 6128 |
| HD1-t2 | Tcm | P1_G1 | HLEKRIENLNKKVDD | 416 | 430 | 15 | 447 | 14854 |
| HD1-t2 | Tcm | P1_G6 | HLEKRIENLNKKVDD | 416 | 430 | 15 | 561 | 22736 |
| HD1-t2 | Tcm | P1_G7 | HLEKRIENLNKKVDD | 416 | 430 | 15 | 533 | 10665 |
| HD1-t2 | Tcm | P1_G11 | HLEKRIENLNKKVDD | 416 | 430 | 15 | 749 | 3215 |
| HD1-t2 | Tcm | P1_G12 | HLEKRIENLNKKVDD | 416 | 430 | 15 | 525 | 17067 |
| HD1-t2 | Tcm | P1_H2 | HLEKRIENLNKKVDD | 416 | 430 | 15 | 529 | 14523 |
| HD1-t2 | Tcm | P1_H3 | HLEKRIENLNKKVDD | 416 | 430 | 15 | 649 | 43914 |
| HD1-t2 | Tcm | P1_H7 | HLEKRIENLNKKVDD | 416 | 430 | 15 | 518 | 2471 |
| HD1-t2 | Tcm | P1_H9 | HLEKRIENLNKKVDD | 416 | 430 | 15 | 613 | 21731 |
| HD1-t2 | Tcm | P2_A2 | HLEKRIENLNKKVDD | 416 | 430 | 15 | 566 | 4115 |
| HD1-t2 | Tcm | P2_A3 | HLEKRIENLNKKVDD | 416 | 430 | 15 | 610 | 3712 |
| HD1-t2 | Tcm | P2_A4 | HLEKRIENLNKKVDD | 416 | 430 | 15 | 702 | 4304 |
| HD1-t2 | Tcm | P2_A9 | HLEKRIENLNKKVDD | 416 | 430 | 15 | 469 | 10350 |
| HD1-t2 | Tcm | P2_B1 | HLEKRIENLNKKVDD | 416 | 430 | 15 | 568 | 8437 |
| HD1-t2 | Tcm | P2_B6 | HLEKRIENLNKKVDD | 416 | 430 | 15 | 535 | 20170 |
| HD1-t2 | Tcm | P2_B7 | HLEKRIENLNKKVDD | 416 | 430 | 15 | 535 | 3549 |
| HD1-t2 | Tcm | P2_B9 | HLEKRIENLNKKVDD | 416 | 430 | 15 | 816 | 7662 |
| HD1-t2 | Tcm | P2_D10 | HLEKRIENLNKKVDD | 416 | 430 | 15 | 1063 | 3651 |
| HD1-t2 | Tcm | P2_E6 | HLEKRIENLNKKVDD | 416 | 430 | 15 | 705 | 3001 |
| HD1-t2 | Tcm | P2_E10 | HLEKRIENLNKKVDD | 416 | 430 | 15 | 830 | 6882 |
| HD1-t2 | Tcm | P2_F2 | HLEKRIENLNKKVDD | 416 | 430 | 15 | 1179 | 22353 |
| HD1-t2 | Tcm | P2_F4 | HLEKRIENLNKKVDD | 416 | 430 | 15 | 1248 | 25969 |
| HD1-t2 | Tcm | P2_F5 | HLEKRIENLNKKVDD | 416 | 430 | 15 | 936 | 4872 |
| HD1-t2 | Tcm | P2_F12 | HLEKRIENLNKKVDD | 416 | 430 | 15 | 836 | 9370 |
| HD1-t2 | Tcm | P2_G1 | HLEKRIENLNKKVDD | 416 | 430 | 15 | 784 | 6850 |
| HD1-t2 | Tcm | P2_G7 | HLEKRIENLNKKVDD | 416 | 430 | 15 | 662 | 3717 |
| HD1-t2 | Tcm | P2_G10 | HLEKRIENLNKKVDD | 416 | 430 | 15 | 843 | 3189 |
| HD1-t2 | Tcm | P2_H6 | HLEKRIENLNKKVDD | 416 | 430 | 15 | 845 | 16522 |
| HD1-t2 | Tcm | P3_B7 | HLEKRIENLNKKVDD | 416 | 430 | 15 | 910 | 9052 |
| HD1-t2 | Tcm | P3_B8 | HLEKRIENLNKKVDD | 416 | 430 | 15 | 940 | 3402 |
| HD1-t2 | Tcm | P3_B9 | HLEKRIENLNKKVDD | 416 | 430 | 15 | 597 | 9496 |
| HD1-t2 | Tcm | P3_C5 | HLEKRIENLNKKVDD | 416 | 430 | 15 | 572 | 2782 |
| HD1-t2 | Tcm | P1_C4 | HLEKRIENLNKKVDD | 416 | 430 | 15 | 783 | 2272 |
| HD1-t2 | Tcm | P2_B4 | HLEKRIENLNKKVDD | 416 | 430 | 15 | 737 | 8482 |
| HD1-t2 | Tcm | P2_B10 | HLEKRIENLNKKVDD | 416 | 430 | 15 | 605 | 7337 |
| HD1-t2 | Tcm | P2_B12 | HLEKRIENLNKKVDD | 416 | 430 | 15 | 792 | 8661 |
| HD1-t2 | Tcm | P2_C6 | HLEKRIENLNKKVDD | 416 | 430 | 15 | 869 | 4114 |
| HD1-t2 | Tcm | P2_C11 | HLEKRIENLNKKVDD | 416 | 430 | 15 | 525 | 1958 |
| HD1-t2 | Tcm | P2_C12 | HLEKRIENLNKKVDD | 416 | 430 | 15 | 895 | 37463 |
| HD1-t2 | Tcm | P2_D9 | HLEKRIENLNKKVDD | 416 | 430 | 15 | 590 | 3942 |
| HD1-t2 | Tcm | P2_F1 | HLEKRIENLNKKVDD | 416 | 430 | 15 | 926 | 21726 |
| HD1-t2 | Tcm | P2_F7 | HLEKRIENLNKKVDD | 416 | 430 | 15 | 631 | 2259 |
| HD1-t2 | Tcm | P2_G6 | HLEKRIENLNKKVDD | 416 | 430 | 15 | 1480 | 5311 |
| HD1-t2 | Tcm | P3_B8 | HLEKRIENLNKKVDD | 416 | 430 | 15 | 770 | 12547 |
| HD1-t1 | Tem | B3 | HLEKRIENLNKKVDD | 416 | 430 | 15 | 57 | 3913 |
| HD1-t1 | Tem | F4 | HLEKRIENLNKKVDD | 416 | 430 | 15 | 48 | 6015 |
| HD1-t2 | Tem | P1_A9 | HLEKRIENLNKKVDD | 416 | 430 | 15 | 757 | 6386 |
| HD1-t2 | Tem | P1_A11 | HLEKRIENLNKKVDD | 416 | 430 | 15 | 705 | 36772 |
| HD1-t2 | Tem | P1_A12 | HLEKRIENLNKKVDD | 416 | 430 | 15 | 853 | 2501 |
| HD1-t2 | Tem | P1_C1 | HLEKRIENLNKKVDD | 416 | 430 | 15 | 684 | 4556 |
| HD1-t2 | Tem | P1_C3 | HLEKRIENLNKKVDD | 416 | 430 | 15 | 858 | 2466 |
| HD1-t2 | Tem | P1_C6 | HLEKRIENLNKKVDD | 416 | 430 | 15 | 1292 | 5707 |
| HD1-t2 | Tem | P1_C9 | HLEKRIENLNKKVDD | 416 | 430 | 15 | 614 | 2460 |
| HD1-t2 | Tem | P1_E12 | HLEKRIENLNKKVDD | 416 | 430 | 15 | 770 | 8641 |
| HD1-t2 | Tem | P1_F4 | HLEKRIENLNKKVDD | 416 | 430 | 15 | 588 | 2316 |
| HD1-t2 | Tem | P1_G1 | HLEKRIENLNKKVDD | 416 | 430 | 15 | 645 | 7054 |
| HD1-t2 | Tem | P1_G2 | HLEKRIENLNKKVDD | 416 | 430 | 15 | 921 | 4779 |
| HD1-t2 | Tem | P1_G3 | HLEKRIENLNKKVDD | 416 | 430 | 15 | 671 | 17904 |
| HD1-t2 | Tem | P1_H6 | HLEKRIENLNKKVDD | 416 | 430 | 15 | 653 | 4661 |
| HD1-t2 | Tem | P2_A1 | HLEKRIENLNKKVDD | 416 | 430 | 15 | 653 | 3303 |
| HD1-t2 | Tem | P2_A2 | HLEKRIENLNKKVDD | 416 | 430 | 15 | 894 | 5115 |
| HD1-t2 | Tem | P2_C3 | HLEKRIENLNKKVDD | 416 | 430 | 15 | 675 | 7089 |
| HD1-t2 | Tem | P2_D11 | HLEKRIENLNKKVDD | 416 | 430 | 15 | 542 | 3240 |
| HD1-t2 | Tem | P2_E10 | HLEKRIENLNKKVDD | 416 | 430 | 15 | 599 | 8805 |
| HD1-t2 | Tem | P2_F8 | HLEKRIENLNKKVDD | 416 | 430 | 15 | 564 | 3133 |
| HD1-t2 | Tem | P2_H3 | HLEKRIENLNKKVDD | 416 | 430 | 15 | 672 | 8814 |
| HD1-t2 | Tem | P2_H8 | HLEKRIENLNKKVDD | 416 | 430 | 15 | 631 | 2878 |
| HD1-t2 | Tem | P3_B4 | HLEKRIENLNKKVDD | 416 | 430 | 15 | 555 | 6973 |
| HD1-t2 | Tem | P1_E2 | HLEKRIENLNKKVDD | 416 | 430 | 15 | 626 | 4187 |
| HD1-t2 | Tem | P1_F7 | HLEKRIENLNKKVDD | 416 | 430 | 15 | 658 | 66991 |
| HD1-t2 | Tem | P1_G10 | HLEKRIENLNKKVDD | 416 | 430 | 15 | 766 | 3856 |
| HD1-t2 | Tem | P2_B4 | HLEKRIENLNKKVDD | 416 | 430 | 15 | 731 | 4028 |
| HD1-t2 | Tem | P2_B8 | HLEKRIENLNKKVDD | 416 | 430 | 15 | 1105 | 278741 |
| HD1-t2 | Tcm | P1_H6 | ELLVLLENERTLDYH | 441 | 455 | 15 | 5720 | 76062 |
| HD1-t2 | cTfh | P1_C5 | ELLVLLENERTLDYHDSNVK | 441 | 460 | 20 | 2630 | 12579 |
| HD1-t1 | Tcm | B5 | LENERTLDYHDSNVK | 446 | 460 | 15 | 67 | 38816 |
| HD1-t1 | Tcm | I4 | LENERTLDYHDSNVK | 446 | 460 | 15 | 17 | 13057 |
| HD1-t1 | Tcm | L3 | LENERTLDYHDSNVK | 446 | 460 | 15 | 9 | 6635 |
| HD1-t1 | Tcm | M11 | LENERTLDYHDSNVK | 446 | 460 | 15 | 23 | 8795 |
| HD1-t1 | Tcm | F8 | CFEFYHKCDNTCMES | 481 | 495 | 15 | 1576 | 10408 |
| HD1-t1 | Tcm | C6 | CFEFYHKCDNTCMES | 481 | 495 | 15 | 291 | 1631 |
| HD1-t1 | Tcm | M2 | CFEFYHKCDNTCMES | 481 | 495 | 15 | 323 | 3155 |
| HD1-t2 | cTfh | P2_B6 | KLNREEIDGVKLESTRIYQI | 511 | 530 | 20 | 1576 | 7823 |
| HD1-t2 | cTfh | P1_E5 | RIYQILAIYSTVASS | 526 | 540 | 15 | 4464 | 20392 |
| HD1-t1 | Tcm | B3 | RIYQILAIYSTVASS | 526 | 540 | 15 | 54 | 2331 |
| HD1-t1 | Tcm | B7 | RIYQILAIYSTVASS | 526 | 540 | 15 | 22 | 34735 |
| HD1-t1 | Tcm | E3 | RIYQILAIYSTVASS | 526 | 540 | 15 | 50 | 16289 |
| HD1-t1 | Tcm | H4 | RIYQILAIYSTVASS | 526 | 540 | 15 | 19 | 29613 |
| HD1-t1 | Tcm | I6 | RIYQILAIYSTVASS | 526 | 540 | 15 | 4 | 12824 |
| HD1-t1 | Tcm | M9 | RIYQILAIYSTVASS | 526 | 540 | 15 | 75 | 42415 |

Epitope mapping of 456 H1-HA-reactive T cell clones from donor HD1 was performed by screening with overlapping peptides spanning the entire H1-HA sequence in the presence of autologous APCs. Proliferation was assessed on day 3 after a 16-h pulse with [^3^H]-thymidine and expressed as Counts per minute (Cpm). For each T cell clone the subset of origin, the start and end position of the epitope residues in H1-HA, and the Cpm values after stimulation with autologous APCs untreated or pulsed with the indicated HA peptide are reported.
