# Supplementary material for: Deciphering and predicting CD4+ T cell immunodominance of influenza virus hemagglutinin
Source: J Exp Med. 2020 Jul 9;217(10):e20200206. doi: 10.1084/jem.20200206 (PMC7537397; doi:10.1084/jem.20200206)
Supplement: Table S2 — shows TCR Vβ sequence and epitope specificity of H1-HA–reactive T cell clones isolated from CD4+ memory (Tcm, Tem, or cTfh) T cell compartment. [file JEM_20200206_TableS2.docx]

**Table S2.** TCR-Vβ sequence and epitope specificity of H1-HA–reactive T cell clones isolated from CD4^+^ memory (Tcm, Tem, or cTfh) T cell compartment

| **Subset** | **ID** | **Vβ gene** | **Jβ gene** | **Vβ CDR3** | **Epitope** | **Start** | **Stop** | **Length** | **N sister clones** | | | |
| --- | --- | --- | --- | --- | --- | --- | --- | --- | --- | --- | --- | --- |
|  |  |  |  |  |  |  |  |  | tot | from Tcm | from Tem | from cTfh |
| Memory | mem_1 | TRBV20-1 | TRBJ2-5 | CSASPGRGETQYF | TFATANADTLCIGYH | 11 | 25 | 15 | 1 |  | 1 |  |
| Memory | mem_2 | TRBV12-5 | TRBJ1-5 | CASGINSNHPQHF | PLHLGKCNIAGWILG | 66 | 80 | 15 | 1 |  | 1 |  |
| Memory | mem_3 | TRBV11-3 | TRBJ2-5 | CASSPGGETQYF | KGKEVLVLWGIHHPS | 186 | 200 | 15 | 2 | 1 |  | 1 |
| Memory | mem_4 | TRBV6-1 | TRBJ2-5 | CASSISGTGFQETQYF | KGKEVLVLWGIHHPS | 186 | 200 | 15 | 5 | 5 |  |  |
| Memory | mem_5 | TRBV18 | TRBJ2-6 | CASSPSAAGANVLTF | KGKEVLVLWGIHHPS | 186 | 200 | 15 | 6 | 6 |  |  |
| Memory | mem_6 | TRBV6-5 | TRBJ1-5 | CASSYSSFLREPQHF | KGKEVLVLWGIHHPS | 186 | 200 | 15 | 1 | 1 |  |  |
| Memory | mem_7 | TRBV12-4 | TRBJ1-3 | CASTRESWRRNTIYF | KGKEVLVLWGIHHPS | 186 | 200 | 15 | 2 | 1 | 1 |  |
| Memory | mem_8 | TRBV11-3 | TRBJ2-3 | CASRTGADTQYF | KGKEVLVLWGIHHPSTSADQ | 186 | 205 | 20 | 1 | 1 |  |  |
| Memory | mem_9 | TRBV12-4 | TRBJ2-6 | CASRRDSGTGANVLTF | KFKPEIAIRPKVRDQ | 226 | 240 | 15 | 2 | 2 |  |  |
| Memory | mem_10 | TRBV11-2 | TRBJ2-7 | CASSLAPGQGLYEQYF | KFKPEIAIRPKVRDQ | 226 | 240 | 15 | 1 | 1 |  |  |
| Memory | mem_11 | TRBV5-1 | TRBJ2-2 | CASSLGPQSKNTGELFF | EGRMNYYWTLVEPGD | 241 | 255 | 15 | 1 |  | 1 |  |
| Memory | mem_12 | TRBV11-2 | TRBJ1-6 | CASSLQGAGSSPLHF | EGRMNYYWTLVEPGD | 241 | 255 | 15 | 2 |  | 1 | 1 |
| Memory | mem_13 | TRBV7-2 | TRBJ2-1 | CASSLWRRGSYNEQFF | EGRMNYYWTLVEPGD | 241 | 255 | 15 | 1 | 1 |  |  |
| Memory | mem_14 | TRBV6-4 | TRBJ2-1 | CSSGEQGAGGNEQFF | EGRMNYYWTLVEPGD | 241 | 255 | 15 | 1 |  |  | 1 |
| Memory | mem_15 | TRBV7-9 | TRBJ1-3 | CASSPGTESSGNTIYF | EGRMNYYWTLVEPGDKITFE | 241 | 260 | 20 | 2 |  | 2 |  |
| Memory | mem_16 | TRBV6-4 | TRBJ2-6 | CASSPQGCGANVLTF | EGRMNYYWTLVEPGDKITFE | 241 | 260 | 20 | 1 |  |  | 1 |
| Memory | mem_17 | TRBV6-4 | TRBJ2-2 | CASSQAGNSGELFF | EGRMNYYWTLVEPGDKITFE | 241 | 260 | 20 | 1 |  | 1 |  |
| Memory | mem_18 | TRBV6-4 | TRBJ2-2 | CASSQAGNTGELFF | EGRMNYYWTLVEPGDKITFE | 241 | 260 | 20 | 1 |  | 1 |  |
| Memory | mem_19 | TRBV12-3 | TRBJ2-6 | CASSQQGSGANVLTF | EGRMNYYWTLVEPGDKITFE | 241 | 260 | 20 | 2 | 2 |  |  |
| Memory | mem_20 | TRBV6-4 | TRBJ2-3 | CASSQQGTGADTQYF | EGRMNYYWTLVEPGDKITFE | 241 | 260 | 20 | 1 | 1 |  |  |
| Memory | mem_21 | TRBV5-5 | TRBJ2-7 | CASSSTVPSYEQYF | EGRMNYYWTLVEPGDKITFE | 241 | 260 | 20 | 2 | 2 |  |  |
| Memory | mem_22 | TRBV19 | TRBJ2-7 | CASSTNLGDYEQYF | YYWTLVEPGDKITFE | 246 | 260 | 15 | 1 | 1 |  |  |
| Memory | mem_23 | TRBV2 | TRBJ1-5 | CASSVGQGVSQPQHF | VVPRYAFAMERNAGS | 266 | 280 | 15 | 1 | 1 |  |  |
| Memory | mem_24 | TRBV6-1 | TRBJ2-1 | CASSESTSGTFNEQFF | QNAIDEITNKVNSVI | 386 | 400 | 15 | 1 | 1 |  |  |
| Memory | mem_25 | TRBV18 | TRBJ1-3 | CASSPLQGISGRPRSF | VNSVIEKMNTQFTAV | 396 | 410 | 15 | 1 | 1 |  |  |
| Memory | mem_26 | TRBV6-5 | TRBJ2-5 | CASSTGDTGGRQETQYF | VNSVIEKMNTQFTAV | 396 | 410 | 15 | 1 |  |  | 1 |
| Memory | mem_27 | TRBV28 | TRBJ1-5 | CASRRKSVGQVGQPQHF | EKMNTQFTAVGKEFN | 401 | 415 | 15 | 1 |  |  | 1 |
| Memory | mem_28 | TRBV10-2 | TRBJ2-3 | CASSELGQATDTQYF | EKMNTQFTAVGKEFNHLEKR | 401 | 420 | 20 | 10 |  | 10 |  |
| Memory | mem_29 | TRBV11-1 | TRBJ2-7 | CASSQVRGAQTYEQYF | EKMNTQFTAVGKEFNHLEKR | 401 | 420 | 20 | 1 |  |  | 1 |
| Memory | mem_30 | TRBV12-4 | TRBJ1-6 | CASSFPPPIHF | EKMNTQFTAVGKEFNHLEKR | 401 | 420 | 20 | 2 |  |  | 2 |
| Memory | mem_31 | TRBV14 | TRBJ1-2 | CASSQVVSGYGYTF | EKMNTQFTAVGKEFNHLEKR | 401 | 420 | 20 | 8 |  |  | 8 |
| Memory | mem_32 | TRBV14 | TRBJ1-2 | CASSQLSGAYGHFF | EKMNTQFTAVGKEFNHLEKR | 401 | 420 | 20 | 1 |  |  | 1 |
| Memory | mem_33 | TRBV14 | TRBJ1-2 | CASSQVNSAYGHTF | EKMNTQFTAVGKEFNHLEKR | 401 | 420 | 20 | 1 |  |  | 1 |
| Memory | mem_34 | TRBV20-1 | TRBJ1-2 | CSASTPGGIYGYTF | EKMNTQFTAVGKEFNHLEKR | 401 | 420 | 20 | 10 |  |  | 10 |
| Memory | mem_35 | TRBV20-1 | TRBJ1-5 | CSLDRPNQPQHF | EKMNTQFTAVGKEFNHLEKR | 401 | 420 | 20 | 6 |  |  | 6 |
| Memory | mem_36 | TRBV20-1 | TRBJ2-3 | CSARVGGSSDTQYF | EKMNTQFTAVGKEFNHLEKR | 401 | 420 | 20 | 1 |  | 1 |  |
| Memory | mem_37 | TRBV24-1 | TRBJ2-2 | CATLGMGTGELFF | EKMNTQFTAVGKEFNHLEKR | 401 | 420 | 20 | 3 | 3 |  |  |
| Memory | mem_38 | TRBV24-1 | TRBJ2-3 | CATQGMGTDTQYF | EKMNTQFTAVGKEFNHLEKR | 401 | 420 | 20 | 2 |  |  | 2 |
| Memory | mem_39 | TRBV27 | TRBJ2-6 | CASRSGGSGANVLTF | EKMNTQFTAVGKEFNHLEKR | 401 | 420 | 20 | 4 |  | 4 |  |
| Memory | mem_40 | TRBV3-1 | TRBJ2-1 | CASSPSTSGGSQFF | EKMNTQFTAVGKEFNHLEKR | 401 | 420 | 20 | 10 |  | 10 |  |
| Memory | mem_41 | TRBV7-8 | TRBJ2-2 | CASTQAAGLNTGELFF | EKMNTQFTAVGKEFNHLEKR | 401 | 420 | 20 | 1 | 1 |  |  |
| Memory | mem_42 | TRBV6-6 | TRBJ1-4 | CASTPAANEKLFF | QFTAVGKEFNHLEKR | 406 | 420 | 15 | 1 |  |  | 1 |
| Memory | mem_43 | TRBV7-9 | TRBJ2-4 | CASSVTSGGEVKNIQYF | GKEFNHLEKRIENLN | 411 | 425 | 15 | 14 |  | 14 |  |
| Memory | mem_44 | TRBV7-9 | TRBJ2-4 | CASSVTSGGEVKNVQYF | GKEFNHLEKRIENLN | 411 | 425 | 15 | 2 |  |  | 2 |
| Memory | mem_45 | TRBV7-9 | TRBJ1-1 | CGAAAYPEGGEIKNKQFF | GKEFNHLEKRIENLN | 411 | 425 | 15 | 1 |  | 1 |  |
| Memory | mem_46 | TRBV12-4 | TRBJ1-1 | CASSFTMNTEAFF | HLEKRIENLNKKVDD | 416 | 430 | 15 | 5 | 5 |  |  |
| Memory | mem_47 | TRBV12-4 | TRBJ1-1 | CASSFTMYTKAFF | HLEKRIENLNKKVDD | 416 | 430 | 15 | 1 | 1 |  |  |
| Memory | mem_48 | TRBV19 | TRBJ2-7 | CASSTASGRSSYEQYF | HLEKRIENLNKKVDD | 416 | 430 | 15 | 19 |  |  | 19 |
| Memory | mem_49 | TRBV19 | TRBJ1-2 | CASSSEGGGRANGYTF | HLEKRIENLNKKVDD | 416 | 430 | 15 | 16 |  |  | 16 |
| Memory | mem_50 | TRBV19 | TRBJ1-5 | CAIGQGGSNQPQHF | HLEKRIENLNKKVDD | 416 | 430 | 15 | 11 |  |  | 11 |
| Memory | mem_51 | TRBV19 | TRBJ2-3 | CASSPSSGSRDTQYF | HLEKRIENLNKKVDD | 416 | 430 | 15 | 9 |  | 4 | 5 |
| Memory | mem_52 | TRBV19 | TRBJ1-5 | CASSSEGGGRQPQHF | HLEKRIENLNKKVDD | 416 | 430 | 15 | 9 |  | 7 | 2 |
| Memory | mem_53 | TRBV19 | TRBJ2-5 | CASQGTSGGRETQYF | HLEKRIENLNKKVDD | 416 | 430 | 15 | 8 | 1 | 1 | 6 |
| Memory | mem_54 | TRBV19 | TRBJ1-1 | CASSEVRGGRTEAFF | HLEKRIENLNKKVDD | 416 | 430 | 15 | 8 | 5 | 3 |  |
| Memory | mem_55 | TRBV19 | TRBJ1-5 | CATGQGGSNQPQHF | HLEKRIENLNKKVDD | 416 | 430 | 15 | 6 |  | 1 | 5 |
| Memory | mem_56 | TRBV19 | TRBJ1-5 | CASGQGGSNQPQHF | HLEKRIENLNKKVDD | 416 | 430 | 15 | 4 |  |  | 4 |
| Memory | mem_57 | TRBV19 | TRBJ1-5 | CASTSETGGRQPQHF | HLEKRIENLNKKVDD | 416 | 430 | 15 | 4 |  |  | 4 |
| Memory | mem_58 | TRBV19 | TRBJ1-2 | CASKDVAGTAKDGYTF | HLEKRIENLNKKVDD | 416 | 430 | 15 | 2 |  | 1 | 1 |
| Memory | mem_59 | TRBV19 | TRBJ1-5 | CASSAEFQGRQPQHF | HLEKRIENLNKKVDD | 416 | 430 | 15 | 2 |  | 1 | 1 |
| Memory | mem_60 | TRBV19 | TRBJ2-5 | CASSPQSGKETQYF | HLEKRIENLNKKVDD | 416 | 430 | 15 | 2 | 1 | 1 |  |
| Memory | mem_61 | TRBV19 | TRBJ1-2 | CASSSEGGGRVNGYTF | HLEKRIENLNKKVDD | 416 | 430 | 15 | 2 |  |  | 2 |
| Memory | mem_62 | TRBV19 | TRBJ1-1 | CASNEVRGGNHQPLF | HLEKRIENLNKKVDD | 416 | 430 | 15 | 1 | 1 |  |  |
| Memory | mem_63 | TRBV19 | TRBJ1-2 | CASRSTGGGLEGYTF | HLEKRIENLNKKVDD | 416 | 430 | 15 | 1 |  |  | 1 |
| Memory | mem_64 | TRBV19 | TRBJ1-5 | CASSADTGGRQPQHF | HLEKRIENLNKKVDD | 416 | 430 | 15 | 1 |  |  | 1 |
| Memory | mem_65 | TRBV19 | TRBJ1-1 | CASSEVRGGLTEAFF | HLEKRIENLNKKVDD | 416 | 430 | 15 | 1 | 1 |  |  |
| Memory | mem_66 | TRBV19 | TRBJ2-5 | CASSSAGKETQYF | HLEKRIENLNKKVDD | 416 | 430 | 15 | 1 |  |  | 1 |
| Memory | mem_67 | TRBV19 | TRBJ1-2 | CASSSEGQGRGGYTF | HLEKRIENLNKKVDD | 416 | 430 | 15 | 1 |  | 1 |  |
| Memory | mem_68 | TRBV19 | TRBJ2-7 | CASSTASGRFSYEQYF | HLEKRIENLNKKVDD | 416 | 430 | 15 | 1 |  |  | 1 |
| Memory | mem_69 | TRBV19 | TRBJ2-7 | CASSTASGRSFFEQYF | HLEKRIENLNKKVDD | 416 | 430 | 15 | 1 |  |  | 1 |
| Memory | mem_70 | TRBV19 | TRBJ2-7 | CASSTASGRSFYEQYF | HLEKRIENLNKKVDD | 416 | 430 | 15 | 1 |  |  | 1 |
| Memory | mem_71 | TRBV19 | TRBJ2-7 | CASSTASGRSLYEQYF | HLEKRIENLNKKVDD | 416 | 430 | 15 | 1 |  |  | 1 |
| Memory | mem_72 | TRBV19 | TRBJ2-7 | CASVPPAGGHSYEQYF | HLEKRIENLNKKVDD | 416 | 430 | 15 | 1 |  |  | 1 |
| Memory | mem_73 | TRBV19 | TRBJ1-2 | CHSSSEGQGRGGHTF | HLEKRIENLNKKVDD | 416 | 430 | 15 | 1 |  | 1 |  |
| Memory | mem_74 | TRBV20-1 | TRBJ2-2 | CSARDGSWTGELFF | HLEKRIENLNKKVDD | 416 | 430 | 15 | 10 | 10 |  |  |
| Memory | mem_75 | TRBV20-1 | TRBJ2-2 | CSARDGSWSGELFF | HLEKRIENLNKKVDD | 416 | 430 | 15 | 1 | 1 |  |  |
| Memory | mem_76 | TRBV20-1 | TRBJ2-2 | CSGRDGSWNGELFF | HLEKRIENLNKKVDD | 416 | 430 | 15 | 1 | 1 |  |  |
| Memory | mem_77 | TRBV3-1 | TRBJ1-6 | CASSQGTPGSPLHF | HLEKRIENLNKKVDD | 416 | 430 | 15 | 2 |  | 2 |  |
| Memory | mem_78 | TRBV3-1 | TRBJ2-5 | CASSESSGRETQYF | HLEKRIENLNKKVDD | 416 | 430 | 15 | 1 |  |  | 1 |
| Memory | mem_79 | TRBV3-1 | TRBJ2-3 | CASSSQGSRTDTQYF | HLEKRIENLNKKVDD | 416 | 430 | 15 | 1 |  | 1 |  |
| Memory | mem_80 | TRBV6-6 | TRBJ1-5 | CASSYSSANTGGNQPQHF | HLEKRIENLNKKVDD | 416 | 430 | 15 | 1 |  |  | 1 |
| Memory | mem_81 | TRBV7-2 | TRBJ1-4 | CAAGTAKEKLFF | HLEKRIENLNKKVDD | 416 | 430 | 15 | 3 | 2 |  | 1 |
| Memory | mem_82 | TRBV10-2 | TRBJ2-1 | CSSRPGEGGNNEQFF | ELLVLLENERTLDYH | 441 | 455 | 15 | 1 | 1 |  |  |
| Memory | mem_83 | TRBV11-2 | TRBJ2-7 | CASSPRTDYEQYF | LENERTLDYHDSNVK | 446 | 460 | 15 | 3 | 3 |  |  |
| Memory | mem_84 | TRBV30 | TRBJ2-2 | CAWSGDSYTGELFF | LENERTLDYHDSNVK | 446 | 460 | 15 | 2 | 2 |  |  |
| Memory | mem_85 | TRBV11-3 | TRBJ2-1 | CASSLDPTNNEQFF | CFEFYHKCDNTCMES | 481 | 495 | 15 | 2 | 2 |  |  |
| Memory | mem_86 | TRBV6-2 | TRBJ2-2 | CASSYREVGDRGPVF | CFEFYHKCDNTCMES | 481 | 495 | 15 | 1 | 1 |  |  |
| Memory | mem_87 | TRBV5-5 | TRBJ2-7 | CASSLNGLSYEQYF | RIYQILAIYSTVASS | 526 | 540 | 15 | 1 | 1 |  |  |
| Memory | mem_88 | TRBV9 | TRBJ2-7 | CASSVAAQAYEQYF | RIYQILAIYSTVASS | 526 | 540 | 15 | 3 | 3 |  |  |
|  |  |  |  | Clonotypes (tot): 88 |  |  |  |  | Clones (tot): 274 | | | |
