# Supplementary material for: Deciphering and predicting CD4+ T cell immunodominance of influenza virus hemagglutinin
Source: J Exp Med. 2020 Jul 9;217(10):e20200206. doi: 10.1084/jem.20200206 (PMC7537397; doi:10.1084/jem.20200206)
Supplement: Table S3 — shows HLA class II typing of the four HDs included in this study. [file JEM_20200206_TableS3.docx]

**Table S3.** HLA class II typing of the four healthy donors included in this study

|  |  | **Gender** | **Age** |  | **HLA-DRB1*** | |  | **HLA-DQA1*** | |  | **HLA-DQB1*** | |  | **HLA-DPA1*** | |  | **HLA-DPB1*** | |  |
| --- | --- | --- | --- | --- | --- | --- | --- | --- | --- | --- | --- | --- | --- | --- | --- | --- | --- | --- | --- |
| **HD1** |  | Male | 50 |  | 01:01 | 08:01 |  | 01:01 | 04:01 |  | 04:02 | 05:01 |  | 01:03 | 02:01 |  | 04:02 | 13:01 |  |
| **HD2** |  | Female | 60 |  | 07:01 |  |  | 02:01 | 02:01 |  | 02:02 | 02:02 |  | 02:01 | 02:01 |  | 11:01 | 17:01 |  |
| **HD3** |  | Male | 19 |  | 03:01 | 04:08 |  | 03:03 | 05:01 |  | 02:01 | 03:01 |  | 01:03 | 02:02/ 02:06 |  | 04:01 | 05:01 |  |
| **HD4** |  | Female | 41 |  | 04:04 | 07:01 |  | 02:01 | 03:01 |  | 03:02 | 03:03 |  | 01:03 | 01:03 |  | 04:01 | 03:01/ 104:01 |  |
