# Supplementary material for: Deciphering and predicting CD4+ T cell immunodominance of influenza virus hemagglutinin
Source: J Exp Med. 2020 Jul 9;217(10):e20200206. doi: 10.1084/jem.20200206 (PMC7537397; doi:10.1084/jem.20200206)
Supplement: Table S4 — shows TCR Vβ sequence and epitope specificity of H1-HA–reactive T cell clones isolated from the CD4+ naive T cell compartment. [file JEM_20200206_TableS4.docx]

**Table S4.** TCR-Vβ sequence and epitope specificity of H1-HA–reactive T cell clones isolated from the CD4^+^ naive T cell compartment.

| **Subset** | **ID** | **Vβ gene** | **Jβ gene** | **Vβ CDR3** | **Epitope** | **Start^1^** | **Stop^1^** | **Length** | **N sister clones^2^** |
| --- | --- | --- | --- | --- | --- | --- | --- | --- | --- |
| Naive | nai_1 | TRBV9 | TRBJ2-5 | CASSLQGQETQYF | MKAILVVLLYTFATANADTL | 1 | 20 | 20 | 1 |
| Naive | nai_2 | TRBV11-2 | TRBJ2-3 | CASSTGTGPDTQYF | EDKHNGKLCKLRGVAPLHLG | 51 | 70 | 20 | 1 |
| Naive | nai_3 | TRBV30 | TRBJ1-1 | CAWTPPQSTGDTEAFF | EDKHNGKLCKLRGVAPLHLG | 51 | 70 | 20 | 1 |
| Naive | nai_4 | TRBV11-1 | TRBJ2-7 | CASSLKDIYEQYF | GKLCKLRGVAPLHLG | 56 | 70 | 15 | 1 |
| Naive | nai_5 | TRBV11-2 | TRBJ1-5 | CASSSDSNQPQHF | GKLCKLRGVAPLHLG | 56 | 70 | 15 | 1 |
| Naive | nai_6 | TRBV9 | TRBJ2-1 | CASSVGDYLGYNEQFF | GKLCKLRGVAPLHLG | 56 | 70 | 15 | 1 |
| Naive | nai_7 | TRBV20-1 | TRBJ1-5 | CSATQGINQPQHF | PLHLGKCNIAGWILG | 66 | 80 | 15 | 1 |
| Naive | nai_8 | TRBV6-6 | TRBJ1-1 | CASSYQMRGTEAFF | SSFERFEIFPKTSSW | 126 | 140 | 15 | 1 |
| Naive | nai_9 | TRBV14 | not resolved | not resolved | NKGVTAACPHAGAKS | 146 | 160 | 15 | 1 |
| Naive | nai_10 | TRBV20-1 | TRBJ2-6 | CSATQSCGANVQTF | AGAKSFYKNLIWLVK | 156 | 170 | 15 | 1 |
| Naive | nai_11 | TRBV11-2 | TRBJ2-1 | CASSLDRGIEQFF | AGAKSFYKNLIWLVKKGNSY | 156 | 175 | 20 | 1 |
| Naive | nai_12 | TRBV5-1 | TRBJ2-3 | CASSLENNRPGTMDTQYF | AGAKSFYKNLIWLVKKGNSY | 156 | 175 | 20 | 1 |
| Naive | nai_13 | TRBV11-3 | TRBJ2-1 | CASSLSIWQGAYNEQFF | SRYSKKFKPEIAIRP | 221 | 235 | 15 | 1 |
| Naive | nai_14 | TRBV20-1 | TRBJ2-7 | CSAPTGTSPYEQYF | SRYSKKFKPEIAIRPKVRDQ | 221 | 240 | 20 | 1 |
| Naive | nai_15 | TRBV20-1 | TRBJ2-1 | CSARDPSGSVNEQLF | SRYSKKFKPEIAIRPKVRDQ | 221 | 240 | 20 | 1 |
| Naive | nai_16 | TRBV12-3 | TRBJ2-2 | CASSYSGGGNTGELFF | KFKPEIAIRPKVRDQ | 226 | 240 | 15 | 1 |
| Naive | nai_17 | TRBV5-1 | TRBJ1-1 | CASSLQGENTEAFF | EGRMNYYWTLVEPGD | 241 | 255 | 15 | 1 |
| Naive | nai_18 | TRBV5-1 | TRBJ1-1 | CASRQGMNTEAFF | EGRMNYYWTLVEPGDKITFE | 241 | 260 | 20 | 1 |
| Naive | nai_19 | TRBV20-1 | TRBJ2-1 | CSARDPSGSVNEQFF | ATGNLVVPRYAFAMERNAGS | 261 | 280 | 20 | 1 |
| Naive | nai_20 | TRBV3-1 | TRBJ2-2 | CATKPGGTGELFF | VVPRYAFAMERNAGS | 266 | 280 | 15 | 1 |
| Naive | nai_21 | TRBV10-3 | TRBJ1-4 | CAISEMATNEKLFF | VVPRYAFAMERNAGSGIIIS | 266 | 285 | 20 | 2 |
| Naive | nai_22 | TRBV6-6 | TRBJ1-3 | CASSRQGGSGNTIYF | GAINTSLPFQNIHPITIGKC | 301 | 320 | 20 | 2 |
| Naive | nai_23 | TRBV7-8 | TRBJ1-1 | not resolved | GAINTSLPFQNIHPITIGKC | 301 | 320 | 20 | 1 |
| Naive | nai_24 | TRBV10-1 | TRBJ2-1 | CASSTPGVGNEQFF | SLPFQNIHPITIGKC | 306 | 320 | 15 | 2 |
| Naive | nai_25 | TRBV7-8 | TRBJ2-7 | CASSLLTSGGNGQYF | EKMNTQFTAVGKEFNHLEKR | 401 | 420 | 20 | 1 |
| Naive | nai_26 | TRBV11-2 | TRBJ1-3 | CASSYGAPTSDTIYF | GFLDIWTYNAELLVL | 431 | 445 | 15 | 1 |
| Naive | nai_27 | TRBV11-2 | TRBJ1-1 | CASSNRVNTEAFF | GFLDIWTYNAELLVLLENER | 431 | 450 | 20 | 1 |
| Naive | nai_28 | TRBV12-4 | TRBJ2-2 | not resolved | GFLDIWTYNAELLVLLENER | 431 | 450 | 20 | 1 |
| Naive | nai_29 | TRBV5-1 | TRBJ1-2 | CASTPRYEGAKYGYTF | GFLDIWTYNAELLVLLENER | 431 | 450 | 20 | 1 |
| Naive | nai_30 | TRBV6-5 | TRBJ1-6 | CASSYSGRGESPLHF | LENERTLDYHDSNVK | 446 | 460 | 15 | 1 |
| Naive | nai_31 | TRBV6-5 | TRBJ2-5 | CASSYLGETQYF | EIGNGCFEFYHKCDN | 476 | 490 | 15 | 1 |
| Naive | nai_32 | TRBV5-5 | TRBJ1-5 | CASSFLALGQPQHF | KLESTRIYQILAIYSTVASS | 521 | 540 | 20 | 1 |
| Naive | nai_33 | TRBV20-1 | TRBJ1-1 | CSARKTGEVWTEAFF | TVASSLVLVVSLGAI | 536 | 550 | 15 | 1 |
| Naive | nai_34 | TRBV14 | TRBJ1-6 | CASSQGTGSPLHF | TVASSLVLVVSLGAISFWMC | 536 | 555 | 20 | 1 |
| Naive | nai_35 | TRBV20-1 | TRBJ2-7 | CSALTSGSGEQYF | TVASSLVLVVSLGAISFWMC | 536 | 555 | 20 | 1 |
| Naive | nai_36 | TRBV6-2 | TRBJ2-2 | CASSSTSGRPGELFF | LVLVVSLGAISFWMC | 541 | 555 | 15 | 1 |
|  |  |  |  | Clonotypes (tot): 36 |  |  |  |  | Clones (tot): 39 |

1. Start and end position of the epitope residues in H1-HA A/California/07/2009 (H1N1).
2. Number of sister clones isolated from each subset.
