# Supplementary material for: Deciphering and predicting CD4+ T cell immunodominance of influenza virus hemagglutinin
Source: J Exp Med. 2020 Jul 9;217(10):e20200206. doi: 10.1084/jem.20200206 (PMC7537397; doi:10.1084/jem.20200206)
Supplement: Table S5 — lists H1-HA peptides identified by MS-based MHC-II peptidomics in donor HD1. [file JEM_20200206_TableS5.docx]

**Table S5.** H1-HA peptides identified by MS-based MHC-II peptidomics in donor HD1

| **Peptide sequence** | **Start** | **End** | **Length** | **Identification** (donor-cell type-replicate) | | |  | **HD1** |  |  |  |
| --- | --- | --- | --- | --- | --- | --- | --- | --- | --- | --- | --- |
|  |  |  |  | HD1-moDC-rep1 | HD1-moDC-rep2 | HD1-EBV-B(anti-head)-rep1 | HD1-EBV-B(anti-head)-rep2 | HD1-EBV-B(anti-head)-rep3 | HD1-EBV-B(anti-stem)-rep1 | HD1-EBV-B(anti-stem)-rep2 | HD1-EBV-B(anti-stem)-rep3 |
| YPGDFIDYEELREQ | 108 | 121 | 14 |  |  |  |  | + |  |  |  |
| IFPKTSSWPNHDSN | 133 | 146 | 14 |  | + |  |  |  |  |  |  |
| IFPKTSSWPNHDSNK | 133 | 147 | 15 | + | + |  |  |  |  |  |  |
| IFPKTSSWPNHDSNKG | 133 | 148 | 16 |  | + |  |  |  |  |  |  |
| FPKTSSWPNHDSNKG | 134 | 148 | 15 |  | + |  |  |  |  |  |  |
| YKNLIWLVKKGNSYPK | 162 | 177 | 16 |  |  | + |  | + |  |  |  |
| KNLIWLVKKGNSYPK | 163 | 177 | 15 |  |  | + |  | + |  |  |  |
| MNYYWTLVEPGDK | 244 | 256 | 13 |  |  | + | + |  |  |  |  |
| MNYYWTLVEPGDKIT | 244 | 258 | 15 |  |  | + |  |  |  |  |  |
| YWTLVEPGDK | 247 | 256 | 10 |  |  |  |  | + |  |  |  |
| VEPGDKITFEATGNLVVPRYA | 251 | 271 | 21 |  |  |  | + |  |  |  |  |
| EPGDKITFEATGNLVVPRY | 252 | 270 | 19 |  |  |  | + |  |  |  |  |
| EPGDKITFEATGNLVVPRYA | 252 | 271 | 20 |  |  |  | + | + |  |  |  |
| VVPRYAFAMERNAGSG | 266 | 281 | 16 |  | + |  |  |  |  |  |  |
| VPRYAFAMERNAGSG | 267 | 281 | 15 |  | + |  |  |  |  |  |  |
| AIDEITNKVNSVIE | 388 | 401 | 14 |  | + | + | + | + |  | + |  |
| AIDEITNKVNSVIEK | 388 | 402 | 15 |  | + | + |  | + |  |  |  |
| IDEITNKVNSVIE | 389 | 401 | 13 |  | + | + | + |  | + |  |  |
| IDEITNKVNSVIEK | 389 | 402 | 14 | + | + | + | + | + |  | + | + |
| DEITNKVNSVIE | 390 | 401 | 12 |  |  |  |  | + |  | + | + |
| DEITNKVNSVIEK | 390 | 402 | 13 | + | + |  |  |  |  | + | + |
| EITNKVNSVIE | 391 | 401 | 11 |  | + |  |  |  |  | + |  |
| EITNKVNSVIEK | 391 | 402 | 12 |  | + |  |  | + |  |  |  |
| ITNKVNSVIE | 392 | 401 | 10 |  | + |  |  | + |  | + | + |
| ITNKVNSVIEK | 392 | 402 | 11 |  | + |  |  | + |  | + |  |
| MNTQFTAVGKEFNH | 403 | 416 | 14 | + | + | + | + | + |  |  |  |
| NTQFTAVGKEFNH | 404 | 416 | 13 | + | + | + | + | + |  |  |  |
| FTAVGKEFNH | 407 | 416 | 10 |  | + |  |  |  |  |  |  |
| VGKEFNHLEKRIE | 410 | 422 | 13 |  |  |  |  | + |  |  |  |
| VGKEFNHLEKRIEN | 410 | 423 | 14 |  | + | + |  | + |  |  |  |
| VGKEFNHLEKRIENLN | 410 | 425 | 16 |  |  | + |  | + |  |  |  |
| GKEFNHLEKRIEN | 411 | 423 | 13 |  | + |  |  |  |  |  |  |
| KEFNHLEKRIEN | 412 | 423 | 12 |  | + |  |  | + |  |  |  |
| KEFNHLEKRIENLN | 412 | 425 | 14 |  |  |  |  | + |  |  |  |
| LEKRIENLNKKVD | 417 | 429 | 13 |  | + |  |  | + |  |  |  |
| LEKRIENLNKKVDD | 417 | 430 | 14 |  | + | + |  | + |  |  |  |
| LEKRIENLNKKVDDG | 417 | 431 | 15 | + | + |  | + | + |  |  |  |
| LEKRIENLNKKVDDGF | 417 | 432 | 16 |  | + | + | + | + |  |  |  |
| LEKRIENLNKKVDDGFL | 417 | 433 | 17 |  |  | + | + | + |  |  |  |
| EKRIENLNKKVDD | 418 | 430 | 13 | + | + |  |  | + |  |  |  |
| EKRIENLNKKVDDG | 418 | 431 | 14 | + | + | + | + | + |  | + | + |
| EKRIENLNKKVDDGF | 418 | 432 | 15 |  | + | + | + | + | + | + |  |
| EKRIENLNKKVDDGFL | 418 | 433 | 16 |  |  | + | + | + | + |  |  |
| KRIENLNKKVD | 419 | 429 | 11 |  | + | + |  |  |  | + |  |
| KRIENLNKKVDD | 419 | 430 | 12 |  | + |  |  | + |  |  |  |
| KRIENLNKKVDDG | 419 | 431 | 13 | + | + | + |  | + |  |  |  |
| KRIENLNKKVDDGF | 419 | 432 | 14 | + | + | + | + | + |  |  |  |
| KRIENLNKKVDDGFL | 419 | 433 | 15 |  |  | + | + | + |  |  |  |
| RIENLNKKVDD | 420 | 430 | 11 | + | + | + |  | + |  |  |  |
| RIENLNKKVDDG | 420 | 431 | 12 | + | + | + |  | + |  |  |  |
| RIENLNKKVDDGF | 420 | 432 | 13 | + | + |  |  |  |  |  |  |
|  |  |  |  |  |  |  |  |  |  |  |  |
