# Supplementary material for: Deciphering and predicting CD4+ T cell immunodominance of influenza virus hemagglutinin
Source: J Exp Med. 2020 Jul 9;217(10):e20200206. doi: 10.1084/jem.20200206 (PMC7537397; doi:10.1084/jem.20200206)
Supplement: Table S6 — lists H1-HA peptides identified by MS-based MHC-II peptidomics in donors HD2–HD4. [file JEM_20200206_TableS6.docx]

**Table S6.** H1-HA peptides identified by MS-based MHC-II peptidomics in donors HD2-HD4

| **Peptide sequence** | **Start^1^** | **End^1^** | **Length** |  | **HD2** |  | **HD3** | |  | **HD4** | |  |
| --- | --- | --- | --- | --- | --- | --- | --- | --- | --- | --- | --- | --- |
|  |  |  |  | HD2-EBV-B-rep1 | HD2-EBV-B-rep2 | HD2-EBV-B-rep3 | HD3-EBV-B-rep1 | HD3-EBV-B-rep2 | HD4-EBV-B-rep1 | HD4-EBV-B-rep2 | HD4-EBV-B-rep3 | HD4-EBV-B-rep4 |
| LSKSYINDKGKEV | 178 | 190 | 13 |  |  |  |  | +^2^ |  |  |  |  |
| KSYINDKGKEV | 180 | 190 | 11 |  |  |  | + |  |  |  |  |  |
| KSYINDKGKEVL | 180 | 191 | 12 |  |  |  |  | + |  |  |  |  |
| VEPGDKITFEATGNLVVPRYA | 251 | 271 | 21 | + | + |  |  |  |  |  |  |  |
| EPGDKITFEATGNLVVPRY | 252 | 270 | 19 | + | + |  |  |  |  |  |  |  |
| EPGDKITFEATGNLVVPRYA | 252 | 271 | 20 | + | + | + |  |  |  | + |  | + |
| VPRYAFAMERNAGSG | 267 | 281 | 15 |  |  |  | + |  |  |  |  |  |
| LPFQNIHPITIG | 307 | 318 | 12 | + | + |  |  |  | + |  |  |  |
| STKLRLATGLRNIPSI | 326 | 341 | 16 | + |  |  |  |  |  |  |  |  |
| STKLRLATGLRNIPSIQ | 326 | 342 | 17 | + | + | + |  |  |  |  |  |  |
| STKLRLATGLRNIPSIQSRG | 326 | 345 | 20 | + |  |  |  |  |  |  |  |  |
| LRLATGLRNIPSIQ | 329 | 342 | 14 |  |  | + |  |  |  |  |  |  |
| LATGLRNIPSIQSR | 331 | 344 | 14 |  |  |  |  |  | + |  |  |  |
| LATGLRNIPSIQSRG | 331 | 345 | 15 |  |  |  |  |  | + | + |  | + |
| LATGLRNIPSIQSRGL | 331 | 346 | 16 |  |  |  |  |  | + | + | + | + |
| LATGLRNIPSIQSRGLF | 331 | 347 | 17 |  |  |  |  |  | + | + |  | + |
| LATGLRNIPSIQSRGLFG | 331 | 348 | 18 |  |  |  | + | + | + | + | + | + |
| ATGLRNIPSIQSR | 332 | 344 | 13 |  |  |  |  |  |  | + |  |  |
| ATGLRNIPSIQSRG | 332 | 345 | 14 |  |  |  |  |  | + | + | + | + |
| ATGLRNIPSIQSRGL | 332 | 346 | 15 |  |  |  |  |  | + | + | + | + |
| ATGLRNIPSIQSRGLF | 332 | 347 | 16 |  |  |  |  |  | + | + |  |  |
| ATGLRNIPSIQSRGLFG | 332 | 348 | 17 |  |  |  |  |  | + | + | + |  |
| TGLRNIPSIQSRG | 333 | 345 | 13 |  |  |  |  |  | + | + | + | + |
| TGLRNIPSIQSRGL | 333 | 346 | 14 |  |  |  |  |  |  | + |  |  |
| TGLRNIPSIQSRGLF | 333 | 347 | 15 |  |  |  |  |  | + | + | + | + |
| TGLRNIPSIQSRGLFG | 333 | 348 | 16 |  |  |  |  |  | + | + | + | + |
| VNSVIEKMNTQFTAVG | 396 | 411 | 16 |  |  |  |  |  | + | + | + | + |
| VNSVIEKMNTQFTAVGK | 396 | 412 | 17 |  |  |  |  |  | + | + |  |  |
| SVIEKMNTQFTAVG | 398 | 411 | 14 |  |  |  |  |  | + | + |  | + |
| EEAKLNREEIDGVKLE | 508 | 523 | 16 |  |  |  |  |  |  | + |  |  |

1. Start and end position and length of the peptide residues in H1-HA.
2. + indicates identification of a peptide in an experiment replicate
